# Multi-Field Relaxation Dynamics of Parahydrogen-Hyperpolarized Propane Gas MRI Contrast Agent

**DOI:** 10.1021/acs.jpca.6c05094

**Published:** 2026-10-08

**Authors:** Clementinah Oladun, Nuwandi M. Ariyasingha, Anna Samoilenko, Md Raduanul H. Chowdhury, Zhongjie Shi, Kehuan Luo, Sidhartha Tan, Oleg G. Salnikov, Dudari B. Burueva, Alba Xhupi, Majd Suleiman, Jerome McCreadie, Larisa M. Kovtunova, Igor V. Koptyug, Juri G. Gelovani, Panayiotis Nikolaou, Jessica Ettedgui, Rolf E. Swenson, Kazutoshi Yamamoto, Norikazu Koyasu, Murali C. Krishna, Joseph W. Plummer, Adrienne E. Campbell-Washburn, Michael J. Barlow, Boyd M. Goodson, Eduard Y. Chekmenev

**Affiliations:** † Department of Biomedical Engineering, College of Engineering, Integrative Biosciences (IBio), Karmanos Cancer Institute (KCI), 2954 Wayne State University, Detroit, Michigan 48202, United States; ‡ Department of Pediatrics, Wayne State University, Detroit, Michigan 48202, United States; § 133879 International Tomography Center SB RAS, 3A Institutskaya st., Novosibirsk 630090, Russia; ∥ College of Medicine & Health Sciences, 11239 United Arab Emirates University, Al Ain, United Arab Emirates; ⊥ XeUS Technologies Ltd., Nicosia 2312, Cyprus; # Chemistry and Synthesis Center, 35035 National Heart, Lung, and Blood Institute, 9800 Medical Centre Drive, Rockville, Bethesda, Maryland 20850, United States; ¶ Center for Cancer Research, NCI, Building 10 B3B69, Bethesda, Maryland 20892, United States; ∇ Cardiovascular Branch, Division of Intramural Research, 2511 National Heart, Lung, and Blood Institute, National Institutes of Health, Bethesda, Maryland 20892, United States; ○ Sir Peter Mansfield Imaging Centre, 6123 University of Nottingham, Nottingham NG72RD, U.K.; ⧫ School of Chemical and Biomolecular Sciences, 2254 Southern Illinois University, Carbondale, Illinois 62901, United States

## Abstract

Hyperpolarized gas-phase MRI is an emerging diagnostic modality, with the clinical utility of hyperpolarized Xe-129 gas for functional lung imaging recently supported by FDA regulatory approval. However, the requirement for dedicated multi-nuclear hardware and complex, centralized production logistics remains a significant translational hurdle. Here, we investigate the longitudinal relaxation dynamics of hyperpolarized propane gas—a promising, proton-frequency alternative produced via heterogeneous parahydrogen-induced polarization (HET-PHIP)—across a wide range of magnetic field strengths: 0.35 T, 0.55 T, 3 T, and 7 T MRI scanners. In the imaging studies, gradient-echo MRI pulse sequence variants with small flip angle excitation radio-frequency pulses were utilized to record a series of scans to monitor the effective decay of the hyperpolarized state of propane gas in spherical plastic phantoms and excised rabbit lungs. We demonstrate that the effective lifetime (characterized by the constant of a mono-exponential signal decay *T*_d_) of hyperpolarized propane is strongly field-dependent, exhibiting a marked decrease from 1.51 ± 0.04 s (0.35 T) to 0.59 ± 0.02 s (0.55 T) to 0.54 ± 0.02 s (3 T) to 0.23 ± 0.01 s (7 T) in phantom studies using an RF flip angle of 5°. This trend is attributed to the presence of partially retained long-lived spin states at the low field of 0.35 T, which are quenched at higher field strengths as the spin system transitions from the strong-coupling to the weak-coupling regime. Moreover, the unfavorable spin-rotation dominated relaxation results in a further decrease of *T*_d_ at higher magnetic fields, especially evident at 7 T. Furthermore, we assess the relaxation behavior in excised rabbit lungs at 0.35 T, reporting a *T*_d_ value of 0.68 ± 0.12 s. This accelerated relaxation of hyperpolarized propane in the lung compared to phantom models is likely driven by susceptibility-induced dephasing and increased surface-to-volume interactions within the alveolar architecture. The results using hyperpolarized propane were also compared with imaging studies using hyperpolarized Xe-129 gas in the excised rabbit lungs at 0.35 T, yielding an effective ^129^Xe *T*_1_ of 11 ± 2 s. These results provide a fundamental biophysical characterization of hyperpolarized propane and underscore its potential as a highly accessible contrast agent for regional pulmonary function mapping.

## Graphical abstract

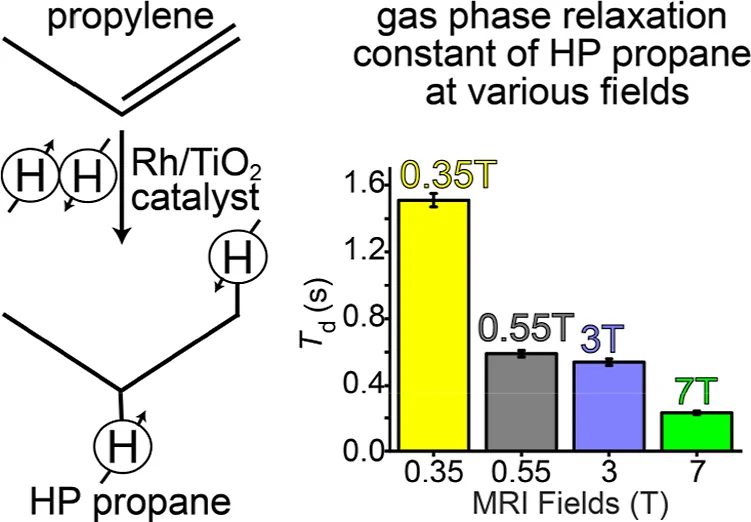

## Introduction

NMR hyperpolarization increases the nuclear spin polarization or the degree of nuclear spin alignment with the applied magnetic field up to the order of unity.^1–4^ This hyperpolarized (HP) state renders polarization increase by several orders of magnitude compared to the equilibrium (thermally polarized) level.^5–7^ Because NMR signal scales linearly with the nuclear spin polarization,^8^ such HP states also give rise to correspondingly massive increases of NMR signals,^3^ thus enabling new applications.^9–16^ In particular, HP gases (most notably HP ^129^Xe gas) allow for imaging of inhaled gas in the lungs.^17–22^ This technology enables functional pulmonary imaging because HP ^129^Xe can report on regional gas ventilation in the lungs, thus acting effectively as a novel MRI contrast agent.^17,18,23–27^ The use of HP ^129^Xe as a contrast agent for pulmonary imaging has been FDA-approved since 2022, and clinical utility is emerging as a result of that landmark regulatory approval.^28^

Despite the robust nature of HP ^129^Xe MRI for pulmonary disease diagnostics, the technology faces two translational barriers. First, the process of production requires expensive instrumentation ($0.4M+), which utilizes a relatively low-throughput process (30+ min per clinical batch).^29–38^ Second, the process of MRI scanning of the HP ^129^Xe contrast agent requires a multi-nuclear capability, while nearly all current clinical MRI scanners are configured to scan only protons (i.e. ^1^H spins). The multi-nuclear scanners' upgrades have high cost ($0.5M) and additionally render complex logistics, making such scanners available only at a few privileged sites.

An alternative technology has recently emerged for the production and MRI scanning of HP gas media.^39–46^ This technology relies on pairwise parahydrogen (*p*-H_2_) addition to a suitable molecular precursor, resulting in the incorporation of the nascent *p*-H_2_ in an asymmetric molecule.^47,48^ For example, pairwise *p*-H_2_ addition to propylene creates HP propane, Figure 1a.^49^ The process of production is typically established over a heterogeneous catalyst, allowing the rapid conversion of *p*-H_2_ and propylene into HP gaseous product, free from the catalyst contamination.^49^ The process of clinical-scale production of HP propane has been demonstrated recently,^50–52^ and validated in an animal model of excised lungs,^50,53^ clearly demonstrating the feasibility of obtaining ventilation lung imaging using this technology. Moreover, this technology has been expanded to the production of HP butane and HP diethyl ether.^54–57^ The process of production is fast (∼1 s) and inexpensive: in fact, a disposable hyperpolarizer design has also been realized.^53^ Furthermore, because the HP states within these novel contrast agents are comprised of proton spins, they can be readily scanned on conventional proton-only clinical MRI scanners—making it possible (in principle) to scan such HP media on virtually any clinical MRI scanner.^50^

**Figure 1:**
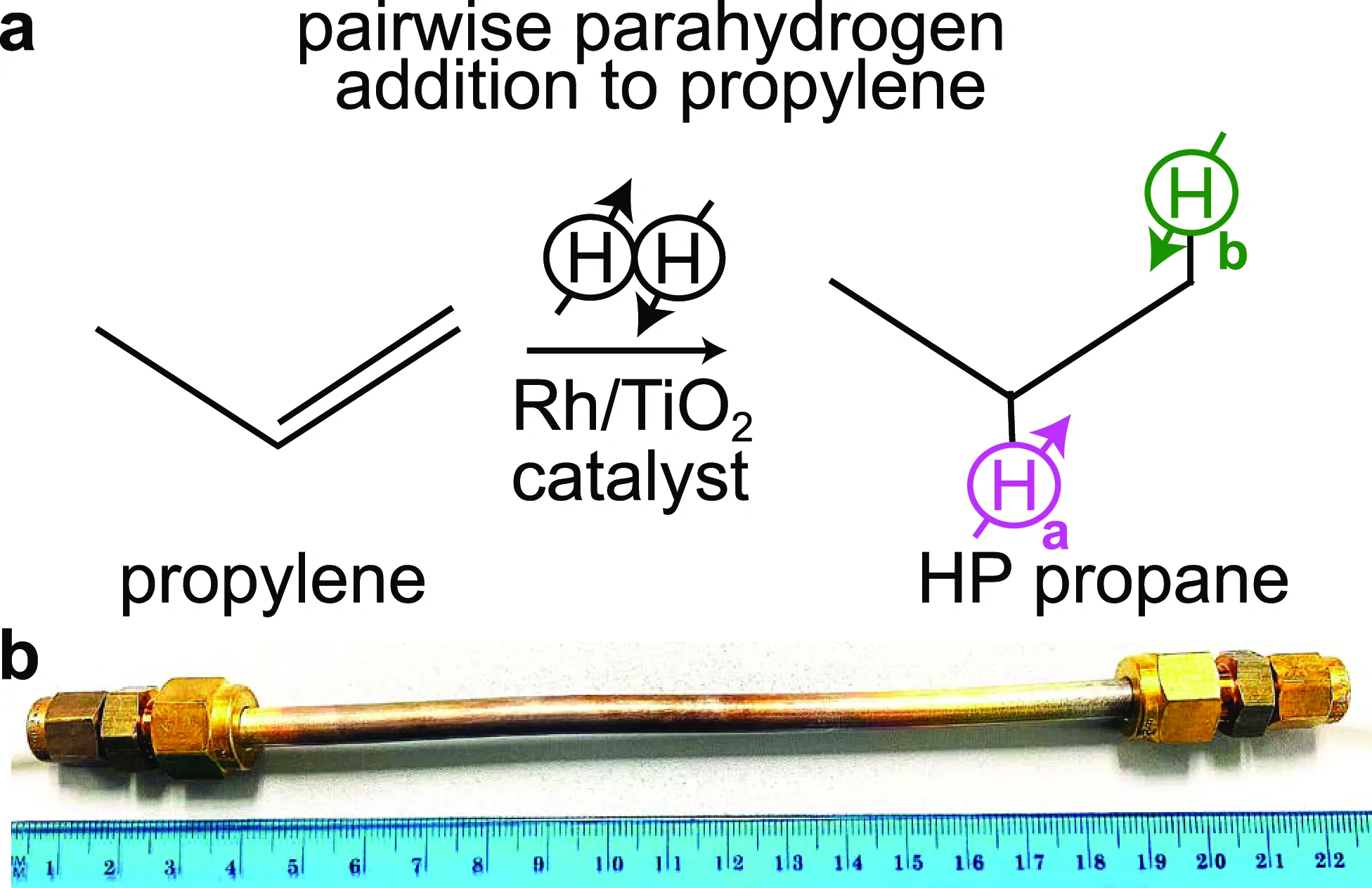
(a) Schematic showing the pairwise addition of *p*-H_2_ gas to propylene over a heterogeneous Rh/TiO_2_ (1 wt % metal loading) catalyst to yield HP propane gas. b) Closeup photo of the copper-tube-based reactor, in which the heterogeneous catalyst and copper beads are packed to perform pairwise parahydrogen addition to propylene (copper beads are added to improve heat management due to their much better thermal conductivity than that of TiO_2_ support).

However, molecular gases generally have relatively short nuclear *T*_1_ times; correspondingly their HP state decays rather quickly. As a result of this limitation, the production of proton-hyperpolarized gas contrast agents must be performed in close proximity of the MRI scanner. Realizing that the *T*_1_ decay of the HP state is the limiting factor for this new alternative technology of HP gas MRI, this work investigates the dependence of the HP state lifetime on the strength of the MRI scanner magnetic field. Our rationale builds on the previous reports of long-lived spin states (LLSS) observed at the magnetic field regime corresponding to strong spin–spin coupling, i.e., the condition when the spin–spin coupling between the nascent parahydrogen-derived protons (Figure 1a) is greater than the difference of their chemical shifts. Specifically, we investigate the relaxation dynamics of the HP state at the clinically relevant condition of 1 atm pressure using 0.35, 0.55, 3, and 7 T MRI scanners and a 1.4 T benchtop NMR spectrometer.

## Materials and Methods

### Production of HP Propane Gas

HP propane used in this study was produced via the heterogeneous parahydrogen-induced polarization (HET-PHIP) technique. *p*-H_2_ with over 95% para state is produced from ultra-pure hydrogen gas (>99.999%, Airgas).^58^ The ca. 1:1 propylene:*p*-H_2_ gas mixture is prepared in an aluminum tank (∼0.7 L). First, the tank is flushed several times with propylene to remove any residual gases from a previous use. Thereafter, propylene is filled into the tank to the desired pressure (typically ∼70 psig). *p*-H_2_ gas is then added in a slight (∼5%) excess to propylene (typical total pressure of the mixture was 145 psig). The pairwise addition of *p*-H_2_ to propylene over the Rh/TiO_2_ catalyst results in the symmetry breaking of the NMR-inactive *p*-H_2_ molecule and subsequent incorporation of the singlet spin order from the *p*-H_2_-derived protons to the product propane molecule. This transformation is schematically shown in Figure 1a with H_a_ and H_b_ sites denoting the symmetry breaking due to their different chemical shifts by ∼0.45 ppm.^40,41,49^ As a result, the HP propane gas can be readily detected via NMR/MRI.

The Rh/TiO_2_ catalyst comprises ∼1 nm particles of Rh metal deposited on the titania support surface (∼1 wt % loading).^59^ The catalyst is packed into a 0.25-inch outer diameter (OD) copper tube (McMaster-Carr, P/N 5174K21), which is about 20 cm in length (Figure 1b). The catalyst (0.2 g) is loaded with copper beads (∼17 g, Sigma-Aldrich, 266086-2KG, 425 μm particle size) to improve temperature and heat management during the exothermic hydrogenation reaction that yields HP propane.^57^ The ends of the reactor are capped with glass wool to prevent particles from migrating out of the reactor region, and are mated with brass reducing connectors (McMaster-Carr, P/N 5272K291) to connect the reactor with 1/8-inch-OD Teflon tubing (McMaster-Carr, P/N 5239K24). Considering the high flammability of propane and hydrogen gases, the exhaust of the phantom lines was kept far away (more than 5 m) from the electronics and other equipment. Moreover, sufficient amount of time was employed between the experiments to ensure for gas evacuation and dilution to stay several orders of magnitude below the lower explosivity limit (LEL) for these gases (∼2%).

### Experimental Setup for Gas-phase Relaxation Studies of HP Propane in a 1.4 T Benchtop NMR Spectrometer

The setup for the relaxation dynamics of HP propane gas in a 1.4 T NMR spectrometer has been reported previously.^57^ Briefly, a 1/8-inch-OD Teflon tube is inserted into the bore of the 1.4 T NMR spectrometer (SpinSolve, Magritek or NMR Pro, Nanalysis). HP propane gas then flows through the tube for about 2–3 s, and then is quickly stopped to enable NMR acquisitions while the gas is still in its HP state. After the gas flow is stopped, a pseudo-2D NMR acquisition is performed to measure the decay of the signals from the HP gas in the Teflon tube at 1 atm. The sequence for data acquisition is started before the HP gas flow is stopped to capture the full extent of the relaxation dynamics. This data is analyzed using a custom-built MATLAB code to evaluate a mono-exponential decay constant for the acquired NMR signals.^53,57^

### Experimental Setup for Gas-phase Relaxation Imaging Studies of HP Propane in a Hollow Phantom Sphere in MRI Scanners

Relaxation dynamics of HP propane gas in MRI scanners were studied as follows. The tank with the gas mixture prepared as described above is connected via a 1/8-inch-OD Teflon tubing to the catalytic reactor. The reactor is connected to a manual shut-off valve (IDEX, P/N P-783) through a Teflon tube, which in turn is connected to the phantom hollow plastic sphere (McMaster-Carr, P/N 9338K13, approximately 18 mL inner volume), which sits inside a detection coil in the MRI scanner (Figure 2a). The gas mixture tank valve is opened, and the precursors’ mixture is allowed to flow through the reactor. The manual valve (shown in blue) is opened to displace the air and fully fill the phantom with fresh HP gas in less than 1 s at a typical HP propane flow rate of 4–7 standard liters per minute, SLM; thereafter both valves are closed after about 2 s. The imaging sequence is initiated on the MRI scanner before the gas flow is started to capture the arrival of the HP propane gas, as well as the decay of the gas NMR signal in the phantom. This approach ensures complete recording of the HP gas relaxation dynamics and allows accurate measurements of signal changes across different magnetic fields.

**Figure 2:**
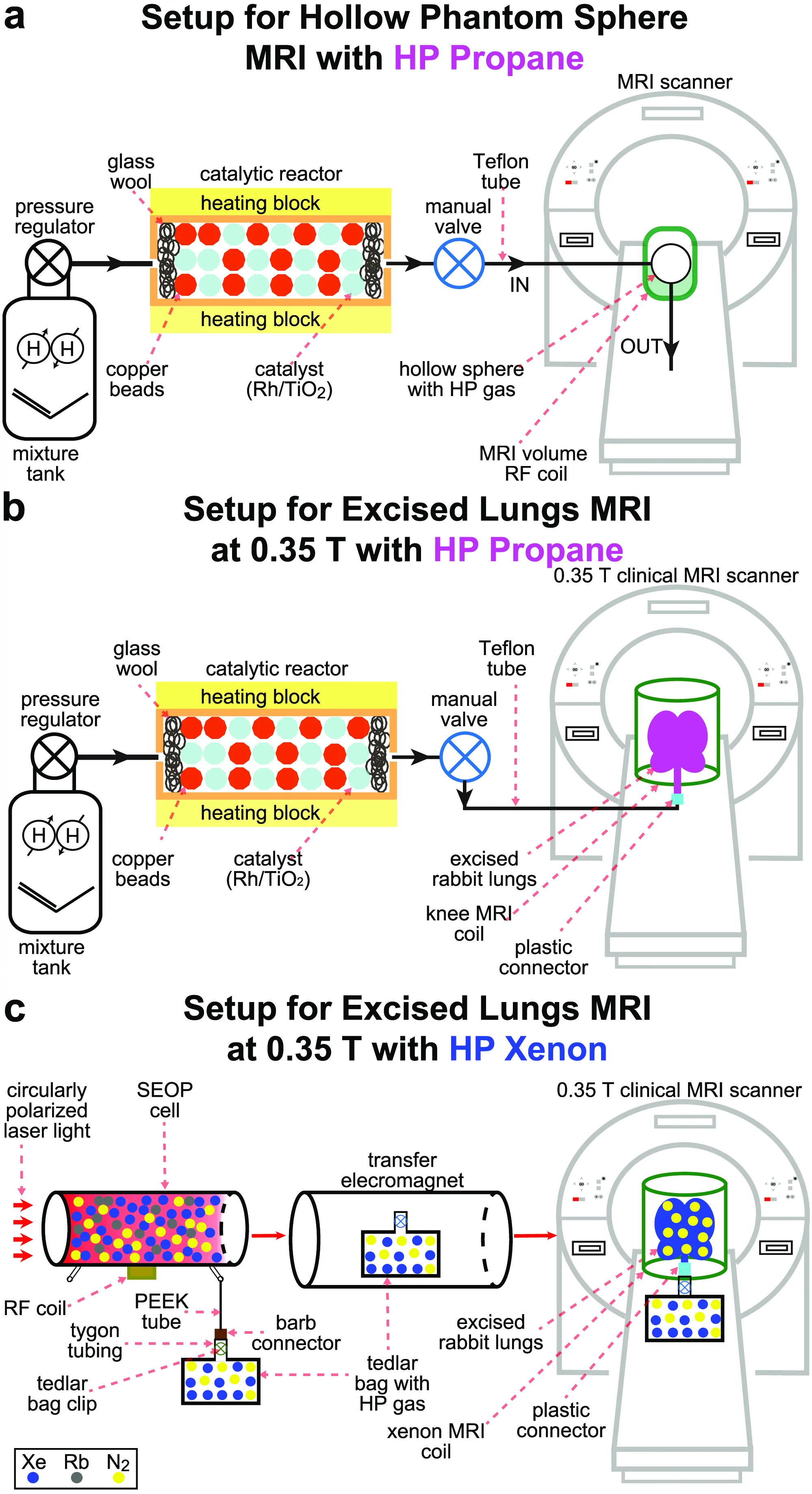
(a) Schematic depiction of the experimental setup used for relaxation studies in the hollow phantom sphere within different MRI scanners. (b) Schematic depiction of the setup for relaxation studies in the excised rabbit lungs in a 0.35 T clinical MRI scanner showing the storage gas mixture tank containing parahydrogen gas and propylene in a ∼1:1 ratio. The pressure regulator on the tank providing control over the inlet gas pressure is connected to the catalytic reactor, which contains the HET-PHIP catalyst. The manual valve helps to control the inflation of the phantom or excised rabbit lungs. (c) Schematic depiction of the setup for relaxation studies of HP ^129^Xe gas in excised rabbit lungs. The HP ^129^Xe produced in the hyperpolarizer is ejected in a Tedlar bag, which is stored in the protective field of an electromagnet during the bag transfer from the production facility to the MRI scanning facility. The Tedlar bag is connected to the lung trachea and the lungs are inflated via bag compression and opening the connecting tube via release of a plastic clip.

Essentially the same imaging sequences (variants of a 2D gradient echo sequence (GRE) with excitation flip angle (FA) of 1°, 2°, 3°, and 5°) were utilized on four MRI scanners, and the image encoding matrices were similar (64 × 64 for 0.35 T (Time Medical) and 0.55 T (Siemens Healthineers), 64 × 40 for 3 T (Bruker) and 32 × 32 for 7 T (Bruker)), see Supporting Information for details.

The GRE sequence used on the 0.35 T clinical MRI scanner consists of 16 back-to-back acquisitions performed with the following imaging parameters: field of view (FOV): 256 × 256 mm^2^; slice thickness: 50 mm. The total imaging time is ∼18–19 s for all 16 repeat scans, resulting in the effective repeat scan time of 1.1–1.2 s per each 2D image (there was virtually no delay between the repeat scans).

On the 0.55 T MRI scanner, the GRE-based sequence utilized: FOV: 320 × 320 mm^2^; slice thickness: 8 mm. The total imaging time was programmed at 147 s for 499 repeat scans, resulting in the repeat scan time of 0.293 s per each 2D image.

A GRE-based sequence was also used for image acquisition on the 3 T MRI scanner, with the following imaging parameters: FOV: 64 × 64 mm^2^; slice thickness: 15 mm; and the total imaging time: 9.4 s for 64 back-to-back repetition scans, resulting in the repeat scan time of 0.147 s per each 2D image.

On the 7 T MRI scanner, GRE sequence parameters included: FOV: 96 × 96 mm^2^; zero-filled matrix: 64 × 64; and slice thickness: 10 mm. The total imaging time was programmed at 9.25 s for 32 repeat scans, resulting in the repeat scan time of 0.289 s per each 2D image (see Supporting Information for details).

### Gas-phase Spectroscopic *T*_1_ Relaxation Studies of Thermally Polarized Propane Using Inversion-Recovery RF Pulse Sequence

A 5 mm Young’s NMR tube was thoroughly evacuated to eliminate gaseous oxygen. After that, neat propane gas (99.5% purity) was filled into the tube under ambient atmospheric pressure. The proton *T*_1_ relaxation times of thermally polarized propane were measured using the standard inversion-recovery RF pulse sequence across a broad range of magnetic field strengths using four different NMR spectrometers: a Bruker AV 300 SB (^1^H Larmor frequency of 300 MHz, corresponding to 7.05 T), a Bruker Avance III 400 (400 MHz, 9.4 T), a Bruker Avance III HD 700 (700 MHz, 16.4 T), and a Spinsolve 60 Nitrogen Ultra (60 MHz, 1.4 T). All experiments were conducted at 23 °C. For the estimation of *T*_1_, the propane H_a_ and H_b_ signal integrals (see the notation in Figure 1a) were fitted to the equation *I* = *I*_0_·(1-*A*·exp­(-*t*/*T*_1_)).

### Experimental Setup for Gas-phase Relaxation Imaging Studies of HP Propane in Excised Lungs

The relaxation dynamics of HP propane gas in excised rabbit lungs was also studied at 0.35 T. The setup for this study was similar to that described above with the exception of the use of excised rabbit lungs, which were arranged to reside in a knee MRI coil in a 0.35 T MRI scanner (Figure 2b). Imaging parameters were the same as described for the 0.35 T phantom studies above.

Excised rabbit lungs used for these imaging studies were obtained from other IACUC-approved studies from the Division of Laboratory Animal Resources at Wayne State University.^60–65^ The lungs were checked by first inflating with air, which helps to see how well the lungs maintain their integrity throughout inflation. If by any chance, a given pair of lungs were found to leak significantly, they were discarded and not used for the study. On the other hand, if the lungs are found to hold their inflation well, they can be potentially used for a large number of inflation runs (up to ∼40) and can even be kept in the refrigerator (∼5 °C) to be re-used the next day. Also, to keep them hydrated and help maintain their longevity throughout the imaging process, the lungs are sprinkled with water every 0.5–1 h.

### Experimental Setup for Gas-phase Relaxation Studies with HP ^129^Xe

^129^Xe gas was produced using a 50:50 Xe/N_2_ gas mixture using a generation-3 (GEN-3) clinical scale batch mode hyperpolarizer.^19,35,66–68^ The gas was produced and ejected into a Tedlar bag and was transported to the MRI facility about 15 min away from the site of HP gas production using a transfer electromagnet (*B*_0_ = 3–4 mT).^69^ The HP ^129^Xe gas stored in the Tedlar bag was quickly transferred from the transport electromagnet into the magnetic field of the 0.35 T MRI scanner to avoid polarization losses.^69^ The Tedlar bag was then connected to the excised rabbit lungs in the MRI scanner through a 1-inch Teflon tube (Figure 2c). Just as with the HP propane phantom studies, the imaging sequence was initiated before the gas was injected into the excised lungs for about 2–3 s, and the gas flow was then stopped. The imaging sequence consisted of 16 back-to-back repeat scans and the imaging parameters were the same as outlined above for the phantom and excised rabbit lungs with HP propane in the 0.35 T MRI scanner. A clinical chest ^129^Xe RF coil was utilized for detecting the MRI signal from the HP gas, whereas a large (∼0.5 m^3^) transmit ^129^Xe RF coil (integrated in the pole plates of 0.35 T permanent magnet) was utilized to deliver RF excitation pulses using a 4 kW RF amplifier. All components within the ^1^H and ^129^Xe transmit-receive RF chains were utilized as developed by the vendor (Time Medical) without any custom modification.

## Results and Discussion

### Relaxation Dynamics of HP Propane Gas in a Hollow Phantom Sphere

2D images were acquired across four MRI scanners, complemented by spectroscopic data from a 1.4 T NMR spectrometer (see Supporting Information), to investigate the relaxation dynamics of HP propane gas at a clinically relevant pressure of 1 atm. This wide range of magnetic field strengths allowed for a systematic evaluation of depolarization mechanisms across typical clinical and high-field regimes.

As described in the Methods section, the scanning sequence was initiated before the HP propane gas entered the phantom sphere located within the detection region; the gas flow was subsequently stopped to capture static decay dynamics. Figure 3a displays a characteristic HP propane signal decay profile within the phantom sphere at 0.35 T. The first image in this series corresponds to the exact frame captured immediately after gas flow cessation. The rationale for timing the image acquisition and data analysis with flow cessation is to ensure that no fresh HP gas enters the detection region during the measurement, while also mitigating potential imaging artifacts induced by active gas flow. Using this baseline and the subsequent sequential images, the signal-to-noise ratio (SNR) was computed for each frame. A mono-exponential fit was applied to a designated region of interest (ROI) to determine the effective relaxation constant for each magnetic field strength. For simplicity, this effective relaxation constant is denoted as *T*_d_ to explicitly emphasize magnetization depolarization due to RF pulsing, potential presence of the LLSS at low fields,^70,71^ and *T*_1_ decay. Illustrative examples of *T*_d_ measurements in Figure 3a,b yield the following values: 1.54 ± 0.07 s at 0.35 T, 0.61 ± 0.06 s at 0.55 T, 0.54 ± 0.02 s at 3 T and 0.24 ± 0.01 s at 7 T.

**Figure 3:**
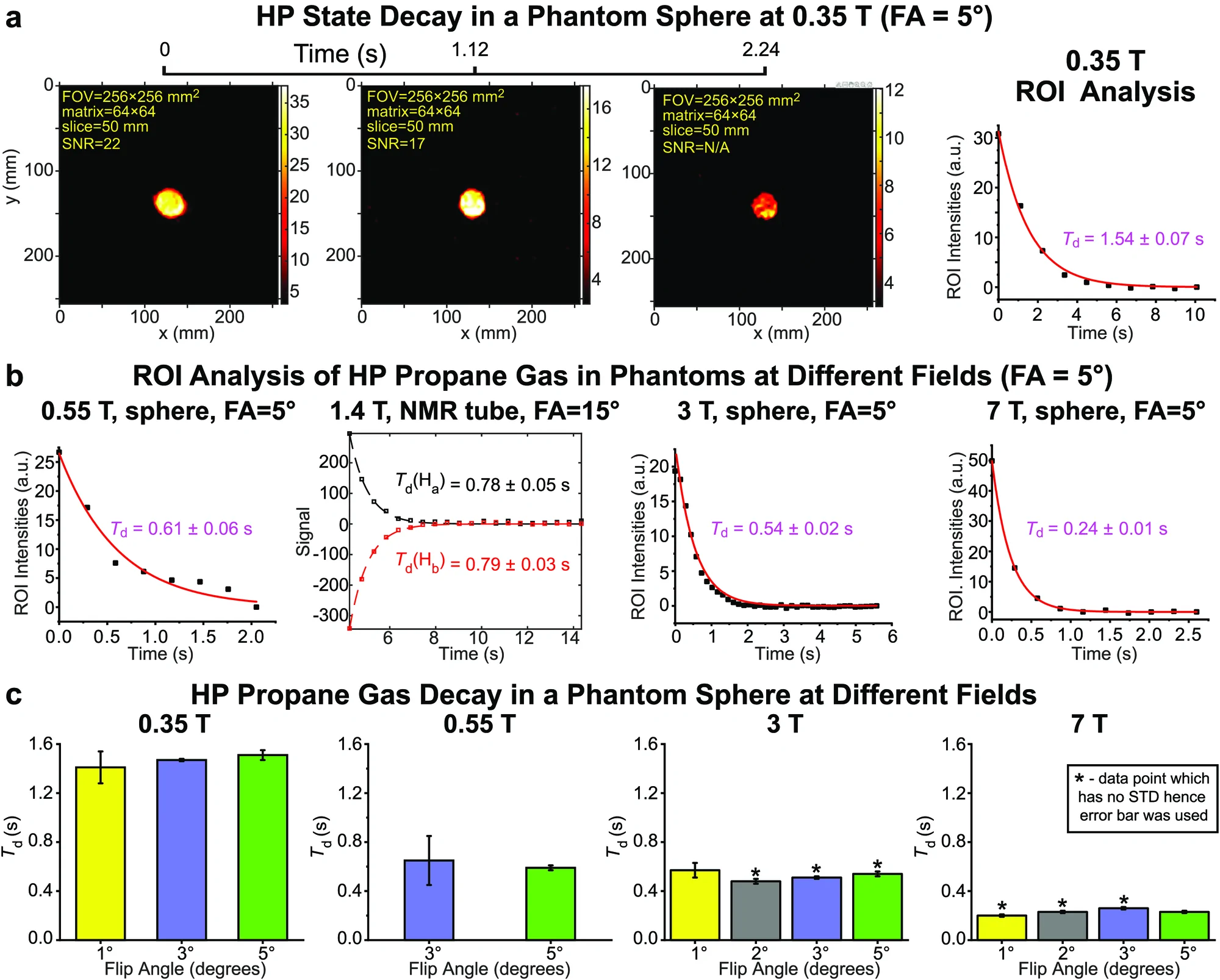
(a) Back-to-back repeat scan imaging series of HP propane in a hollow phantom sphere in a 0.35 T MRI scanner at 1 atm. The imaging parameters are FOV = 256 × 256 mm^2^, matrix 64 × 64, slice thickness = 50 mm, *F*_A_ = 5°, *T*_R_ = 17.19 ms, *T*_E_ = 8.21 ms, total imaging time = 18 s. The first three images in the series after injecting the HP gas into the phantom are shown here. A mono-exponential fitting of the HP propane signal across the whole image series was plotted to obtain a decay constant of 1.54 ± 0.07 s. b) Representative mono-exponential fittings of HP propane signals at 1 atm at 0.55 T, 1.4 T, 3 T, and 7 T. c) Histogram plot showing the decay constant values and their standard deviations (STD, obtained via multiple measurements) at different flip angles ranging between 1° and 5° at 0.35 T, 0.55 T, 3 T, and 7 T, respectively. At 0.35 T, decay constant values and STDs were 1.41 ± 0.14 s (flip angle (FA) = 1°), 1.47 ± 0.01 s (FA = 3°), and 1.51 ± 0.04 s (FA = 5°). At 0.55 T decay constant values and STDs were 0.65 ± 0.20 s (FA = 3°), and 0.59 ± 0.02 s (FA = 5°). At 3 T, decay constant values and STD were 0.57 ± 0.06 s (FA = 1°). Moreover, at 3 T, the decay constant and the associated error bar (n = 1 measurement) were 0.48 ± 0.02 s (FA = 2°), 0.51 ± 0.01 s (FA = 3°), and 0.54 ± 0.02 s (FA = 5°). At 7 T, decay constant values and the experimental error bars (*n* = 1 measurement) were 0.20 ± 0.01 s (FA = 1°), 0.23 ± 0.01 s (FA = 2°), and 0.26 ± 0.01 s (FA = 3°). Moreover, the decay constant value and STD values were 0.23 ± 0.01 s (FA = 5°) at 7 T. See Supporting Information for details.

Moreover, 2D images were acquired at various flip angles ranging between 1° and 5° at the different magnetic fields of 0.35, 0.55, 3, and 7 T to confirm that the small-angle pulses employed in the imaging sequence do not act as a major source of HP state decay. In these studies (shown in Figure 3c), the measurement was repeated multiple times, so the reported *T*_d_ values are accompanied by the standard deviation (STD) unless otherwise noted. Indeed, these measurements yielded essentially the same value of *T*_d_ for each of the fields studied, irrespective of the excitation flip angle (FA) utilized. For example, at 0.35 T, decay constant values and STDs were 1.41 ± 0.14 s (FA = 1°), 1.47 ± 0.01 s (FA = 3°), and 1.51 ± 0.04 s (FA = 5°).

A comparison of the *T*_d_ values across the fields studied reveals a striking decrease in the decay constant with increasing main magnetic field strength *B*_0_. The substantially longer *T*_d_ lifetime observed at 0.35 T is rationalized by the presence of partially preserved LLSS,^52^ which occur because the chemical shift difference between the H_a_ and H_b_ protons (0.45 ppm, corresponding to ca. 6.7 Hz at 0.35 T) is comparable to the scalar spin–spin coupling (^3^ *J*_H–H_ ∼7 Hz) between these two nuclei at this specific low magnetic field regime.^71^ Under these conditions, the singlet-triplet configuration is partially protected against certain symmetric relaxation pathways.^72^

As the external magnetic field scales upward into the clinical (1.4 and 3 T) and high-field (7 T) regimes, this strong-coupling condition is lost (Δν > *J*), lifting the protection of the LLSS and accelerating standard gas-phase relaxation. This field-dependent transition may be further compounded by the intrinsic spin-rotation (SR) interaction, which typically dominates the longitudinal relaxation of small volatile hydrocarbons like propane via collision-induced rotational magnetic fields.^73,74^ Additionally, macroscopic field inhomogeneities across the phantom geometry and local magnetic susceptibility gradients may become significantly more pronounced at higher field strengths. These variations enhance effective depolarization and transverse dephasing during multi-pulse readouts,^75,76^ working in tandem with the loss of LLSS protection to drive the steep reduction in the effective gas-phase hyperpolarization lifetime from ∼1.5 s at 0.35 T down to ∼0.25 s at 7 T.

To get a further insight into the dependence of HP propane lifetime on the external magnetic field, NMR spectroscopic measurements of *T*_1_ relaxation times of thermally polarized propane gas were conducted at magnetic fields of 1.4, 7.05, 9.4, and 16.4 T using the standard inversion-recovery pulse sequence (Figure S7). In contrast to the MRI *T*_d_ data shown in Figure 3c, no clear trends were observed. In particular, the shortest *T*_1_ values of ca. 0.6 s were obtained at 1.4 T, while the longest *T*_1_ were observed at 7.05 T (ca. 0.73 s for H_a_ and ca. 0.78 s for H_b_). Higher fields of 9.4 and 16.4 T provided similar *T*_1_ values of ca. 0.67 s for H_a_ and ca. 0.73 s for H_b_. However, the differences between all these *T*_1_ values at various magnetic fields were not as strong as the differences in *T*_d_ obtained from the ROI analyses of MR images. These results indicate that the decrease of *T*_d_ with magnetic field apparently is connected to other factors outlined above rather than to the spin-rotation mechanism governing *T*_1_ relaxation.

The observed magnetic-field dependence can be further evaluated by considering both the spin-coupling regime and potential experimental contributions to the measured *T*_d_. Quantitatively, using ^3^ *J*_H–H_ ∼ 7 Hz, the ratio of the chemical-shift frequency separation to scalar coupling, Δν/*J* increases from approximately 1 at 0.35 T to ∼1.5 at 0.55 T, ∼8 at 3 T, and ∼19 at 7 T. Thus, over the experimental field range, the propane spin system evolves from the strong/intermediate-coupling regime toward the weak-coupling regime, providing a physically consistent basis for progressively reduced preservation of coupled long-lived spin order with increasing field. Nevertheless, the *T*_d_ determined from the imaging experiments represents an effective HP image-signal decay constant rather than a measurement of a single intrinsic molecular relaxation process. Contributions from *T*_2_*, static-field inhomogeneity, diffusion through background gradients, and scanner-specific acquisition conditions cannot therefore be quantitatively separated from the present multi-scanner data. Importantly, the essentially unchanged *T*_d_ values obtained using different low flip angles at each magnetic field (Figure 3c) argue against RF-induced depletion as the dominant origin of the observed field dependence. Together with the comparatively weak field dependence of the independently measured thermal *T*_1_ values, these observations support preservation of coupled long-lived spin order as a mechanistically consistent interpretation of the enhanced low-field lifetime, while not excluding additional contributions from dephasing, diffusion, and differences between the MRI systems.

Despite the large polarization enhancement of HP propane relative to thermally polarized protons at 0.35 T, the much greater proton density of tissue water results in a detectable background signal. Indeed, a tissue-water background is visible in the excised-lung propane images (see Supporting Information). The background does not preclude visualization of HP propane within the lung because the HP gas produces a strong transient signal localized primarily within the airspaces. Furthermore, the HP propane and tissue-water contributions have markedly different temporal behavior: the propane signal decays rapidly following administration, whereas the equilibrium tissue-water background remains comparatively constant. This difference provides a straightforward means for separating the HP-gas contribution using pre-inhalation reference imaging, background subtraction, or temporal analysis. Frequency-selective excitation and water-suppression approaches could provide additional discrimination in future implementations.

At even lower magnetic fields, where the chemical-shift frequency separation becomes substantially smaller than the scalar coupling (Δν ≪ ^3^ *J*_H–H_), propane enters the strongly coupled regime.^71^ Under these conditions, RF irradiation should be considered not only in terms of conventional homonuclear decoupling but also as a means of manipulating or preserving long-lived coupled-spin order.^71^ For example, at 0.05 T the expected proton chemical-shift separation is approximately 1 Hz, compared with a scalar coupling of approximately 7 Hz, while the proton Larmor frequency is only ∼2.1 MHz. This regime may therefore permit low-power spin-locking or related RF schemes^70,77^ with substantially reduced specific absorption rate (SAR) constraints.^78^ Depending on the RF parameters, such approaches could potentially preserve or interconvert hyperpolarized magnetization and long-lived spin order, thereby extending the useful imaging window. Conversely, RF irradiation could perturb the strong-coupling interactions responsible for the favorable low-field relaxation behavior, and experimental optimization will therefore be required.

### Comparative Gas-Phase Relaxation Dynamics of HP Propane and HP ^129^Xe in Excised Rabbit Lungs in a Clinical 0.35 T Open-MRI Scanner

Motivated by a comparatively higher *T*_d_ value of HP propane in phantoms at 0.35 T, the relaxation dynamics studies in excised rabbit lungs were performed using this MRI scanner, Figure 4a. 3D MR images were acquired with HP propane (*P* ∼ 1%) gas as well as HP ^129^Xe (50:50 mixture with N_2_ gas, *P* ∼ 10%) using the same imaging parameters, including FOV of 256 × 256 mm^2^, imaging matrix of 64 × 64, FA of 5°, the same number of 16 back-to-back scan acquisitions, and similar TR/TE values (see Supporting Information for details). The examples of pseudo-2D MRI scanning of an HP gas bolus injected in the rabbit lungs and the corresponding signal decay curves are shown in Figure 4a (HP propane) and Figure 4b (HP ^129^Xe). The experiments with HP propane were performed using FA of 1°, 2°, 3° and 5° multiple times in multiple lungs to account for biological variability. The summary of the results (Figure 4a) reveals that different FA angles did not affect the measured *T*_d_, indicating that the depolarization due to application of RF pulses were likely not the dominant relaxation mechanism. Moreover, the relaxation constant *T*_d_ for HP propane gas was 0.68 ± 0.12 s (FA = 5°, Figure 4a), which is substantially shorter than the corresponding *T*_d_ value of 1.51 ± 0.04 s in the phantom (Figure 3c).

**Figure 4:**
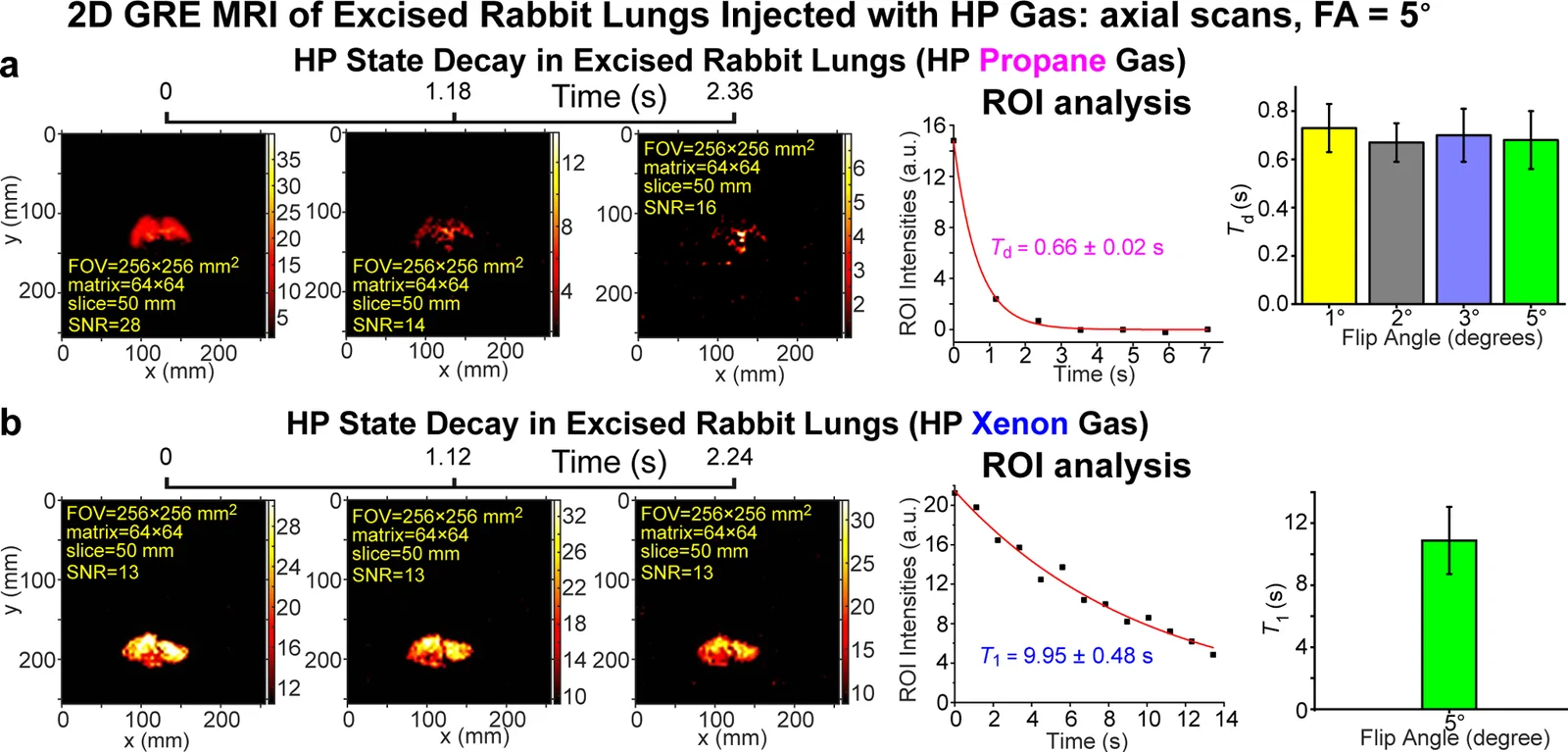
(a) Back-to-back imaging series showing the decay of the signal of HP propane gas inflated in excised rabbit lungs at 0.35 T. The first three images of the series after the lungs were fully inflated are shown. Example of one mono-exponential fitting data set of HP signal, yielding an effective relaxation constant *T*_d_ of 0.66 ± 0.02 s. A histogram showing the *T*_d_ values at different flip angles as well as standard deviations (error bars) of multiple repeat experiments for each FA value. Decay constant value and STD were 0.73 ± 0.10 s (FA = 1°), 0.67 ± 0.08 s (FA = 2°), 0.70 ± 0.11 s (FA = 3°), and 0.68 ± 0.12 s (FA = 5°). (b) Back-to-back imaging series showing the decay of the signal of HP ^129^Xe gas inflated in excised rabbit lungs at 0.35 T. The first three images of the series after the lungs were fully inflated are shown. Example of one mono-exponential fitting data set of HP signal, giving a relaxation time of 9.95 ± 0.48 s. A histogram showing the ^129^Xe *T*_1_ value and standard deviation of multiple repeat experiments for FA = 5° with a value of 10.9 ± 2.2 s. The same imaging parameters were used for both series shown in panels a) and b): FOV = 256 × 256 mm^2^, matrix 64 × 64, slice thickness = 50 mm, FA = 5°, TR = 17.52 ms, TE = 8.54 ms, total imaging time = 18 s.

Thus, propane provides a short, higher-sensitivity imaging window suitable for rapid proton-frequency ventilation imaging, whereas Xe provides a much longer temporal window suitable for repeated acquisitions and more elaborate protocols.

This accelerated relaxation behavior can be potentially rationalized by two primary compounding biophysical mechanisms characteristic of the lung microenvironment, which have been previously seen in relaxation dynamics studies with HP propane-*d*_6_.^52^ First, the complex, highly branched alveolar lung structure may introduce severe magnetic susceptibility mismatches at the numerous gas–tissue interfaces. At 0.35 T, these gradients may induce micro-scale field inhomogeneities. As the HP gas molecules undergo rapid restricted diffusion within the alveolar spaces, they traverse these local gradients, which drastically accelerates transverse relaxation and significantly contributes to the decrease of the effective LLSS lifetime *T*_LLSS_, which is observed as the reduction of the apparent *T*_d_ value. Second, unlike the inert surfaces of a hollow plastic sphere, the complex biological surface of the pulmonary epithelium presents a vastly higher surface-area-to-volume ratio alongside potential interactions with microscopic cellular environments or residual dissolved paramagnetic O_2_ in the tissues. Collisions with the alveolar walls enhance both dipole–dipole interactions and spin-rotation relaxation pathways, effectively acting as an efficient sink for hyperpolarization.

A direct empirical comparison of image SNR between HP propane and clinical HP ^129^Xe pulmonary protocols cannot be strictly made here due to several substantial confounding hardware and experimental factors. First, the HP ^129^Xe contrast gas mixture is typically heavily diluted with an inert gas buffer (e.g., a 1:1 ratio with N_2_), whereas HP propane is delivered essentially undiluted. Second, according to fundamental electromagnetic reciprocity principles governing NMR detection,^8^ the small, highly optimized, close-fitting proton knee coil utilized for our excised rabbit lungs setup inherently possesses a vastly higher intrinsic filling factor and geometric sensitivity compared to the large, spacious clinical chest coil that was utilized for human ^129^Xe torso imaging and repurposed for the smaller rabbit lung sample. Rather than relying on hardware-dependent image SNR metrics, a more objective comparison rests on the relaxation dynamics. Indeed, HP ^129^Xe exhibits a much longer hyperpolarization lifetime in the lungs’ environment with effective *T*_1_ = 10.9 ± 2.2 s in comparison to *T*_d_ = 0.68 ± 0.12 s for HP propane. At the same time, the reader is reminded about the low gyromagnetic ratio γ_129Xe_ of ∼ 0.27 times that of the proton, which severely penalizes ^129^Xe intrinsic signal detection efficiency. Conversely, HP propane leverages the maximum possible biomedically relevant gyromagnetic ratio, meaning that even with an reduced, sub-second effective lifetime, the significantly higher signal per spin enables rapid, sub-second MRI-based data acquisition strategies. Consequently, while ^129^Xe remains the gold standard for steady-state breath-hold functional or diffusion mapping, HP propane may serve as a highly complementary, ultra-accessible proton-frequency alternative capable of mapping regional ventilation dynamics using standard, unmodified clinical hardware.

### Comparative Gas-Phase MR Sensitivity of HP Propane and HP ^129^Xe in Excised Rabbit Lungs in a Clinical 0.35 T Open-MRI Scanner

A quantitative comparison of HP propane and HP ^129^Xe image sensitivity requires consideration of spin concentration, polarization, gyromagnetic ratio, resonance frequency, and RF-coil sensitivity. Following the framework previously employed by our group for quantitative comparison of HP MRI detection sensitivity,^79^ the HP propane employed here contained two p-H_2_-derived hyperpolarized protons per molecule with an estimated polarization of ∼1% (at the time of production) and was administered essentially undiluted. In comparison, ^129^Xe polarization was ∼10%; however, natural-abundance xenon was employed (^129^Xe abundance ∼26.4%), and Xe constituted only ∼50% of the Xe/N_2_ mixture. Accounting for these factors and the ∼3.62-fold larger ^1^H gyromagnetic ratio gives an estimated ∼5.5-fold advantage in initial magnetization for HP propane (see Supporting Information for details).

RF detection further favors ^1^H under the conditions employed here. Using the classical frequency-dependent RF-sensitivity scaling of Hoult and Richards^8^ and Coffey and co-workers,^79^ after polarization is considered explicitly, the ∼3.62-fold difference in resonance frequency corresponds to an additional approximate sensitivity factor of 3.62^3/4^ ≈ 2.63. This yields an estimated ∼14-fold advantage for HP propane under otherwise equivalent RF-detection conditions. Moreover, the propane experiments employed a close-fitting proton knee coil with an estimated volume of ∼3 L, whereas ^129^Xe was detected using a ∼30 L clinical chest coil. Using an approximate geometric scaling of sensitivity with inverse characteristic coil dimension, (V_Xe/_V_H_)^1/3^, gives an additional factor of ∼2.15 and an overall idealized SNR advantage of ∼31-fold for HP propane. This coil correction is necessarily approximate because the actual sensitivity also depends on coil geometry, filling factor, Q, loading, matching, and receiver noise; a detailed derivation and assumptions are provided in the Supporting Information.

Experimentally, however, the initial-image SNR values were ∼28 for HP propane and ∼13 for HP ^129^Xe, corresponding to an observed advantage of only ∼2.2-fold (Figure 4). This difference is important because the idealized calculation assumes 1) that the nominal propane polarization is retained until imaging, 2) the same *T*_2_* of the two HP gases, and 3) that all of the HP propane spin order is NMR-visible. None of these three assumptions is expected to fully hold. The measured propane decay time in the excised lung was only *T*_d_ = 0.68 ± 0.12 s, compared with *T*_1_ = 10.9 ± 2.2 s for ^129^Xe; therefore, polarization losses during gas delivery, lung inflation, and the interval preceding acquisition disproportionately reduce the propane signal. Moreover, ^129^Xe *T*_2_ is expected to be substantially longer than that of propane. Furthermore, at 0.35 T the p-H_2_-derived propane protons approach the strong-coupling regime, and a fraction of the overpopulated singlet state order may reside in otherwise NMR-invisible states rather than directly observable magnetization. Consequently, the ∼31-fold value represents an idealized sensitivity estimate rather than a prediction of experimental SNR. The observed ∼2.2-fold advantage reflects the combined effects of high intrinsic ^1^H sensitivity, rapid propane polarization decay (likely the dominating factor), shorter *T*_2_*, incomplete NMR visibility of the HP spin order, and differences in RF hardware.

Given the overall semi-quantitative agreement of this sensitivity analysis with the experimental observations, it is also informative to consider HP propane and HP ^129^Xe under otherwise equivalent conditions. Assuming equal gas concentration (or pressure), identical coil geometry and comparable receiver performance, 1%-polarized propane provides two HP protons per molecule, whereas 10%-polarized natural-abundance xenon provides 0.264 × 0.10 = 0.0264 polarized ^129^Xe spins per xenon atom on a concentration-weighted basis. The corresponding polarized-spin ratio is therefore (2×0.01)/[0.264×0.10] ≈ 0.762. Accounting for the ∼3.62-fold greater ^1^H gyromagnetic ratio and the additional approximate ν^3/4^ RF-sensitivity dependence described above gives an estimated relative sensitivity of 0.762 × 3.62×3.62^3/4^ ≈ 7.2 in favor of HP propane. Thus, despite a tenfold lower nuclear polarization, ∼1%-polarized HP propane may provide approximately sevenfold greater instantaneous detection sensitivity than 10%-polarized natural-abundance HP ^129^Xe at the same gas concentration under otherwise comparable detection conditions. This comparison concerns instantaneous detection sensitivity and does not account for the substantially longer hyperpolarization lifetime of ^129^Xe, longer ^129^Xe *T*_2_* or incomplete NMR visibility of strongly coupled propane spin order.

The substantially shorter lifetime of HP propane compared with HP ^129^Xe nevertheless represents an important practical limitation. For *T*_d_ = 0.68 s, approximately 50% of the initial HP propane signal remains after ∼0.47 s, 37% after 0.68 s, and 20% after ∼1.1 s, emphasizing the need for rapid gas delivery, synchronization, and image acquisition. However, the present lung experiments also demonstrate that useful image SNR can be obtained within this short temporal window: the first HP propane lung image yielded an SNR of approximately 28, compared with approximately 13 for HP ^129^Xe under the experimental conditions employed here. As discussed above and in detail in the Supporting Information, this approximately 2.2-fold experimental SNR advantage is consistent with the intrinsically greater instantaneous detection sensitivity of HP ^1^H, although the magnitude of this advantage depends strongly on polarization, gas concentration, RF-coil performance, and the fraction of HP propane spin order that is NMR-visible. Thus, the principal potential advantage of HP propane is the feasibility of rapid, high-sensitivity proton-frequency ventilation imaging rather than a long observation window. Conversely, the much longer ^129^Xe lifetime provides a substantial advantage for protocols requiring prolonged or repeated acquisitions, broader spatial coverage, or additional temporal encoding. The presented results therefore demonstrate the feasibility of rapid HP propane lung imaging but do not establish superiority over HP ^129^Xe for pulmonary MRI; rather, the two agents provide different trade-offs between instantaneous detection sensitivity, hyperpolarization lifetime, and hardware requirements.

### Higher-Resolution Slice-Selective 2D MRI of HP Propane in Excised Rabbit Lungs

Given the relatively high SNR of the MRI scans with HP propane gas shown in Figure 4a, a separate feasibility scanning with a higher resolution (2×2 mm^2^ in-plane resolution) and a smaller slice thickness (10 mm) was performed as shown in Figure 5. The injection of HP propane gas reveals a uniform signal intensity throughout the pulmonary parenchymal architecture, as anticipated. Scan #2 was recorded immediately before the HP gas was injected, which was followed by Scan #3 recorded after the injection of HP propane in the lungs. Scan #4 revealed a substantially reduced signal intensity due to HP propane depolarization. The image obtained under thermal equilibrium conditions (Scan #5, not shown in Figure 5, see Supporting Information) was subtracted from Scan #3 and is presented as a difference image in Figure 5. The corresponding placement of the axial projection scan is shown as a schematic along with the photograph of the inflated lungs inside the protective plastic container.

**Figure 5:**
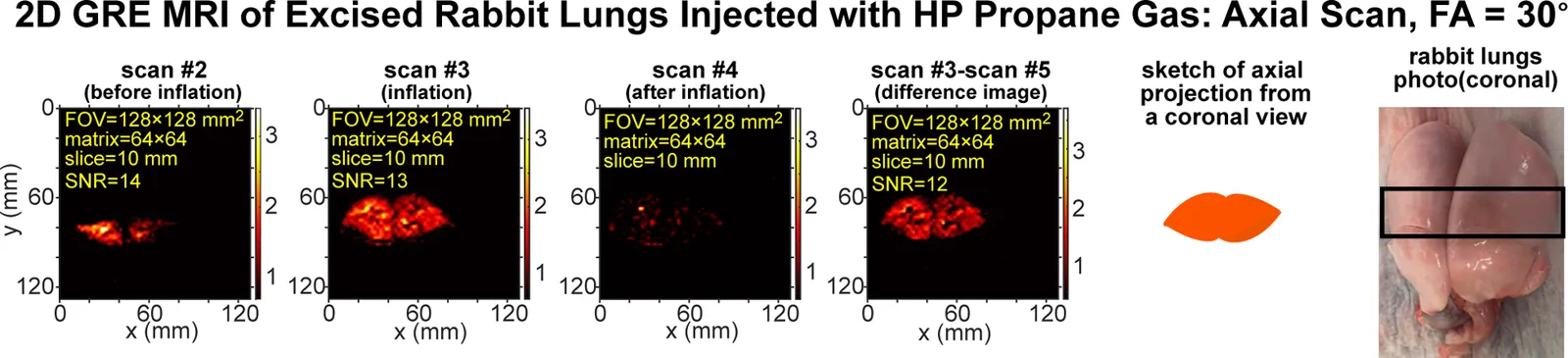
Representative high-resolution excised lungs imaging using HP propane gas at 0.35 T and the following parameters: TR = 17.19 ms, TE = 8.21 ms, total scan time of 16 repeat scans = 18 s, imaging matrix = 64 × 64, FOV = 128 × 128 mm^2^, FA = 30°, slice thickness = 10 mm. A series of lungs inflation images demonstrating the feasibility of a higher resolution ventilation lung imaging with HP propane gas: scan #2 (recorded immediately before the injection of HP propane gas and showing the collapsed lung); followed by scan # 3 (recorded immediately after the HP gas injection showing an inflated lungs illuminated with HP propane gas contrast); scan #4 (recorded immediately after scan #3 demonstrating gas depolarization); the difference image between scan #3 and scan #5; the sketch of the anticipated inflated lungs shape after gas inflation when scanned using axial projection; and (far right) a photo of the excised inflated rabbit lungs and an overlay showing the arrangement of axial slice localization (black box) in this coronal photograph.

## Conclusion

In this work we investigated the relaxation dynamics of HP propane gas, produced via heterogeneous pairwise *p*-H_2_ addition to propylene, across a broad range of magnetic field strengths (0.35 T to 7 T). The study demonstrates that the effective relaxation lifetime (characterized by the effective mono-exponential decay constant *T*_d_) of HP propane is strongly field-dependent, decreasing significantly from ∼1.5 s at 0.35 T to 0.25 s at 7 T in spherical plastic phantoms.

The extended hyperpolarization lifetime observed at 0.35 T is attributed to the presence of partially retained long-lived spin states (LLSS), facilitated by the strong coupling regime where chemical shift differences are similar to scalar spin–spin couplings. As the magnetic field increases, this protective condition is lost, and standard gas-phase relaxation mechanisms—primarily spin-rotation interactions and increased magnetic field inhomogeneities—drive the reduction in *T*_d_ values.

In physiologically relevant studies using excised rabbit lungs at 0.35 T, the *T*_d_ of HP propane was measured at 0.68 ± 0.12 s, a value shorter than that observed in hollow phantoms by approximately a factor of 2. This accelerated relaxation in the lungs’ environment is likely due to complex magnetic susceptibility mismatches at gas–tissue interfaces and increased surface-area-to-volume interactions with the pulmonary epithelium. While HP ^129^Xe in the lungs maintains a longer *T*_1_ (10.9 ± 2.2 s), HP propane offers a high-signal, proton-frequency alternative that is compatible with the standard, unmodified clinical MRI hardware. These findings highlight the potential of HP propane as a highly accessible contrast agent for rapid regional ventilation mapping of the lungs.

## Supplementary Material



## Data Availability

Raw spectroscopic (CSV format) and imaging (DICOM) data (ZIP), and examples of MATLAB processing codes and processed files are provided via Mendeley Data V1, DOI: 10.17632/g5fk82n7w7.1 .

## Notes

Additional experimental details, tabulated relaxation data, reproducibility data and plots, *T*_d_ relaxation studies in the gas phase, and image processing data and details (PDF), examples of *T*_d_ and image data processing using custom-made MATLAB codes (PDF)
