# Supplementary material for: Multi-Field Relaxation Dynamics of Parahydrogen-Hyperpolarized Propane Gas MRI Contrast Agent
Source: J Phys Chem A. 2026 Oct 8;130(43):8611–22. doi: 10.1021/acs.jpca.6c05094 (PMC13726385; doi:10.1021/acs.jpca.6c05094)
Supplement: Supplementary file 1 [file jp6c05094_si_001.pdf]

SUPPORTING INFORMATION FOR

Multi-Field Relaxation Dynamics of Parahydrogen-Hyperpolarized Propane Gas MRI Contrast Agent

Clementinah Oladun,[a] Nuwandi M. Ariyasingha,[a] Anna Samoilenko,[a] Md Raduanul H. Chowdhury,[a] Zhongjie Shi,[b] Kehuan Luo,[b] Sidhartha Tan,[b] Oleg G. Salnikov,[c] Dudari B. Burueva,[c] Alba Xhupi,[a] Majd Suleiman,[a] Jerome McCreadie,[a] Larisa M. Kovtunova,[c] Igor V. Koptyug,[c] Juri G. Gelovani,[a,d] Panayiotis Nikolaou,[e] Jessica Ettedgui,[f] Rolf E. Swenson,[f] Kazutoshi Yamamoto,[g] Norikazu Koyasu,[g] Murali C. Krishna,[g] Joseph W. Plummer,[h] Adrienne Campbell-Washburn,[h] Michael J. Barlow,[i] Boyd M. Goodson,[j] Eduard Y. Chekmenev[a]*

[a] Department of Chemistry, Integrative Biosciences (IBio), Karmanos Cancer Institute (KCI), Wayne State University, Detroit, Michigan 48202, United States

[b] Department of Pediatrics, Wayne State University, Detroit, Michigan 48202, United States

[c] International Tomography Center SB RAS, 3A Institutskaya st., Novosibirsk 630090, Russia

[d] College of Medicine & Health Sciences, United Arab Emirates University, Al Ain, United Arab Emirates

[e] XeUS Technologies Ltd., Nicosia 2312, Cyprus

[f] Chemistry and Synthesis Center, National Heart, Lung, and Blood Institute, 9800 Medical Centre Drive, Rockville, Bethesda, Maryland, 20850, United States

[g] Center for Cancer Research, NCI, Building 10 B3B69, Bethesda, MD 20892, United States

[h] Cardiovascular Branch, Division of Intramural Research, National Heart, Lung, and Blood Institute, National Institutes of Health, Bethesda, MD 20892, United States

[i] Sir Peter Mansfield Imaging Centre, University of Nottingham, Nottingham NG72RD, United Kingdom

[j] School of Chemical and Biomolecular Sciences, Southern Illinois University, Carbondale, Illinois 62901, United States

Table of Contents

1. REPRODUCIBILITY DATA FOR T_1 VALUES OF HP PROPANE AT 1.4 T S3

2. HOLLOW PHANTOM SPHERE RELAXATION IMAGING IN 0.35 T MRI SCANNER S3

3. HOLLOW PHANTOM SPHERE RELAXATION IMAGING IN 0.55 T MRI SCANNER S6

4. HOLLOW PHANTOM SPHERE RELAXATION IMAGING IN 3 T MRI SCANNER S8

5. HOLLOW PHANTOM SPHERE RELAXATION IMAGING IN 7 T MRI SCANNER S10

6. PHANTOM RELAXATION STUDIES AT LOW FLIP ANGLES (1°, 2°, 3° AND 5°) ON FOUR MRI SCANNERS (0.35 T, 0.55 T, 3 T AND 7 T) S13

7. T_1 RELAXATION STUDIES OF THERMALLY POLARIZED PROPANE GAS AT 1.4 T, 7.05 T, 9.4 T, AND 16.4 T S14

8. COMPARATIVE RELAXATION STUDIES AT 0.35 T MRI USING HP PROPANE AND HP ^{129}XE GAS IN EXCISED RABBIT LUNGS S15

9. HIGHER RESOLUTION MRI SCANNING OF EXCISED RABBIT LUNGS WITH HP PROPANE GAS AT 0.35 T S22

10. MATLAB CODE FOR GAS-PHASE T_1 RELAXATION DYNAMICS STUDIES S23

11. MATLAB POST PROCESSING IMAGING ANALYSIS PROTOCOL S28

12. MATLAB CODE FOR DIFFERENCE IMAGE PROCESSING OF 2D GRE MRI DATA OBTAINED WITH HP PROPANE GAS IN PHANTOM AND EXCISED RABBIT LUNGS AT 0.35 T MRI SCANNER S28

I) FILE STORAGE FORMAT AND ARCHITECTURE S28

13. MATLAB CODE FOR GAS-PHASE T_D RELAXATION DYNAMICS STUDIES WITH HP PROPANE GAS IN EXCISED RABBIT LUNGS AND PHANTOMS AT 0.35 T........ S31

14. MATLAB CODE FOR RAW IMAGING DATA WITH HP ^{129}XE GAS IN EXCISED RABBIT LUNGS AT 0.35 T 33

15. MATLAB CODE FOR GAS-PHASE T_D RELAXATION DYNAMICS STUDIES WITH HP ^{129}XE GAS IN EXCISED RABBIT LUNGS AT 0.35 T S34

16. MATLAB CODE FOR GAS-PHASE T_D RELAXATION DYNAMICS IN PHANTOM AT 0.55 T MRI SCANNER S37

I) FILE STORAGE FORMAT AND ARCHITECTURE S37

17. MATLAB CODE FOR GAS-PHASE T_D RELAXATION DYNAMICS WITH HP PROPANE IN PHANTOM AT 3 T MRI SCANNER S39

I) FILE STORAGE FORMAT AND ARCHITECTURE S39

18. MATLAB CODE FOR GAS-PHASE T_D RELAXATION DYNAMICS OBTAINED IN PHANTOM WITH HP PROPANE IN 7 T MRI SCANNER........ S41

I) FILE STORAGE FORMAT AND ARCHITECTURE S41

19. DATA AVAILABILITY STATEMENT S44

20. DETAILED SENSITIVITY COMPARISON OF HP PROPANE AND HP ^{129}XE........ S44

21. REFERENCES USED IN SUPPORTING INFORMATION (SI) S47

1. Reproducibility Data for T_1 values of HP propane at 1.4 T

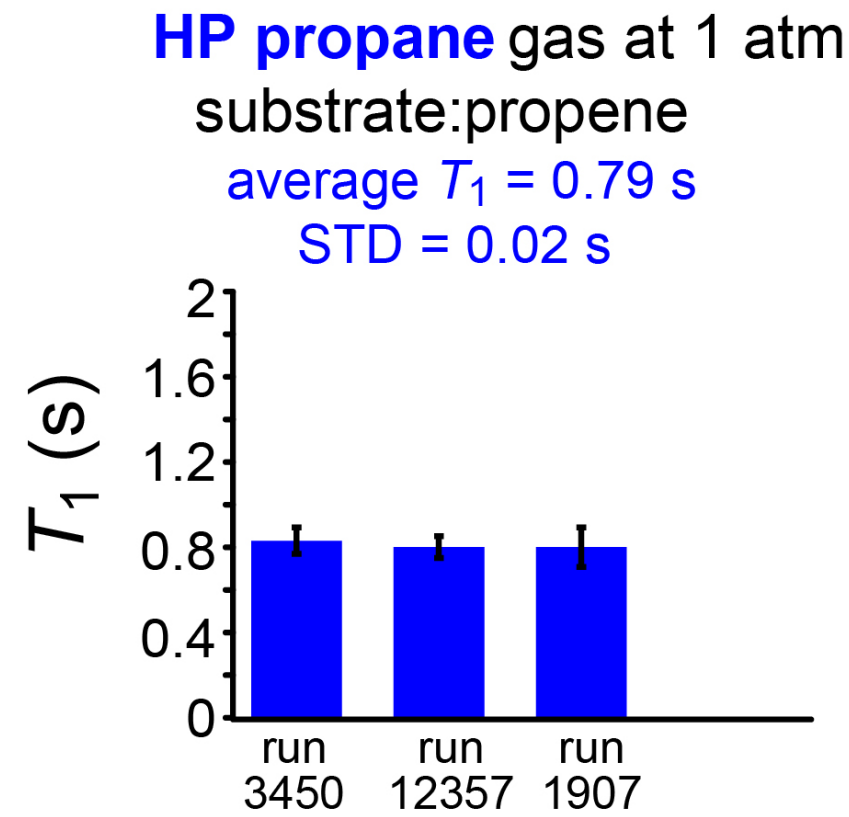

Figure S1. Reproducibility plot of shot-to-shot T_1 data collected for HP propane gas at atmospheric (1 atm) pressure.

T_1 H_A (s)	T_1 H_A error bar (s)	T_1 H_B (s)	T_1 H_B error bar (s)
0.81 (run 3450)	0.06	0.73	0.06
0.78 (run 12357)	0.05	0.79	0.03
0.78 (run 1907)	0.09	0.76	0.11

Table S1. T_1 values and the corresponding error bars of H_A and H_B sites of HP propane gas at 1 atm (see **Figure 1** for H_A and H_B notation details). The raw data for the entries in this table is supplied in the non-deaccumulated (raw) format in the package specified in the data availability statement. The data structure is as follows: run number (*i.e.*, **1350**) with subfolders of "HP_data" and "thermal_reference_data". All propane data in this table comes from Ref. # [1].

2. Hollow Phantom Sphere Relaxation Imaging in 0.35 T MRI scanner

In this section, we present the whole series of the imaging for the relaxation of HP propane gas in hollow phantom sphere in 0.35 T which are presented in **Figure 3a** with a 4×4 mm^2 pixel size (**Figure S2**). The DICOM images were analyzed using Matlab. Background subtraction was done by subtracting the 14th image in the series from images #1 through #16. Image #5 was taken as the first data point for the plotting of the relaxation data as we can clearly ascertain that there was no more gas flow into this phantom at this point. Images #5 to #14 were used in the relaxation plot (shown as ROI analysis in **Figure 3a)**. During analysis, the signal intensities of the images of interest were computed (**Table S2**) and used in the plot of the ROI analysis using Origin.

Time (s)	Signal intensity (a.u)
0	3080.813
1.12	1633.875
2.24	729.75
3.36	244.125
4.48	97.1875
5.60	34.375
6.72	-15.375
7.84	10.1875
8.96	-28.8125
10.08	0

Table S2. Signal intensities of scan #5 to scan #14 of hollow phantom sphere at 0.35 T used in the ROI analysis shown in **Figure 3a.**

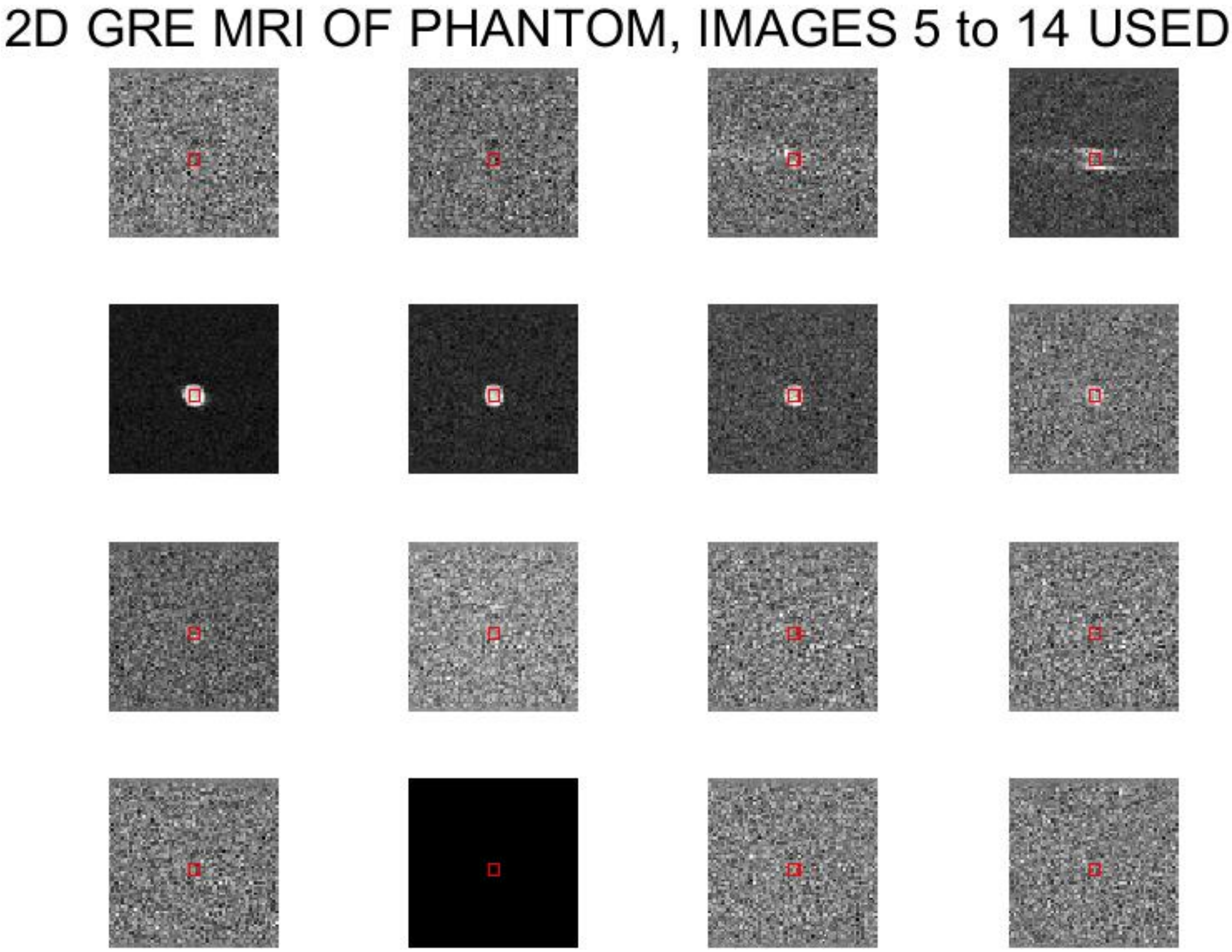

Figure S2. Time series of slice-selective 2D GRE images of HP propane gas injected into hollow phantom sphere, acquired by utilizing a 0.35 T MRI scanner and a knee RF coil. Axial-projection scans of HP propane gas signal decay in the hollow phantom sphere recorded using FA = 5° at 1.12 s temporal resolution. The presented images were obtained by subtracting image #14 from each image in the series. The imaging parameters employed were: 64×64 imaging matrix and 256×256 mm^2 field of view (FOV), 50 mm slice thickness, TR = 17.19 ms, and TE = 8.21 ms. The red box highlights the 4×4 pixel matrix with the average intensity region which serves as our region of interest (ROI) shown in **Figure 3a**.

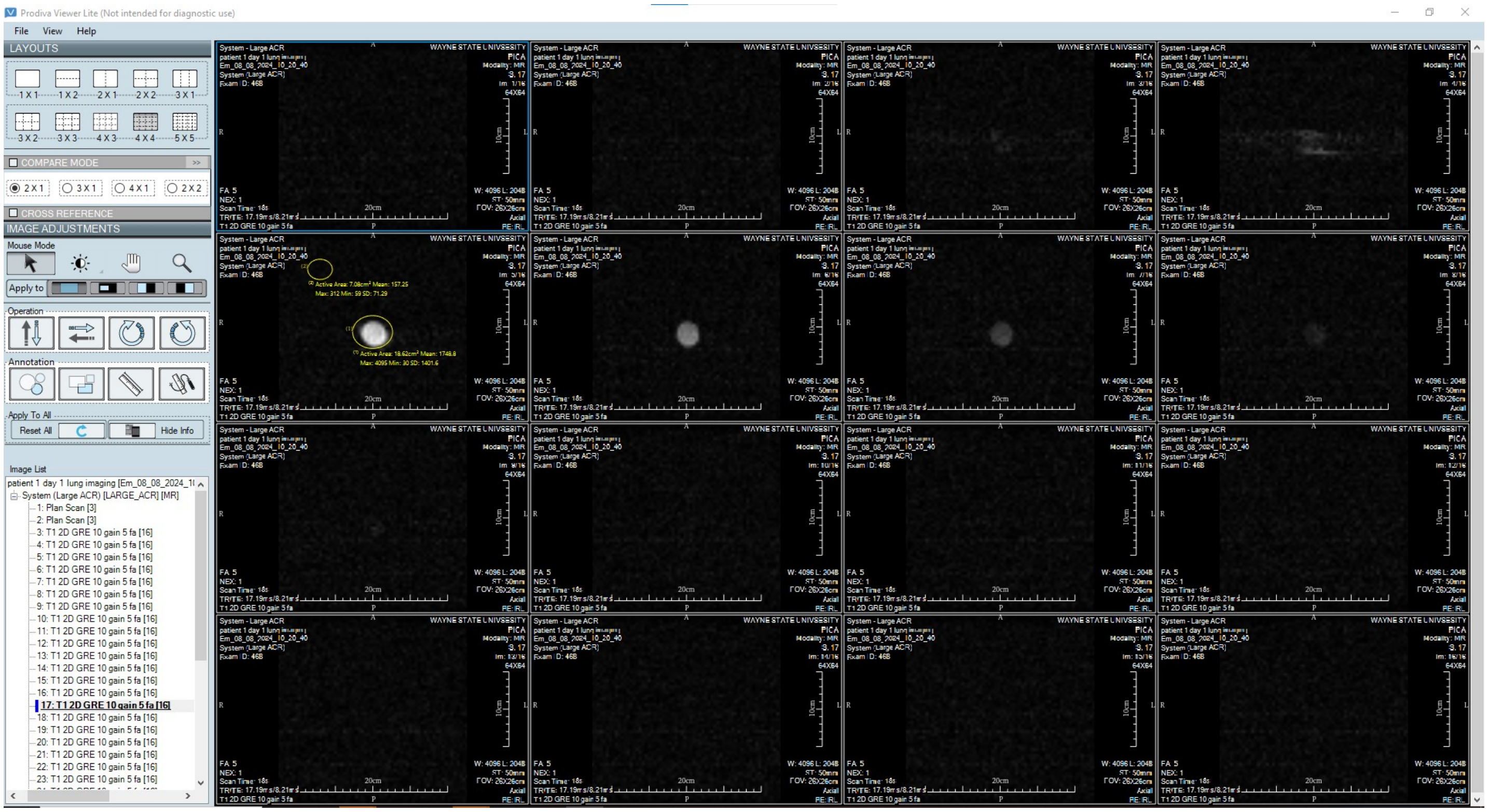

Figure S3. Screenshot of images processed by the MRI scanner software during the experiment presented in **Figure 3a** and **Figure S2** (axial projection, 4×4 mm^2 pixel size) without any data post-processing.

3. Hollow Phantom Sphere Relaxation Imaging in 0.55 T MRI scanner

In this section, we present the whole series of the imaging for the relaxation of HP propane gas in hollow phantom sphere in 0.55 T which are presented in **Figure 3b** with a 4.6×4.6 mm^2 pixel size (**Figure S4**).

For low-field assessment, dynamic gas imaging was carried out on a 0.55 T clinical spectrometer system (MAGNETOM Free.Max, Siemens Healthineers, Erlangen, Germany) operating at a ^1H resonance frequency of 23.39 MHz. A BEAT Interactive pulse sequence (operating as a fast gradient-echo framework) was employed to map the HP gas cell dynamics using a single 8 mm slice thickness. Spatial encoding parameters were configured natively as a square 64×64 matrix over a 32.0×32.0 cm FOV. For separate tracking experiments, the excitation pulse flip angle was varied across values of 3° and 5° to systematically manipulate longitudinal magnetization consumption.

The temporal framework consisted of 499 consecutive dynamic frames captured continuously over a total experimental window of 147 seconds. The hardware timing was configured with a TR of 4.57 ms and aTE of 2.19 ms. This hardware configuration resulted in a precise temporal resolution (frame period, Delta t) of 292.6 ms per image frame (approximated as 293 ms in post-processing). The data loop was natively reconstructed on-scanner without asymmetric k-space padding or truncation to output a standard 64×64 spatial pixel matrix.

Image Acquisition / 0.55 T Experimental Parameters

- **Spectrometer System:** Siemens MAGNETOM Free.Max (0.55 T)
- **Pulse Sequence Type:** BEAT Interactive (Fast Gradient Echo)
- **Sample / Phantom Geometry:** Hyperpolarized (HP) propane gas phantom
- **Resonance Frequency (^1H):** 23.39 MHz
- **Repetition Time (TR):** 4.57 ms
- **Echo Time (TE):** 2.19 ms

- **Flip Angle (Pulse Angle):** 3° and 5° (for separate experiments)
- **Slice Thickness:** 8 mm (Single Slice)
- **Field of View (FOV):** 32.0×32.0 cm
- **Native Acquisition Matrix:** 64×64
- **Reconstructed Image Matrix:** 64×64 pixels
- **Temporal Framework:** 499 consecutive time-course frames
- **Frame Period (Δt):** 293 ms per frame
- **Total Scan Duration:** 147.0 seconds

The DICOM images were analyzed using MATLAB. Background subtraction was done by subtracting the 8th image in the series from images #1 through #8. Image #1 was taken as the first data point for the plotting of the relaxation. Images #1 to #8 were used in relaxation plot (shown as ROI analysis in **Figure 3b)**. During analysis, the signal intensities of the images of interest were computed (**Table S3**) and used in the plot of the ROI analysis with Origin.

Time (s)	Signal intensity (a.u)
0	26.68
0.293	17.16
0.586	7.60
0.879	6.12
1.172	4.64
1.465	4.36
1.758	3.08
2.051	0

Table S3. Signal intensities of scan #1 to scan #8 of hollow phantom sphere at 0.55 T used in the ROI analysis shown in **Figure 3b.**

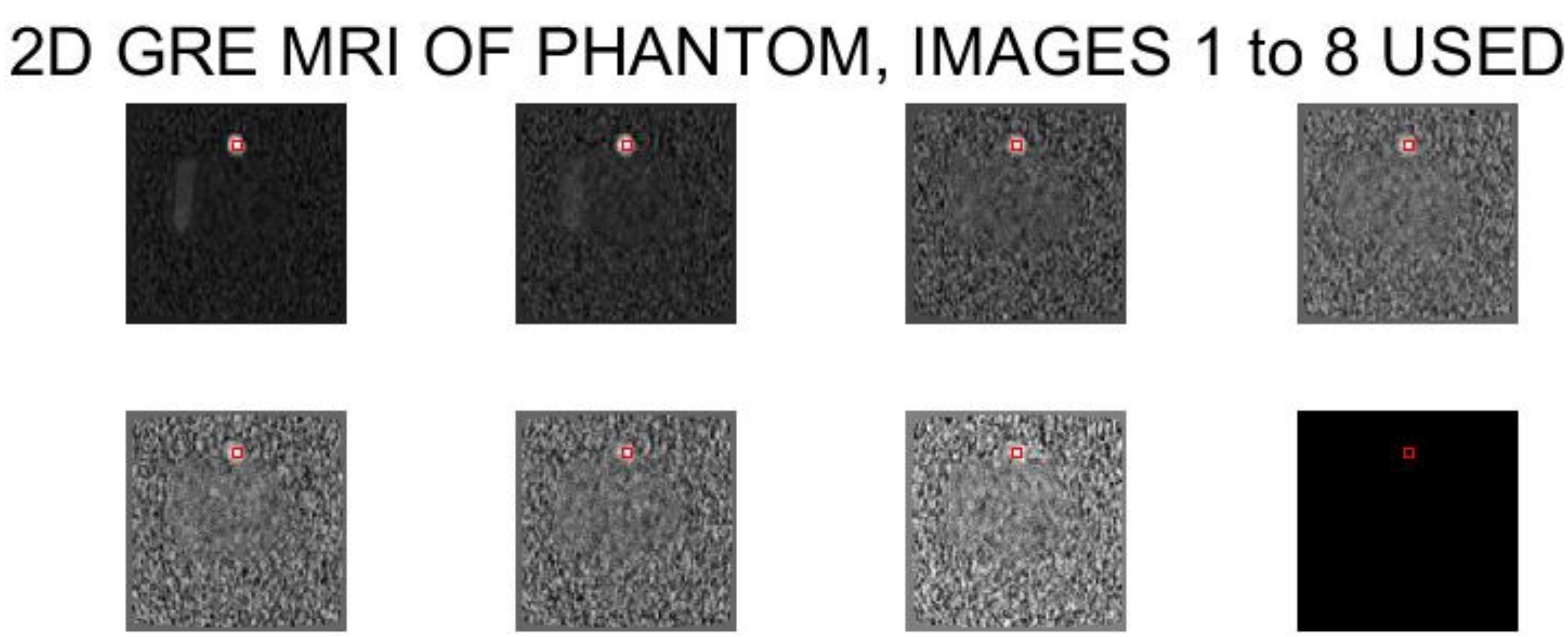

Figure S4. Time series of slice-selective 2D GRE images of HP propane gas injected into hollow phantom sphere, acquired by utilizing a 0.55 T MRI scanner and a chest RF coil. Axial-projection scans of HP propane gas signal decay in the hollow phantom sphere recorded using FA = 5° at 0.293 s temporal resolution. The presented images were obtained by subtracting image #8 from each image in the series. The imaging parameters employed were as follows: 64×64 imaging matrix and 320×320 mm^2 FOV, 8 mm slice thickness, TR = 4.57 ms and TE = 2.19 ms. The red box highlights the 5×5 pixel matrix with the average intensity region which serves as our region of interest (ROI) for 0.55 T in **Figure 3b** which used images #1 to #8.

4. Hollow Phantom Sphere Relaxation Imaging in 3 T MRI scanner

In this section, we present the whole series of the imaging for the relaxation of HP propane gas in hollow phantom sphere in 3 T which are presented in **Figure 3b** with a 1×1 mm^2 pixel size (**Figure S5**).

Image Acquisition / 3 T Experimental Parameters

Dynamic NMR imaging was performed to track the real-time transport and signal dynamics of an HP propane gas sample loaded into a hollow plastic sphere phantom. The dataset was acquired on a 3 T spectrometer (ParaVision 360 V2.0, Bruker BioSpin MRI GmbH) operating at a ^1H resonance frequency of 127.6227 MHz and utilizing a dedicated RF resonator coil. Imaging was conducted in a standard 2D orientation using a fast low-angle shot (FLASH) gradient-echo pulse sequence. To optimize the SNR for the low-density gas within the spherical volume, a single, thick 15 mm slice was excited across a 6.4 × 6.4 cm^2 FOV. For separate tracking experiments, the excitation pulse angle was varied across values of 1°, 2°, 3°, and 5° to systematically manipulate longitudinal magnetization consumption.

To continuously monitor the gas dynamics, a total of 64 sequential temporal frames were recorded during a single continuous scan. Each frame was natively acquired using an asymmetric k-space grid consisting of 1024 readout points and 40 phase-encoding steps. By operating the gradient hardware at its maximum performance limits, the sequence achieved an ultra-short TE of 1.60 ms and a TR of 3.69 ms. This configuration yielded a high temporal resolution of exactly 147.44 ms per image frame. Following acquisition, the raw 40-step phase data was automatically zero-padded in k-space by the reconstruction engine to output a symmetric viewing matrix of 64 × 64 pixels per frame.

Experimental Parameters

The primary imaging and spectrometer parameters utilized during the 3 T hyperpolarized propane gas experiment are summarized below:

- **Spectrometer System:** 3T Bruker ParaVision 360 (V2.0 pl 1)
- **Pulse Sequence:** 2D Gradient Echo (FLASH.ppg)
- **Sample/Phantom:** HP propane gas in a hollow plastic sphere phantom
- **Resonance Frequency (^{1}H):** 127.6227 MHz
- **Repetition Time (TR):** 3.69 ms
- **Echo Time (TE):** 1.60 ms
- **Flip Angle (Pulse Angle):** 1°, 2°, 3°, and 5° (for separate experiments)
- **Slice Thickness:** 15 mm (Single Slice)
- **Field of View (FOV):** 6.4 × 6.4 cm^2
- **Native Acquisition Matrix:** 1024 readout points x 40 phase steps
- **Reconstructed Image Matrix:** 64 × 64 pixels (via zero-padding)
- **Temporal Framework:** 64 consecutive time-course frames
- **Frame Period (Δt):** 147.44 ms per frame
- **Total Scan Duration:** 9.44 seconds

The DICOM images were analyzed using MATLAB. Background subtraction was done by subtracting the 64th image in the series from images #1 through #64. Image #26 was taken as the first data point for the plotting of the relaxation data. Images #26 to #64 were used in relaxation plot (shown as ROI analysis in **Figure 3b)**. During analysis, the signal intensities of the images of interest were computed (**Table S4**) and used in the plot of the ROI analysis with Origin.

Time (s)	Signal intensity (a.u)
0	19363.510
0.147	18159.225
0.294	14364.838
0.441	10231.265
0.588	7070.367
0.735	4736.816
0.882	3495.837
1.029	2652.408
1.176	2002.816
1.323	1589.000
1.470	920.592
1.617	500.204
1.764	250.929
1.911	80.327
2.058	158.387
2.205	-34.061
2.352	98.143
2.499	-156.265
2.646	-169.673
2.793	-136.816
2.940	137.489
3.087	-320.857
3.234	18.918
3.381	-165.020
3.528	65.327
3.675	66.898
3.822	-169.265
3.969	-158.102
4.116	-109.429
4.263	-185.918
4.410	-187.633
4.557	-60.285
4.704	-13.776
4.851	3.469
4.998	-169.571
5.145	-117.204
5.292	-77.776
5.439	-46.836
5.586	0

Table S4. Signal intensities of scan #26 to scan #46 of hollow phantom sphere at 3 T used in the ROI analysis shown in **Figure 3b.**

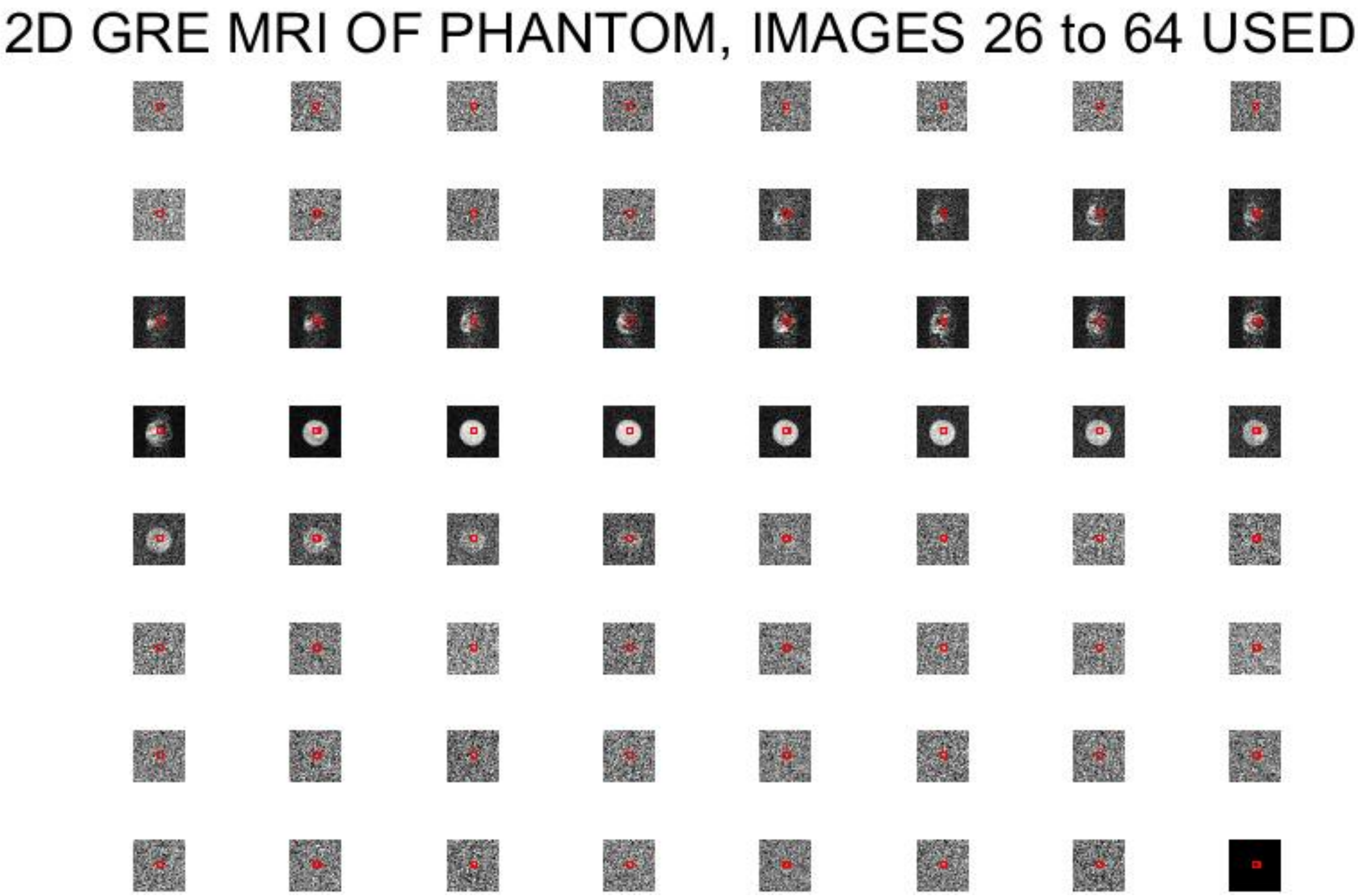

Figure S5. Time series of slice-selective 2D GRE images of HP propane gas injected into hollow phantom sphere, acquired by utilizing a 3 T MRI scanner and a volume RF coil. Axial-projection scans of HP propane gas signal decay in the hollow phantom sphere recorded using FA = 5° at 0.147 s temporal resolution. The presented images were obtained by subtracting image #64 from each image in the series. The imaging parameters employed were as follows: 64×64 imaging matrix and 64×64 mm^2 field of view (FOV), 15 mm slice thickness, TR = 3.69 ms, and TE = 1.59 ms. The red box highlights the 7×7 pixel matrix with the average intensity region which serves as our region of interest (ROI) for 3 T shown in **Figure 3b** which used images #26 to #64.

5. Hollow Phantom Sphere Relaxation Imaging in 7 T MRI scanner

In this section, we present the whole series of the imaging for the relaxation of HP propane gas in hollow phantom sphere in 7 T which are presented in **Figure 3b** with a 3×3 mm^2 pixel size (**Figure S6**).

Image Acquisition

For high-field assessment, dynamic gas imaging was carried out on a 7 T spectrometer system (ParaVision 360 V2.0, Bruker BioSpin MRI GmbH) operating at a ^1H resonance frequency of 300.2905 MHz and using a dedicated RF resonator coil. A 2D FLASH gradient-echo pulse sequence was employed to map the hyperpolarized gas cell dynamics across a single 10 mm slice thickness. Spatial encoding parameters defined a symmetric 32×32 pixel target reconstruction grid over a 9.6×9.6 cm field of view (FOV). For this dynamic tracking experiment, the excitation pulse angle was configured at 5°.

The temporal framework consisted of 32 consecutive dynamic frames captured continuously over a total experimental window of 9.25 seconds. Each frame was natively recorded as an asymmetric grid of 1024 readout points and 32 true phase-encoding steps. The hardware timing was configured with a TR of 9.04 ms and aTE of 2.75 ms. This hardware loop resulted in a precise temporal resolution (frame period, Δt) of 289.28 ms per image

frame. The resulting k-space array was symmetric-truncated along the readout axis by the ParaVision reconstruction pipeline to output a clean, non-interpolated 32×32 spatial pixel matrix.

7 T Experimental Parameters

- **Spectrometer System:** Bruker ParaVision 360 (V2.0 pl 1)
- **Pulse Sequence Type:** 2D Gradient Echo (FLASH.ppg)
- **Sample / Phantom Geometry:** Hyperpolarized (HP) propane gas phantom
- **Resonance Frequency (1H):** 300.2905 MHz
- **Repetition Time (TR):** 9.04 ms
- **Echo Time (TE):** 2.75 ms
- **Flip Angle (Pulse Angle):** 5°
- **Slice Thickness:** 10 mm (Single Slice)
- **Field of View (FOV):** 9.6×9.6 cm^2
- **Native Acquisition Matrix:** 1024 readout points × 32 phase steps
- **Reconstructed Image Matrix:** 32×32 pixels (via truncation)
- **Temporal Framework:** 32 consecutive time-course frames
- **Frame Period (Δ t):** 289.28 ms per frame
- **Total Scan Duration:** 9.25 seconds

The DICOM images were analyzed using MATLAB. Background subtraction was done by subtracting the 20th image in the series from images #1 through #32. Image #11 was taken as the first data point for the plotting of the relaxation curve. Images #11 to #20 were used in relaxation plot (shown as ROI analysis in **Figure 3b)**. During analysis, the signal intensities of the images of interest were computed (**Table S5**) and used in the plot of the ROI analysis with Origin.

Time (s)	Signal intensity (a.u)
0	3080.813
0.289	1452.540
0.578	450.030
0.867	107.760
1.156	1.800
1.445	54.400
1.734	-39.200
2.023	-2.640
2.312	15.400
2.601	0.00

Table S5. Signal intensities of scan #11 to scan #20 of hollow phantom sphere at 7 T used in the ROI analysis shown in **Figure 3b.**

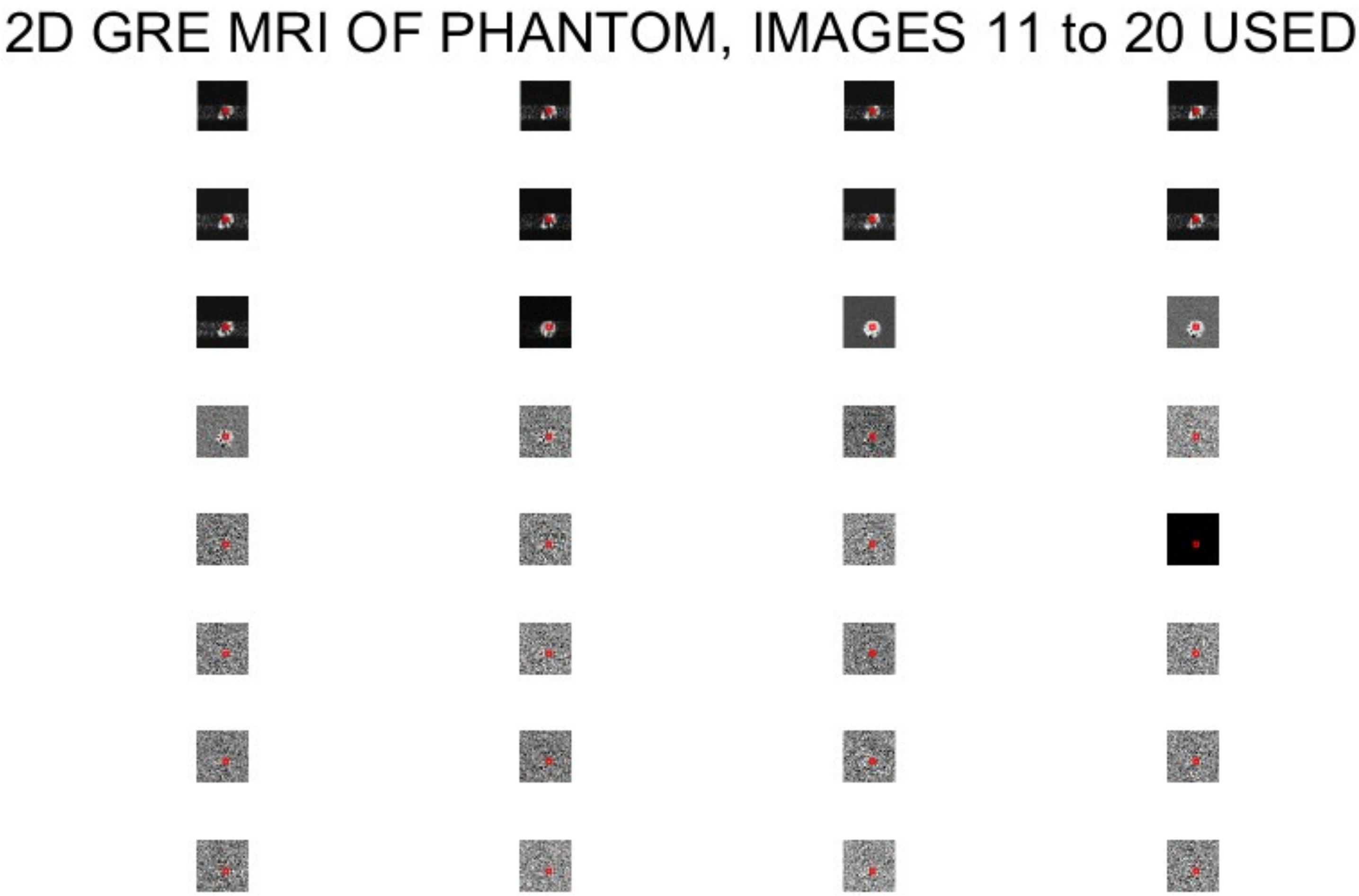

Figure S6. Time series of slice-selective 2D GRE images of HP propane gas injected into hollow phantom sphere, acquired by utilizing a 7 T MRI scanner and a volume RF coil. Axial-projection scans of HP propane gas signal decay in the hollow phantom sphere recorded using FA = 5° at 0.289 s temporal resolution. The presented images were obtained by subtracting image #20 from each image in the series. The imaging parameters employed were as follows: 32×32 imaging matrix (interpolated to 64×64) and 96×96 mm^2 field of view (FOV), 10 mm slice thickness, TR = 9.04 ms, and TE = 2.75 ms. The red box highlights the 5×5 pixel matrix with the average intensity region which serves as our region of interest (ROI) for 7 T shown in **Figure 3b** using images #11 to scan #20.

6. Phantom relaxation studies at low flip angles (1°, 2°, 3° and 5°) on four MRI scanners (0.35 T, 0.55 T, 3 T and 7 T)

The data presented in **Table S6** below represents multiple data acquired at 0.35 T, 0.55 T, 3 T and 7 T respectively to study the effect of the flip angle used during imaging acquisition on the relaxation of the HP propane gas at these different fields. Images were acquired as described above for each MRI scanner at different flip angles which are 1°, 2°, 3° and 5°. Where possible, multiple data were acquired at the same flip angle and each individual data was analyzed as described above for each MRI scanner. Signal intensities value were acquired from images of interest during MATLAB processing of the DICOM files and these intensities were later plotted agaimst time in Origin to get a relaxation decay constant as well as error bar values. Where we have two or more data available for a specific flip angle in an MRI scanner, the average value and standard deviation of the relaxation constants was calculated, and this was used in the plotting of the results presented in **Figure 3c**. However, for any flip angle with just one data point, the relaxation constant and error bar were used.

0.35 T			0.55 T		3 T		7 T	
**FA (°)**	T_d **(s)**	**error bar (s)**	T_d **(s)**	**error bar (s)**	T_d **(s)**	**error bar (s)**	T_d **(s)**	**error bar (s)**
1	1.41 (folder S12)	0.06	-	-	0.52 (folder 12)	0.03	0.20 (folder 15)	0.01
1	1.55 (folder S13)	0.06	-	-	0.61 (folder 13)	0.04	-	-
1	1.28 (folder S14)	0.04	-	-	-	-	-	-
2	-		-	-	0.48 (folder 14)	0.02	0.23 (folder 14)	0.01
3	1.47 (folder S10)	0.05	0.79 (folder 12)	0.06	0.51 (folder 15)	0.01	0.26 (folder 13)	0.01
3	1.46 (folder S16)	0.03	0.51 (folder 13)	0.05	-	-	-	-
5	1.48 (folder S9)	0.08	0.61 (folder 4)	0.06	0.54 (folder 16)	0.02	0.22 (folder 11)	0.003
5	1.54 (folder S17)	0.07	0.58 (folder 18)	0.04	-	-	0.24 (folder 12)	0.003
5	-	-	0.58 (folder 19)	0.05	-	-	-	-

Table S6. Relaxation constant T_d of HP propane gas in hollow phantom sphere at different flip angle and various MRI scanners. The folder numbers in parentheses refer to the location of the raw data in the shared data files.

7. T_1 relaxation studies of thermally polarized propane gas at 1.4 T, 7.05 T, 9.4 T, and 16.4 T

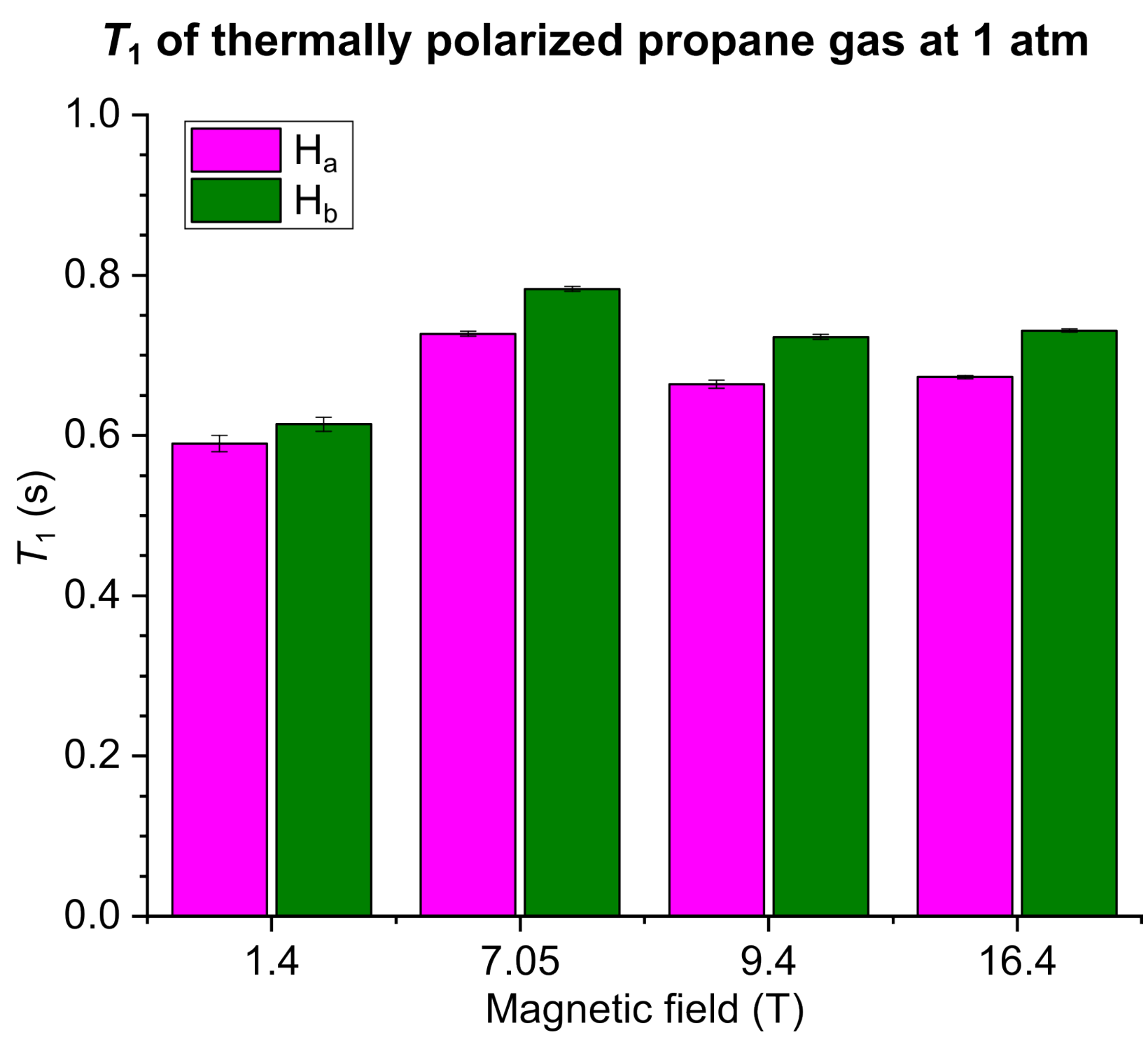

Figure S7. T_1 relaxation times of thermally polarized propane gas at 1 atm pressure and various external magnetic fields (1.4 T, 7.05 T, 9.4 T, and 16.4 T) measured using the inversion-recovery RF pulse sequence. The corresponding tabulated data is presented in **Table S7**.

Magnetic field strength (T)	T_1 H_A (s)	T_1 H_A error bar (s)	T_1 H_B (s)	T_1 H_B error bar (s)
1.4	0.59	0.01	0.614	0.009
7.05	0.727	0.003	0.783	0.003
9.4	0.664	0.005	0.723	0.003
16.4	0.673	0.002	0.731	0.002

Table S7. T_1 values and the corresponding error bars for H_A and H_B sites of thermally polarized propane gas at 1 atm pressure and various external magnetic fields (1.4 T, 7.05 T, 9.4 T, and 16.4 T) (see **Figure 1** for H_A and H_B notation details).

8. Comparative relaxation studies at 0.35 T MRI using HP propane and HP ^{129}Xe gas in excised rabbit lungs

In this section, we present the whole series of the imaging for the relaxation of HP propane gas in excised rabbit lungs in 0.35 T which are presented in **Figure 4a** with a 4×4 mm^2 pixel size (**Figure S8**). The DICOM images were analyzed using MATLAB. Background subtraction was done by subtracting the 10th image in the series from images #1 through #16. Image #4 was taken as the first data point for the plotting of the relaxation data as we can clearly ascertain that there was no more gas flow into the excised rabbit lungs and the lungs are fully inflated at this point. Images #4 to #10 were used in relaxation plot (shown as ROI analysis in **Figure 4a)**. During analysis, the signal intensities of the images were computed and those of #4 to #10 (**Table S8**) were used in the plot of the ROI analysis using Origin.

Time (s)	Signal intensity (a.u)
0	1479.64
1.18	238.68
2.36	68.84
3.54	-3.60
4.72	-1.44
5.90	-21.92
7.08	0

Table S8. Signal intensities of scan #4 to scan #10 of excised rabbit lungs at 0.35 T used in the ROI analysis shown in **Figure 4a.**

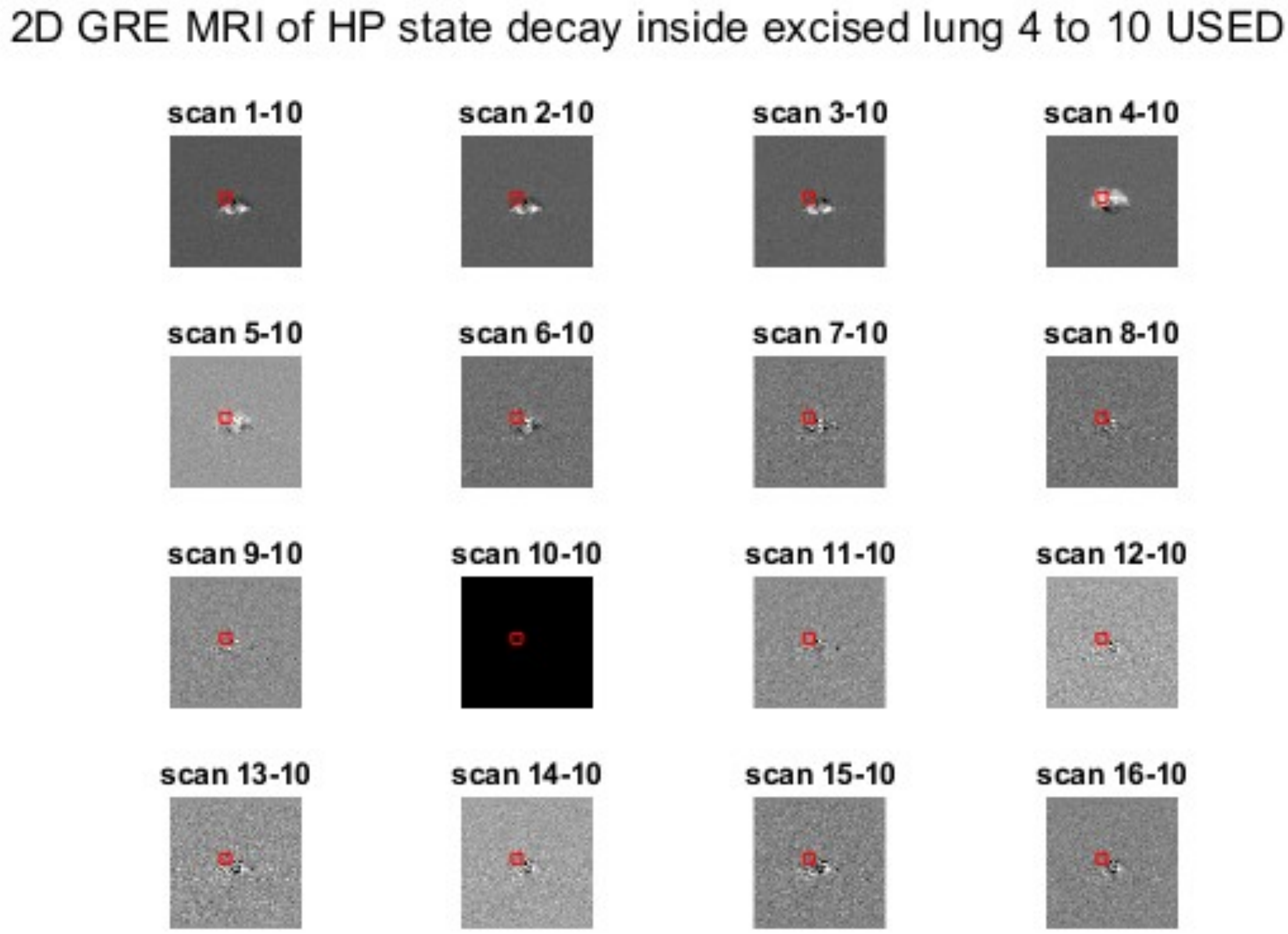

Figure S8. Time series of slice-selective 2D GRE images of HP propane gas injected into excised rabbit lungs, acquired by utilizing a 0.35 T MRI scanner and a knee RF coil. Axial-projection scans of HP propane gas signal decay in excised rabbit lungs recorded using FA = 5° at 1.18 s temporal resolution. The presented images were obtained by subtracting image #10 from each image in the series. The imaging parameters employed were as follows: 64×64 imaging matrix and 256×256 mm^2 field of view (FOV), 50 mm slice thickness, TE = 18.73 ms, and TR = 8.21 ms. The red box highlights the 5×5 pixel matrix with the average intensity region which serves as our region of interest (ROI) shown in **Figure 4a**.

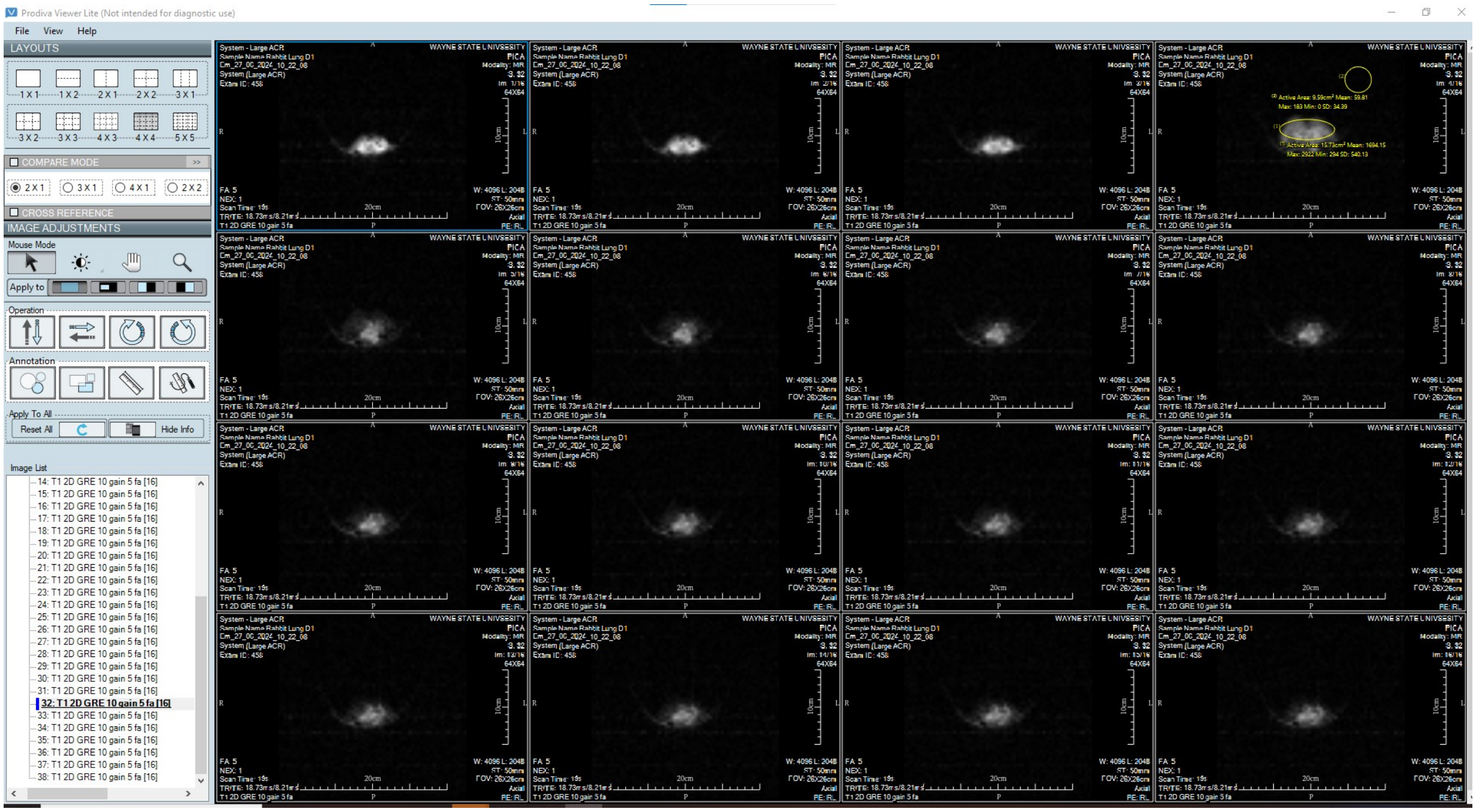

Figure S9. Screenshot of images processed by the MRI scanner software during the experiment presented in **Figure 4a** (axial projection, 4×4 mm^2 pixel size) without any data post-processing.

The data for the reproducibility plot for HP propane gas in excised rabbit lungs at different flip angles in 0.35 T clinical MRI scanner is presented below in **Table S9.** Signal intensities values were acquired from images of interest during MATLAB processing of the DICOM files and these intensities were later plotted agaimst time in Origin to get a relaxation decay constant as well as error bar values. Thereafter, the average value and standard deviation of the relaxation time constants at each flip angle was calculated and this average value is what is presented in the histogram in **Figure 4a.**

FA 5°		FA 3°		FA 2°		FA 1°	
T_d (s)	error bar (s)	T_d (s)	error bar (s)	T_d (s)	error bar (s)	T_d (s)	error bar (s)
0.59 (folder S16)	0.03	0.72 (folder S23)	0.02	0.60 (folder S28)	0.02	0.67. (folder S29)	0.03
0.48 (folder S18)	0.07	0.66 (folder S24)	0.05	0.77 (folder S38)	0.008	0.72 (folder S39)	0.03
0.79 (folder S27)	0.07	0.79 (folder S26)	0.05	0.54 (folder S12)	0.01	0.76 (folder S14)	0.02
0.56 (folder S28)	0.04	0.96 (folder S30)	0.05	0.64 (folder S13)	0.02	0.62 (folder S15)	0.02
0.85 (folder S29)	0.04	0.77 (folder S33)	0.03	0.70 (folder S16)	0.02	0.81 (folder S30)	0.04
0.66 (folder S32)	0.02	0.81 (folder S27)	0.02	0.59 (folder S29)	0.02	0.82 (folder S31)	0.02
0.66 (folder S26)	0.01	0.57 (folder S37)	0.10	0.67 (folder S32)	0.006	0.90 (folder S2)	0.05
0.63 (folder S36)	0.005	0.73 (folder S11)	0.02	0.79 (folder S3)	0.03	0.72 (folder S11)	0.03
0.68 (folder S40)	0.02	0.73 (folder S17)	0.03	0.69 (folder S12)	-0.02	0.57 (folder S23)	0.09
0.93 (folder S10)	0.04	0.64 (folder S18)	0.01	0.71 (folder S22)	0.06	0.71 (folder S29)	0.09
0.64 (folder S19)	0.01	0.56 (folder S28)	0.06	0.71 (folder S28)	0.09	-	-
0.67 (folder S22)	0.008	0.61 (folder S33)	0.04	-	-	-	-
0.67 (folder S 27)	0.01	0.63 (folder S4)	0.08	-	-	-	-
0.68 (folder S34)	0.02	0.60 (folder S21)	0.07	-	-	-	--
0.64 (folder S5)	0.08	-	-	-	-	-	-
0.93 (folder S14)	0.06	-	-	-	-	-	-
0.74 (folder S20)	0.04	-	-	-	-	-	-
0.64 (folder S26)	0.09	-	-	-	-	-	-
0.58 (folder S30)	0.08	-	-	-	-	-	-
0.57 (folder S35)	0.08	-	-	-	-	-	-
0.63 (folder S37)	0.04	-	-	-	-	-	-

Table S9. Relaxation time constant T_d of HP propane gas in excised rabbit lungs at different flip angle in 0.35 T MRI scanner.

In the same manner, we present the whole series of the imaging for the relaxation of HP ^{129}Xe gas in excised rabbit lungs in 0.35 T which are presented in **Figure 4b** with a 4×4 mm^2 pixel size (**Figure S10**). The DICOM images were analyzed using MATLAB. Background subtraction was done slightly differently from that of HP propane gas in excised rabbit lungs. Because the relaxation time of HP ^{129}Xe gas is relatively longer than that of HP propane, it is not possible to subtract any image from the others as this will effectively subtract some of the HP ^{129}Xe signal. So, we choose a region with no HP ^{129}Xe gas intensity which is also a 5×5 region just as chosen in the lungs for the subtraction. Image #4 was taken as the first data point for the plotting of the relaxation data as we can clearly ascertain that the lungs were fully inflated at this point and there was no more gas flow into the excised rabbit lungs. Images #4 to #16 were used in relaxation plot (shown as ROI analysis in **Figure 4b)**. During analysis, the signal intensities of the images were computed and those of #4 to #16 (**Table S10**) were used in the plot of the ROI analysis using Origin.

Time (s)	Signal intensity (a.u)
0	2124.52
1.12	1980.20
2.24	1645.76
3.36	1571.76
4.48	1246.48
5.60	1371.52
6.72	1040.16
7.84	996.96
8.96	819.12
10.08	859.04
11.20	720.28
12.32	618.80
13.44	484.36

Table S10. Signal intensities of scan #4 to scan #16 of excised rabbit lungs at 0.35 T used in the ROI analysis shown in **Figure 4b.**

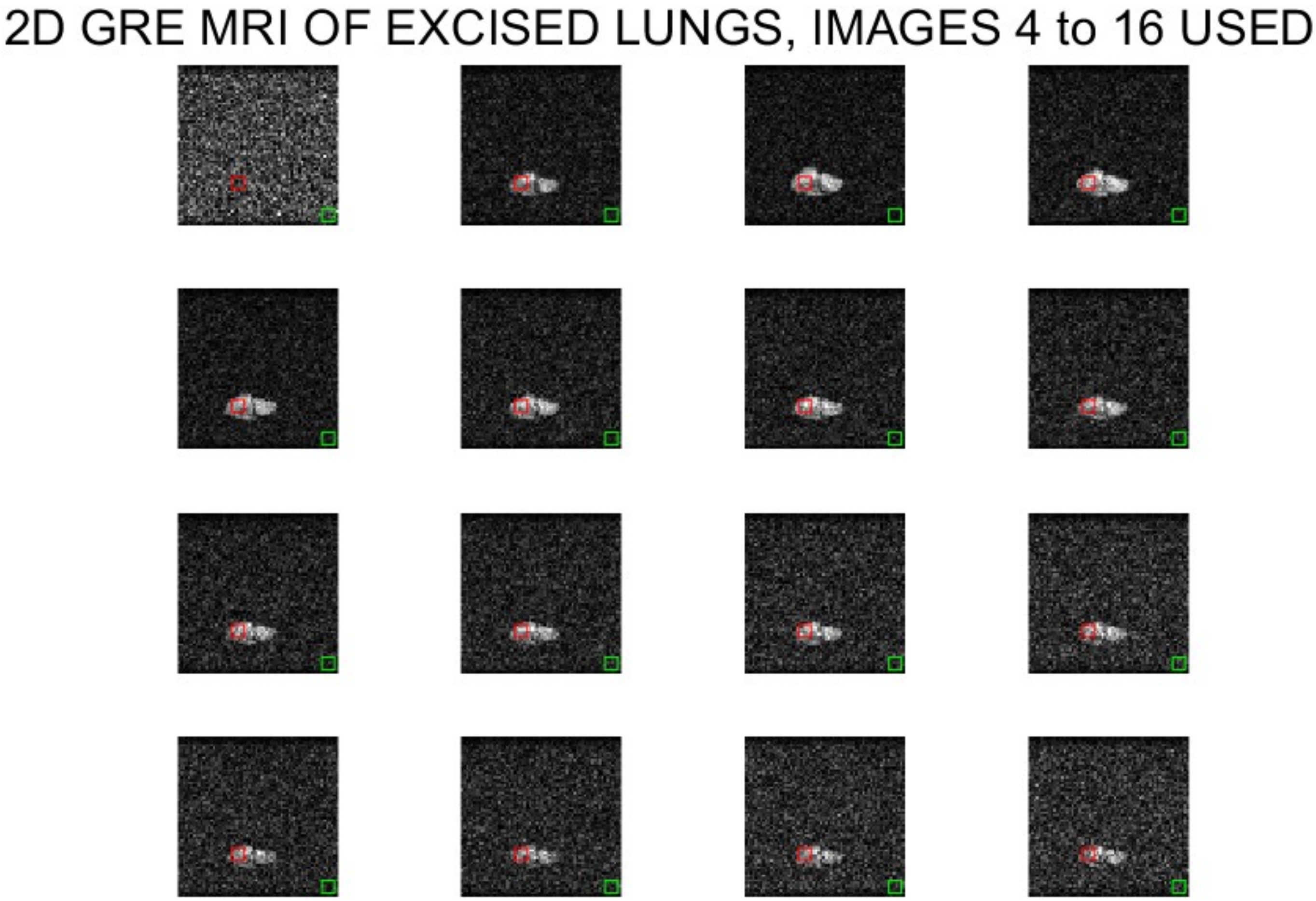

Figure S10. Time series of slice-selective 2D GRE images of HP ^{129}Xe gas injected into excised rabbit lungs, acquired by utilizing a 0.35 T MRI scanner and a knee RF coil. Axial-projection scans of HP ^{129}Xe gas signal decay in the lungs recorded using FA = 5° at 1.12 s temporal resolution. The imaging parameters employed were as follows: 64×64 imaging matrix and 256×256 mm^2 field of view (FOV), 50 mm slice thickness, TE = 17.52 ms, and TR = 8.54 ms. The red box highlights the 5×5 pixel matrix with the intensity region which serves as our (kept same throughout the images) while the green box highlights the noise region (same 5×5 region slected throughout all the images). The average intensity gotten by dividing the signal intensity region by the RMS of the noise region is the signal intensities that was plotted as region of interest (ROI) shown in **Figure 4b.**

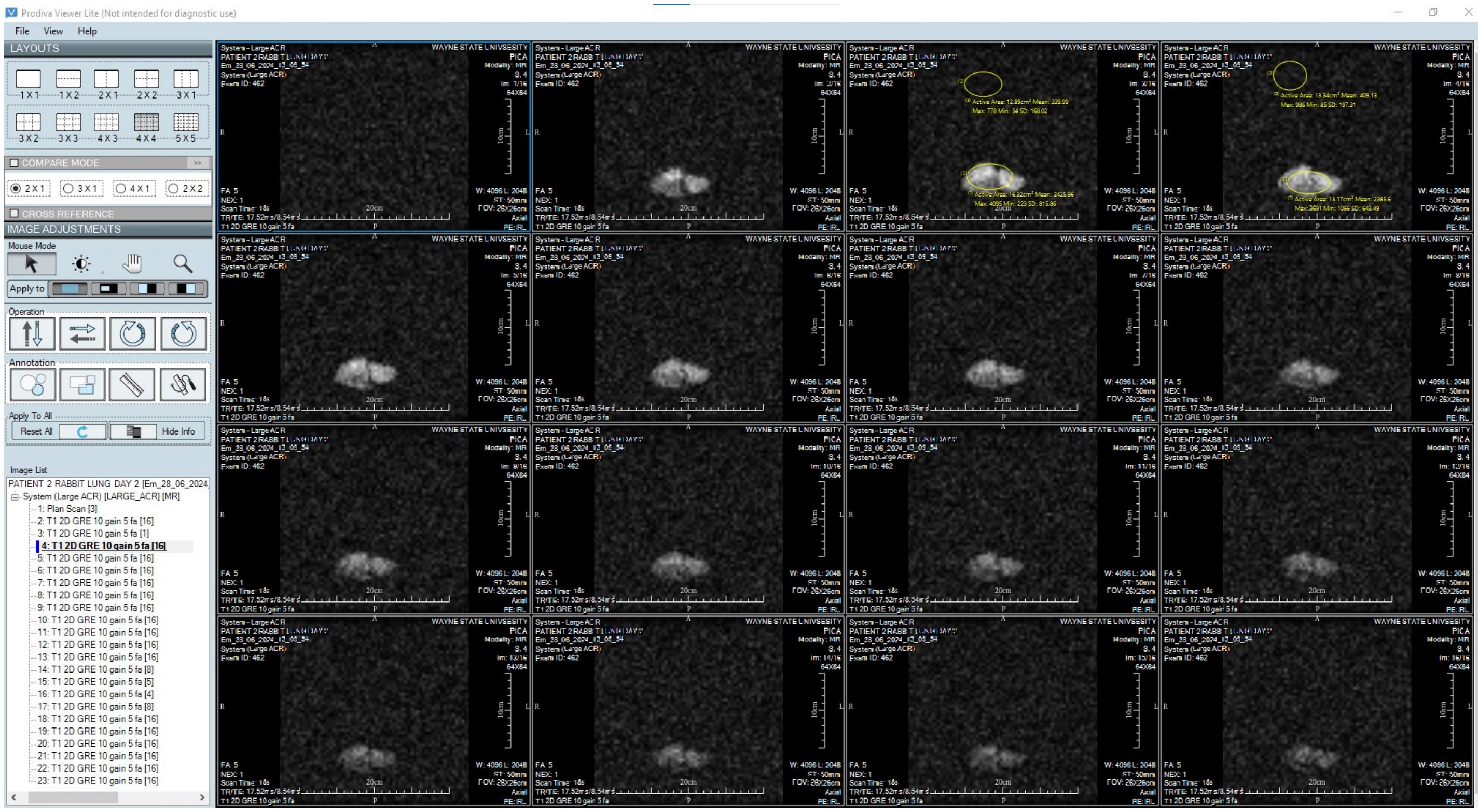

Figure S11. Screenshot of images processed by the MRI scanner software during the experiment presented in **Figure 4b** (axial projection, 4×4 mm^2 pixel size) without any data post-processing.

The data for the reproducibility plot for HP ^{129}Xe gas in excised rabbit lungs at 5° flip angle in 0.35 T clinical MRI scanner is presented below in **Table S11.** Signal intensities values were acquired from images of interest during MATLAB processing of the DICOM files and these intensities were later plotted agaimst time in Origin to get a relaxation decay constant as well as error bar values. Thereafter, the average value and standard deviation of the relaxation time constants at each flip angle was calculated and this average value is what is presented in the histogram in **Figure 4b.**

Table S11. Relaxation time constant T_d of HP ^{129}Xe gas in excised rabbit lungs in 0.35 T MRI scanner.

**FA 5°**	
T_d **(s)**	**Error bar (s)**
9.34 (folder S23)	0.35
9.95 (folder S4)	0.48
13.36 (folder S6)	0.70

9. Higher Resolution MRI scanning of excised rabbit lungs with HP propane gas at 0.35 T

In this section, we present the whole series of the imaging with HP propane gas in excised rabbit lungs in 0.35 T which are presented in **Figure 5** with a 2×2 mm^2 pixel size (**Figure S12**). The DICOM images were analyzed using MATLAB. Background subtraction was done by subtracting the 5th image in the series from images #1 through #16.

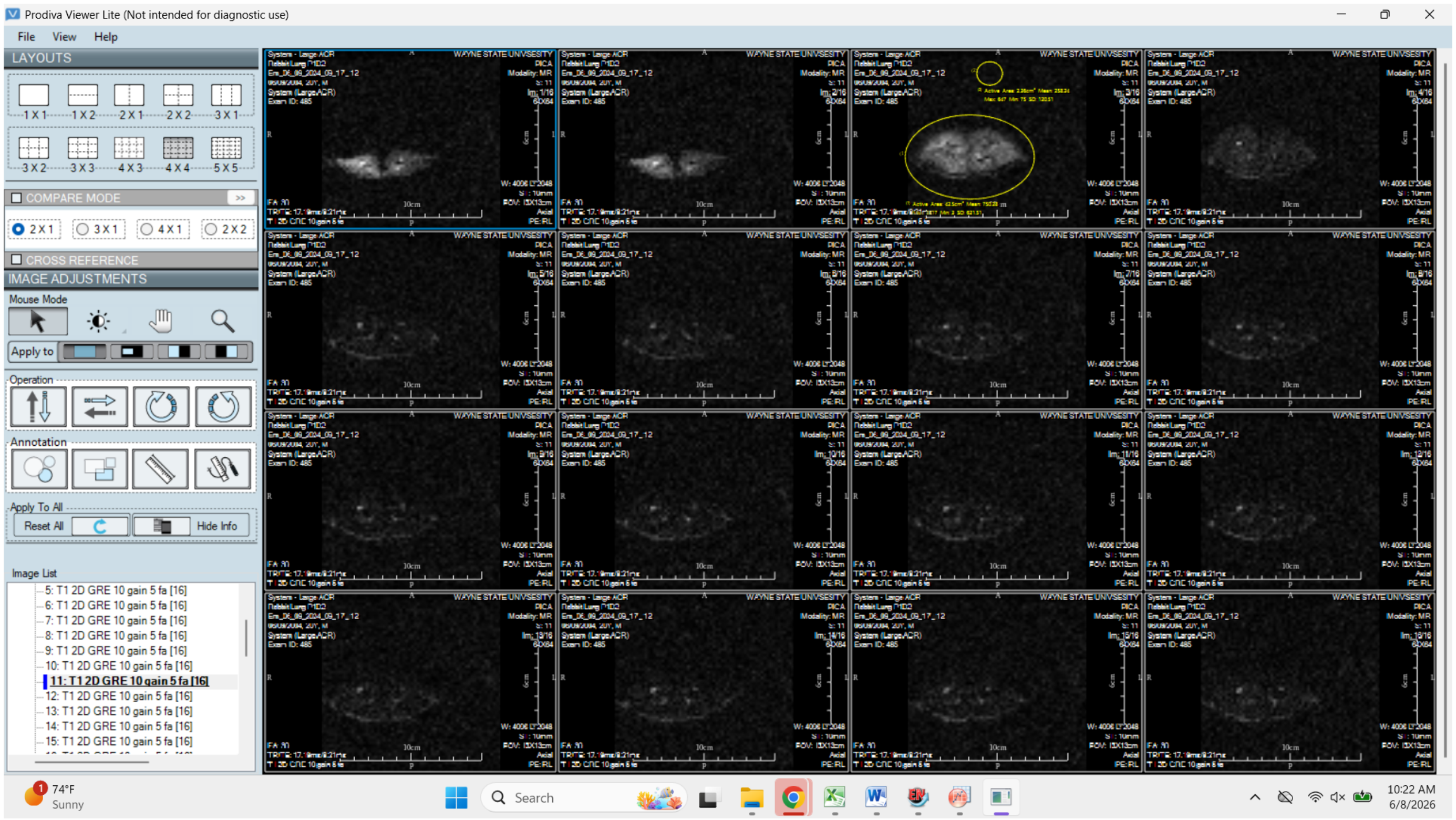

Figure S12. Screenshot of images processed by the MRI scanner software during the experiment presented in **Figure 5** (axial projection, 2×2 mm^2 pixel size) without any data post-processing.

10. MATLAB code for gas-phase T_1 relaxation dynamics studies

Here, we discuss automated data analysis protocol using MATLAB (R2021a). All the data recorded using 1.4 T NMR spectrometer (NMR Pro 60, Nanalysis, New-Zealand) used in 1.4 T ROI Analysis in **Figure 3b** and **Figure S1** were analyzed using the following script. Quotation marks ('Reference Data') are used to show directories and variable names. Comments and operation (preceded with "%" symbol), are shown in the script as a guide to understanding of the code structure. *i.e.,* `% this is a comment`.

The corresponding HP propane gas signal data (64 scans) are previously stored in a folder named as "HP data" and the thermally polarized propane signal reference data are stored in a folder named as "Reference data" which will appear in the script below. According to the pseudo-2D acquisition method, 64 scans are recorded for the HP state for a single experimental run in an accumulated data acquisition fashion. Therefore, we perform a de-accumulation of data prior to calculating their integrated intensities. First, we clean the workspace to make sure it is not filled with unwanted commands/functions from previous runs. Some additional small functions and scripts used for these calculations are given in another folder and are directed using *addpath*. Line broadening, phase correction and baseline correction are performed before the data integration begins. The script below is written in such a way that 64 HP signal plots and their corresponding thermal spectrum are created and imported as PDFs with a CSV file that contains the signal intensities and signal enhancement values. For relaxation data analysis, a build-up curve is created for all the 64 HP signal data files and a separate decay curve is fitted for selected data range of interest (given in "bound 7" and "bound 8" in the code) using an exponential decay function[1-2]

```
% Code for analysis of pseudo-2D NMR Pro data
clear;
close all; clc; clf;

%Addpath for processing functions
addpath("C:\Users\propane\gan_func");

%Short function designed to make whatever folder this script is run from the new working directory
%Useful if this is to be copied and executed in each experiment folder
%scl

%% Variables

lb = 0.5;           %line broadening (Hz)
phase = 20;         %first order phase correction (deg)

%% Import thermal reference data from JCAMP-DX format

%Move to thermal reference data folder
cd 'Reference Data'

%Determine number of spectrum files in thermal experiment folder
m = length(dir) - 2;

%Tabulate all thermal spectrum filenames
tfiles = dir('*.dx');
for j = 1:m
     filearray(j) = strcat(tfiles(j).folder + "\" + tfiles(j).name);
     thermJCAMPStruct{j} = jcampread(filearray(j));
end

for j = 1:m

thermjcampblock = thermJCAMPStruct{j}.Blocks;
thermtimedata = thermJCAMPStruct{j}.Blocks.XData; thermtimedata = (thermtimedata)';

if j == 1
thermrealdata = thermJCAMPStruct{j}.Blocks(1).YData; thermrealdata = (thermrealdata)';
thermimagdata = thermJCAMPStruct{j}.Blocks(2).YData; thermimagdata = (thermimagdata)';
elseif j >= 2
thermrealdata = j*thermJCAMPStruct{j}.Blocks(1).YData - (j-1)*thermJCAMPStruct{j-1}.Blocks(1).YData;
thermrealdata = (thermrealdata)';
thermimagdata = j*thermJCAMPStruct{j}.Blocks(2).YData - (j-1)*thermJCAMPStruct{j-1}.Blocks(2).YData;
thermimagdata = (thermimagdata)';
```

```
end

%Identify relevant acquisition paramaters from struct
thermjcampnotes = thermJCAMPStruct{j}.Notes;
O1Pt = str2double(thermjcampnotes(13,2));       %frequency offset for spectrum center
Ppmt = str2double(thermjcampnotes(8,2));        %frequency in MHz
dwellt = thermtimedata(2)-thermtimedata(1);     %dwell time (difference between time data)
swt = 1/dwellt;                                 %spectral width in Hz
swpt = swt/Ppmt;                                %convert sw to ppm

% [therm,thermdir] = uigetfile('*.*','Select thermal reference .dx spectrum file');
% cd(thermdir)
% %NOTE: jcampread function requires MATLAB Bioinformatics Toolbox
% ThermJCAMPStruct = jcampread(therm);
% %Alternative syntax for fixed-file reference
% %JCAMPStruct = jcampread('pablo_debug_0070_signal.dx');
%
% %Identify time, real and imaginary vectors from struct
% thermjcampblock = ThermJCAMPStruct.Blocks;
% thermtimedata = thermjcampblock.XData; thermtimedata = (thermtimedata)';
% thermrealdata = thermjcampblock(1).YData; thermrealdata = (thermrealdata)';
% thermimagdata = thermjcampblock(2).YData; thermimagdata = (thermimagdata)';

%Define number of points * 4 for zero-filling
td = length(thermtimedata);
npt = td*4;
%Format data into real and imaginary parts to perfom complex conjugation
fidt = thermrealdata + 1i*thermimagdata;

%Perform zero-filling, line-broadening and exp. multiplication
fidt = zf(fidt,npt);
fidt = em(fidt,lb,swt);

%Perform FFT of data and swap center of k-space
spectherm = swap(real(ph(fft(conj(fidt)),phase,0)));
axt = O1Pt+(swpt/2):-(swpt/npt):O1Pt-(swpt/2)+(swpt/npt);     %x-axis absolute frequency (ppm)

%Baseline correct magnitude spectrum
specthermend = spectherm(round(npt*0.75):npt);
specthermbc = mean(specthermend);
spectherm = spectherm - specthermbc;

%Plot 1H magnitude spectrum
fig1 = plot(axt,spectherm,'b-');
set(gca,'XDir','rev')
axis([-2 10 -Inf Inf])      %set axis scale from -2 to 10 ppm
axis on;
xlabel('^1H Frequency (ppm)','FontSize',16,'FontWeight','bold')
ylabel('^1H NMR Magnitude (a.u.)','FontSize',16,'FontWeight','bold')
title('Thermal ^1H NMR Magnitude Spectrum from FID','FontSize',14,'FontWeight','bold')

cd ../
cd 'CSV Data'

%Write processed thermal spectral data to CSV format
axt = (axt)';
thermdatafull = [axt,spectherm];
thermtxt = num2str(j);
writematrix(thermdatafull,strcat('Thermal FFT Data -' + " " + thermtxt + '.csv'))

cd ../
cd 'PDF 1D Spectra'
%Save processed thermal spectrum to PDF format
thermname = strcat('Thermal FFT Data -' + " " + thermtxt);
print(thermname,'-dpdf')

%Calculate thermal reference integral from user boundaries
bound1 = 1271; bound2 = 1420;           %lower/upper intergration bounds
intdatat = spectherm(bound1:bound2);
intranget = axt(bound1:bound2);

%Perform trapezoidal integral of thermal 1H peak
Stherm(j) = trapz(intdatat);
[val, idx] = max(intdatat);
Stherm_max=max(intdatat);
```

```
cd ../
cd 'Reference Data'

end

%Calculate average thermal spectral integral from de-accumulated averages
Stherm_avg = mean(Stherm);

%% Import HP data from JCAMP-DX format

%Move to HP spectral data folder
cd ../
cd 'HP Data'

%Determine number of spectrum files in experiment folder
n = length(dir) - 2;

%Preallocate matrix sizes for speed
SHa = zeros(1,n);SHb = zeros(1,n);
EHa = zeros(1,n);EHb = zeros(1,n);
ExpDataALL = cell(length(n),10);

%Tabulate all spectrum filenames
files = dir('*.dx');
for k = 1:n
     filearray(k) = strcat(files(k).folder + "\" + files(k).name);
     HPJCAMPStruct{k} = jcampread(filearray(k));
end

for k = 1:n

HPjcampblock = HPJCAMPStruct{k}.Blocks;
HPtimedata = HPJCAMPStruct{k}.Blocks.XData; HPtimedata = (HPtimedata)';

if k == 1
HPrealdata = HPJCAMPStruct{k}.Blocks(1).YData; HPrealdata = (HPrealdata)';
HPimagdata = HPJCAMPStruct{k}.Blocks(2).YData; HPimagdata = (HPimagdata)';
elseif k >= 2
HPrealdata = k*HPJCAMPStruct{k}.Blocks(1).YData - (k-1)*HPJCAMPStruct{k-1}.Blocks(1).YData; HPrealdata =
(HPrealdata)';
HPimagdata = k*HPJCAMPStruct{k}.Blocks(2).YData - (k-1)*HPJCAMPStruct{k-1}.Blocks(2).YData; HPimagdata =
(HPimagdata)';
end

%Identify relevant acquisition paramaters from struct
HPjcampnotes = HPJCAMPStruct{k}.Notes;
O1PHP = str2double(HPjcampnotes(13,2));       %frequency offset for spectrum center
PpmHP = str2double(HPjcampnotes(8,2));        %frequency in MHz
dwellHP = HPtimedata(2)-HPtimedata(1);        %dwell time (difference between time data)
swHP = 1/dwellHP;                                 %spectral width in Hz
swpHP = swHP/PpmHP;                                %convert sw to ppm

%Define number of points * 4 for zero-filling
tdHP = length(HPtimedata);
npHP = td*4;
%Format data into real and imaginary parts to perfom complex conjugation
fidHP = HPrealdata + 1i*HPimagdata;

%Perform zero-filling, line-broadening and exp. multiplication
fidHP = zf(fidHP,npHP);
fidHP = em(fidHP,lb,swHP);

%Perform FFT of data and swap center of k-space
specHP = swap(real(ph(fft(conj(fidHP)),phase,0)));
axHP = O1PHP+(swpHP/2):-(swpHP/npHP):O1PHP-(swpHP/2)+(swpHP/npHP);     %x-axis absolute frequency (ppm)

%Baseline correct magnitude spectrum
specHPend = specHP(round(npHP*0.75):npHP);
specHPbc = mean(specHPend);
specHP = specHP - specHPbc;

%Plot 1H magnitude spectrum
figure(2)
fig2 = plot(axHP,specHP,'r-');
set(gca,'XDir','rev')
axis([-2 10 -Inf Inf])      %set axis scale from -2 to 10 ppm
```

```
axis on;
xlabel('^1H Frequency (ppm)','FontSize',16,'FontWeight','bold')
ylabel('^1H NMR Magnitude (a.u.)','FontSize',16,'FontWeight','bold')
title("HP ^1H NMR Magnitude Spectrum from FID - Exp #" + k,'FontSize',14,'FontWeight','bold')

%Write processed thermal spectral data to CSV format
axHP = (axHP)';
HPdatafull = [axHP,specHP];
HPtxt = num2str(k);

cd ../
cd 'CSV Data'
writematrix(HPdatafull,strcat('HP FFT Data -' + " " + HPtxt + '.csv'))

cd ../
cd 'PDF 1D Spectra'
%Save processed thermal spectrum to PDF format
HPname = strcat('HP FFT Data -' + " " + HPtxt);
print(HPname,'-dpdf')

%Calculate HP reference integral from user boundaries
bound3 = 1257; bound4 = 1354;           %lower/upper Ha intergration bounds
bound5 = 1354; bound6 = 1418;           %lower/upper Hb intergration bounds
intdataHa = specHP(bound3:bound4);
intdataHb = specHP(bound5:bound6);
intrangeHa = axt(bound3:bound4);
intrangeHb = axt(bound5:bound6);

%Perform trapezoidal integral of HP 1H peak
SHa(k) = trapz(intdataHa);
SHb(k) = trapz(intdataHb);

%Calculate enhancement factor of HP 1H peak
EHa(k) = (SHa(k)/Stherm_avg)*8;
EHb(k) = (SHb(k)/Stherm_avg)*8;

%Print current experiment Ha/Hb enhancement values to Command Window9
fprintf('Exp. %1.0f - Ha Signal Enhancement = %4.3f, Hb Signal Enhancement = %4.3f\n',k,EHa(k),EHb(k))

cd ../
cd 'HP Data'

end

%Convert data to column arrays for tabulation
SHa = (SHa)'; SHb = (SHb)';
EHa = (EHa)'; EHb = (EHb)';

%% Plot signal enhancement vs. time

%Define experimental timestep
Duration = 33.47;                   %total scan duration in seconds
TimeStep = Duration/(n-1);          %time space between scans
Exptime = 0:TimeStep:Duration;      %creates linear array of time points
Exptime = (Exptime)';

figure(3)
fig3 = plot(Exptime,EHa,'kx');
hold on
plot(Exptime,EHb,'rx')
axis on;
axis([0 Duration -Inf Inf])
xlabel('Time (seconds)','FontSize',16,'FontWeight','bold')
ylabel('^1H NMR Signal Enhancement Factor','FontSize',16,'FontWeight','bold')
title('HP Propane ^1H NMR Dynamic Run','FontSize',14,'FontWeight','bold')

cd ../
%Save processed thermal spectrum to PDF format
Expname = 'Exp Build and Decay';
print(Expname,'-dpdf')

%% Subplot T1 decay data

%Select bounds for fitting
bound7 = 13; bound8 =40;
ExptimeT1 = Exptime(bound7:bound8);
```

```
EHaT1 = EHa(bound7:bound8);
EHbT1 = EHb(bound7:bound8);
t = length(ExptimeT1);

%Define Exp1 exponential decay fitting function
fHa = fit(ExptimeT1,EHaT1,'exp1');
fHb = fit(ExptimeT1,EHbT1,'exp1');

%Determine fitting coefficients, Pmax and uncertainty
coeffsHa = coeffvalues(fHa);
coeffsHb = coeffvalues(fHb);
% EHamax = coeffsHa(1);
confHa = confint(fHa); confHb = confint(fHb);

%Determine relaxation rate constants and uncertainty
T1valHa = abs(1/coeffsHa(2));
T1valHb = abs(1/coeffsHb(2));
xErrHa = abs(1/confHa(1,2)-(1/confHa(2,2)))/2;
xErrHb = abs(1/confHb(1,2)-(1/confHb(2,2)))/2;

%Create string arrays for labelling
T1valtxtHa = num2str(T1valHa);
T1valtxtHb = num2str(T1valHb);
xErrtxtHa = num2str(xErrHa);
xErrtxtHb = num2str(xErrHb);

%Concatenate strings to generate buildup & fit characteristics
fiteqtxt = 'y = a*exp(bx)';
figtxtHa = strcat('T_1(H_a) ='  + " " + T1valtxtHa + " " + '+/-' + " " + xErrtxtHa + " " + 'secs');
figtxtHb = strcat('T_1(H_b) ='  + " " + T1valtxtHb + " " + '+/-' + " " + xErrtxtHb + " " + 'secs');

%Create new plot for relaxation curve
figure(4)
plot(fHa,'k--',ExptimeT1,EHaT1,'ks');
hold on
plot(fHb,'r--',ExptimeT1,EHbT1,'rs');

axis on;
axis([ExptimeT1(1) ExptimeT1(t) -Inf Inf])
ax = gca; ax.FontSize = 14;
xlabel('Relaxation Time (secs)','FontSize',16,'FontWeight','bold')
ylabel('^1H NMR Signal Enhancement','FontSize',16,'FontWeight','bold')
title("^1H T_1 Relaxation",'FontSize',16,'FontWeight','bold')
legend('Location','NorthEastOutside')
xpos = ExptimeT1(6);
yposHa = (max(EHaT1)*0.8); yposHb = (min(EHbT1)*0.8);
text(xpos,yposHa,figtxtHa,'FontSize',14)
text(xpos,yposHb,figtxtHb,'Color','red','FontSize',14)
set(gcf,'Position',[100 100 750 500])

%Save snapshot of T1 data to PDF format
T1name = 'Exp T1 Decay';
print(T1name,'-dpdf')

%% Write all relevant data to file

%Assign thermal integral and T1 values & errors to master cell array
for StoreLoop = 1:n
    ExpDataALL{StoreLoop,1} = Exptime(StoreLoop);
    ExpDataALL{StoreLoop,2} = Stherm_avg;
    ExpDataALL{StoreLoop,3} = SHa(StoreLoop);
    ExpDataALL{StoreLoop,4} = EHa(StoreLoop);
    ExpDataALL{StoreLoop,5} = SHb(StoreLoop);
    ExpDataALL{StoreLoop,6} = EHb(StoreLoop);
    ExpDataALL{StoreLoop,7} = T1valHa;
    ExpDataALL{StoreLoop,8} = xErrHa;
    ExpDataALL{StoreLoop,9} = T1valHb;
    ExpDataALL{StoreLoop,10} = xErrHb;
end

%ExpDataALL = [Exptime,SthermTable,SHa,EHa,SHb,EHb,T1valHaTable,xErrHaTable,T1valHbTable,xErrHbTable];
ExpDataCell = cell2table(ExpDataALL,'VariableNames',{'Time' 'Stherm' 'SHa' 'EHa' 'SHb' 'EHb' 'T1 Ha' 'T1 Ha Err'
'T1 Hb' 'T1 Hb Err'});
writetable(ExpDataCell,'KPI.csv')
```

11. MATLAB Post Processing Imaging Analysis Protocol

This section of the supplementary information provides a comprehensive presentation and discussion of the complete automated data analysis protocol implemented in MATLAB for the imaging data. The protocol entails utilizing custom-built functions specifically designed for processing and analyzing data in the DICOM file format. All acquired data are extracted from the dedicated MRI station computer using a flash drive. However, the architecture of the DICOM images varies from one MRI scanner to another. Nevertheless, all the HP propane gas imaging data acquired from the four different MRI scanners used in this study were analyzed using the same technique. A post inflation image (usually with no HP propane gas signal) is subtracted from all other images to create a difference image. Thereafter specific images are selected for the plotting of the mono-exponential decay fit. During the image processing, a specific region in the image is selected which is analyzed for the signal intensities. These signal intensities is outputted in a tabular format. This table of intensities is then plotted in Origin using mono-exponetial decay fit to get the corresponding relaxation constant of the HP propane gas in each of the MRI scanner studied.

For HP ^{129}Xe gas imaging data analysis protocol, a slightly different approach was employed as already discussed above. Background subtraction was done slightly differently from that of HP propane gas in excised rabbit lungs; hence the MATLAB code was modified. Because the relaxation time of HP ^{129}Xe gas is relatively longer it is not possible to subtract any image from the others as this will effectively subtract some of the HP ^{129}Xe signal. So, we chose a region with no HP ^{129}Xe gas signal which is also a 5×5 region just as chosen in the lungs for the subtraction.

Throughout the section, variable names and directories will be denoted within quotation marks, such as "Image_Subtraction". In-line comments are incorporated to enhance comprehension of the script's structure and operation. These comments are denoted by the % symbol in MATLAB and are displayed in green font to distinguish them from the code itself.

The MATLAB code should be in the folder where the DICOM files are stored.

Mean Signal-to-Noise Ratio (SNR): All image pixels are analyzed for their intensities. First, the region of the image (8×8 pixels in size), where no signal is seen by a user is selected to compute the RMS noise value. This RMS noise value is employed for identifying pixels that contain a signal. When a pixel intensity exceeds a threshold value (computed based on the RMS noise value), the pixel signal is added to the overall calculation of SNR (specifically, the signal is added to the total sum of all signal values from all pixels exceeded the threshold value); in case if the pixel intensity is below the threshold value, it is discarded from the SNR calculation. Next, the mean signal is computed by finding the mean values of all the pixels exceeding the threshold value. Finally, the SNR is computed by dividing the mean signal value by the value of RMS noise. This approach mitigates the need for the region of interest (ROI) selection by an end user.[2-3]

12. MATLAB code for difference image processing of 2D GRE MRI data obtained with HP propane gas in phantom and excised rabbit lungs at 0.35 T MRI scanner

I) File storage format and architecture

This section of the SI presents and discusses the currently utilized format for file and folder naming of MR image analysis using MATLAB processing software. Data from all experiments is saved in a uniform fashion to ensure reproducibility. One complete set of experiments at the 0.35 T MRI scanner can run up to 40 individual scans. All HP propane images were taken using 16 repetitions as described in the main text. All these 16 scans for an individual run are saved inside the folder named S(X), X = number of sequences. DICOM files are saved as I000000M (M = scan number/image number).

After transferring the image data in DICOM file format from the computer, the user selects a sequence with 16 scans that is under investigation. For example, S17 is a scan that is used for acquiring axial HP propane

images in **Figure 3a**. Copy this file to a new analysis folder named "**Figure 3a**". The folder should contain data tagged as **S17**

- SX (X = number of scan)
    - N number of DICOM files, Where N represents number of scans (16) used during the experiment.

```
cla;
clear;
close all;
% parameters to ENTER
FOV=256;                              %! enter FOV from the image information
matrix=64;                            % enter matrix size from the image information
fa = 5;                               % flip angle value: will be used for display only
slice = 50;                           % slide thickness value: will be used for display only
noise_threshold = 10;                 % thresholding for image presentation: everything less than RMS noise * this value will be dropped
subt_image_number = 10;               % image number to be subtracted
exp_number = 17;                      % experiment number: : will be used for display only
date_value = '08/08/2024';            % date: : will be used for display only
gas_value = 'propane';                % type of gas (e.g., propane): : will be used for display only
subject = 'phantom sphere';           % type of gas (e.g., rabbit lungs): : will be used for display only
x_position = 8;                       % x-position for the output of key imaging parameters
y_position = 20;                      % y-position for the output of key imaging parameters
%computed parameters
slice_string = int2str(slice);        % converts slice thickness value to a string
fa_string = int2str(fa);              % converts FA value to a string
fa_string = append('FA=',fa_string);  % finished FA string composition
matrix_string = int2str(matrix);      % converts matrix size to a string
fov_string = int2str(FOV);            % converts FOV value to a string
exp_string = int2str(exp_number);     % converts exp number to a string
exp_string = append('S',exp_string);  % creates S number as a string
subt_string = int2str(subt_image_number);   % converts # of image to be subtracted into a string
date_string = date_value;             % converts date into a string
gas_string = gas_value;               % converts gas type into a string
subject_string = subject;             % converts subject type into a string

% Specify the paths of the DICOM files
dicomPaths = {
'I0000001.dcm',
'I0000002.dcm',
'I0000003.dcm',
'I0000004.dcm',
'I0000005.dcm',
'I0000006.dcm',
'I0000007.dcm',
'I0000008.dcm',
'I0000009.dcm',
'I0000010.dcm',
'I0000011.dcm',
'I0000012.dcm',
'I0000013.dcm',
'I0000014.dcm',
'I0000015.dcm',
'I0000016.dcm',
};

% Read the DICOM files
dicomFiles = cell(16, 1);
for i = 1:16
    dicomFiles{i} = double(dicomread(dicomPaths{i}));
end

% Create scan #99 using the scan to be subtracted
for i = subt_image_number:subt_image_number
    for x = 1:matrix
    for y = 1:matrix
        dicomFiles{99}(x, y) = dicomFiles{i}(x, y);      % assigning the matrix number and its elements
    end
    end
end
% Perfoming the background subtraction
for i = 1:16
```

```
    for x = 1:matrix
    for y = 1:matrix
        dicomFiles_diff{i}(x, y) = dicomFiles{i}(x, y) - dicomFiles{99}(x, y);      % making the subtraction
    end
    end
end

% Create a subplot to plot the resized image (one at a time)
figure;

for i = 1:16
    % Resize the image to 768x768
    counter = 0;        % resets the counter for the number of noise calculation
    signalsum = 0;      % resets the signal pixel count for noise calculation
    original_image{i}=dicomFiles_diff{i};   % loads the images
    resized_image = imresize(double(dicomFiles_diff{i}), [768, 768]); % loads the images
    % compute noise:
    noise_image = original_image{i}(4:11, 10:17);    % selects the region for noise computations
    offset = mean(noise_image);                     % computing the mean for each column of the image matrix
    offset1 = mean(offset);                     % computing the mean for all elements of the image matrix
    noise_diff = noise_image-offset1;
    noiserms = (rms(noise_image-offset1));        % computing RMS noise of each column of the noise matrix after subtracting the mean value
    noiserms1 = (rms(noiserms));                  % computing RMS noise of the image matrix
    noise_counter = noiserms1*noise_threshold;     % computing noise value for thresholding using the factor noise_threshold
    % compute average SNR using noise thresholding
for k = 1:matrix
for j = 1:matrix
    if original_image{i}(k, j) > noise_counter          % compares pixel value to noise threshold
        signalsum = signalsum + original_image{i}(k, j)/noiserms1;  % if the pixel value is greater than the threshold value, it is added to the total SNR sum
        counter = counter +1;                           % counts the number of pixels that exceeded the noise threshold value
    end
end
end
    SNR_average = signalsum/counter;               % computes the average SNR of all signal pixels that have exceeded the noise threshold value
    snr_string = int2str(SNR_average);           % converts SNR value to a string
    %%
    %image plotting
    imshow(resized_image, [], 'XData', [0, FOV], 'YData', [0, FOV]);           % broadcasting the image in the figure dispplay (one at a time)
    YourText = sprintf(append('FOV=',fov_string,'x',fov_string,' mm2 \nmatrix=',matrix_string,'x',matrix_string,'\nslice=',slice_string,' mm\nSNR=',snr_string)); %
yellow text
    hText = text(x_position,y_position,YourText,'Color',[1 1 0],'FontSize',18); %yellow text position and color %! changes second number to 25(from 20) as my texts
were popping out of the figure frame at the top
    caxis([noise_counter/4, 4095-noise_counter/4]); % setting the threshold for image visualization
    ax = gca;   %standard axes
    axis on;    % axes to be displays (ON)
    i_string = int2str(i);  % creates string value of i (the loop counter)
    title_name = append(date_string,' ',exp_string,', ',gas_string,', ',subject_string,'\n',fa_string,', difference image=',i_string,'-',subt_string);
    title(sprintf(title_name));             % title statement using title_name file
    ax.FontSize = 14;                      % font size of the axes
    ax.TickDir = 'out';                   % ticks are out not in
    ax.TickLength = [0.015 0.015];          % ticks' length
    ax.XAxis.MajorTickChild.LineWidth = 2;  % x axis tickmark thickness = 2
    ax.YAxis.MajorTickChild.LineWidth = 2;  % y axis tickmark thickness = 2
    xlabel('x (mm)')        % x axis label
    ylabel('y (mm)')        % y axis label
    colorbar                % displays the color bar
    colormap(hot);          %  color mode: other good color modes are: 'gray' and 'jet'
    filename = append(exp_string,'_difference_',i_string,'-',subt_string,'.pdf');
    exportgraphics(gcf, sprintf(filename), 'ContentType', 'vector'); %!
    line wont work on my matlab. errors says unrecognized function or
    %variable 'exportgraphics'
    %saveas(gcf, sprintf(filename)); %!line added to be able to save images generated during analysis
end
```

13. MATLAB code for gas-phase T_d relaxation dynamics studies with HP propane gas in excised rabbit lungs and phantoms at 0.35 T

```
clc; % Clears the command window.
clear; % Clears all variables from the workspace.
close all; % Closes all open figure windows.
% Parameters to ENTER
FOV=256;                        % enter FOV from the image information
matrix=64;                       % enter matrix size from the image information
fa = 5;                          % flip angle value: will be used for display only
exp_number = 17;                   % experiment number: : will be used for display only
scan_time = 1.12; % Time per image scan in seconds.
dicom_start = 5; % The starting DICOM image number.
subt_image_number = dicom_start + 9; % The DICOM image number to be subtracted.
x_start = 31; % Starting x-coordinate for the signal intensity region.
x_end = 34; % Ending x-coordinate for the signal intensity region.
y_start = 33; % Starting y-coordinate for the signal intensity region.
y_end = 36;  % Ending y-coordinate for the signal intensity region.
region_size = 4; % Size of the region for calculating T1.

% Computed parameters
x_start_string = int2str(x_start);        % converts slice thickness value to a string
x_end_string = int2str(x_end);
y_start_string = int2str(y_start);
y_end_string = int2str(y_end);
dicom_start_string = int2str(dicom_start);
subt_image_number_string = int2str(subt_image_number);
fa_string = int2str(fa);              % converts FA value to a string
exp_string = int2str(exp_number);      % converts exp number to a string
exp_string = append('S',exp_string);      % creates S number as a string;              % finished FA string composition
fov_string = int2str(FOV);            % converts FOV value to a string
matrix_string = int2str(matrix);      % converts matrix size to a string
num_file = dicom_start:subt_image_number; % Creates an array of image numbers for processing.
dicom_string = int2str(dicom_start); % Converts the starting DICOM number to a string.
subt_string = int2str(subt_image_number); % Converts the subtraction image number to a string.

% Specify the paths of the DICOM files
dicomPaths = {
  'I0000001.dcm',
'I0000002.dcm',
'I0000003.dcm',
'I0000004.dcm',
'I0000005.dcm',
'I0000006.dcm',
'I0000007.dcm',
'I0000008.dcm',
'I0000009.dcm',
'I0000010.dcm',
'I0000011.dcm',
'I0000012.dcm',
'I0000013.dcm',
'I0000014.dcm',
'I0000015.dcm',
'I0000016.dcm',

}; % List of DICOM file paths.

% Read the DICOM files
dicomFiles = cell(16, 1); % Preallocate a cell array for the DICOM images.
for i = 1:16
   dicomFiles{i} = double(dicomread(dicomPaths{i})); % Read each DICOM file into the array.
end

% Calculate average signal intensity for each image after subtraction
signal_intensities = zeros(1, 16); % Preallocate an array for signal intensities.
for i = 1:16
   subtractedImage{i} = dicomFiles{i} - dicomFiles{subt_image_number}; % Subtract the specified image.
   signal_T1_image = subtractedImage{i}(y_start:y_end, x_start:x_end); % Extract the region for signal intensity.
   signal_intensities(i) = (mean(signal_T1_image(:))); % Compute the mean signal intensity.
end

% Image plotting one at a time but plotted together in same page
figure; % Create a new figure window.
for i = 1:16
```

```
    resized_image = subtractedImage{i}; % Prepare the subtracted image for plotting.
    subplot(4, 4, i); % Create a subplot in a 4x4 grid.
    imshow(resized_image, []); % Display the subtracted image.

    %xlabel('x (pixel)'); % Label the x-axis.
    %ylabel('y (pixel)'); % Label the y-axis.

    % Highlight the selected region
    hold on; % Retain current plot when adding new elements.
    rectangle('Position', [x_start, y_start, region_size, region_size], 'EdgeColor', 'red', 'LineWidth', 0.5); % Draw a rectangle around the selected region.
    hold off; % Release the plot hold.

    title_name = append('2D GRE MRI OF PHANTOM, IMAGES', ' ', dicom_string, ' ', 'to', ' ', subt_string,' ','USED'); % Create a title for the figure.
    sgtitle(title_name); % Set a super title for the entire figure.

    filename = append('T1_Images.pdf'); % Define the filename for saving images.
    exportgraphics(gcf, sprintf(filename), 'ContentType', 'vector');

    filename = append('T1_Images.jpg'); % Define the filename for saving images.
    exportgraphics(gcf, sprintf(filename), 'ContentType', 'vector'); %!
    %saveas(gcf, sprintf(filename)); % Save the current figure as a PDF.
end

% Define the data points for fitting
xData = [0, 1*scan_time, 2*scan_time, 3*scan_time, 4*scan_time, 5*scan_time, 6*scan_time, 7*scan_time, 8*scan_time, 9*scan_time]; % Create x-data points based
on scan time.
yData = signal_intensities(num_file); % Get corresponding signal intensities.
xData = (xData)'; % Transpose xData for fitting.
yData = (yData)'; % Transpose yData for fitting.
filename = append('signal_intenvstime.xlsx'); % Define filename for exporting data to Excel.
xlswrite(filename, xData, 'Sheet1', 'A1'); % Write xData (time) to the first column of Excel.
xlswrite(filename, yData, 'Sheet1', 'B1'); % Write yData (signal intensities) to the second column of Excel.

% Fit the data to the T1 function: SNR = A * exp(-TE / T1)
%mymodel = fittype('a+b*exp(-c*x)');
mymodel=@(t1,b,c,x) (b+c*exp(-x/t1));
f = fit(xData, yData, mymodel,'Startpoint',[1 10 1000]); % Perform exponential fitting on the data.
% Determine fitting coefficients, max and uncertainty
coeffs = coeffvalues(f); % Get the fitting coefficients.
max = round(coeffs(3), 2); % Round the maximum value from the fit.
bounds = confint(f); % Get the confidence intervals for the fitting.
yErr = round(abs((bounds(2,3)-bounds(1,3))/2), 2); % Calculate the uncertainty in y.

% Determine relaxation rate constants and uncertainty
t1val = round(abs(coeffs(1)), 2); % Calculate T1 relaxation time from the fit.
xErr = round((bounds(2,1)-bounds(1,1))/2, 2); % Calculate the uncertainty in x.

% Determine constants and uncertainty
maxtxt = num2str(max); % Convert max value to string.
t1valtxt = num2str(t1val); % Convert T1 value to string.
yErrtxt = num2str(yErr); % Convert y error to string.
xErrtxt = num2str(xErr); % Convert x error to string.

% Concatenate strings to generate buildup & fit characteristics
fiteqtxt = 'y = a*exp(bx)'; % Define the fitting equation.
figtxt1 = strcat('max =', " ", maxtxt, " ", char(177), " ", yErrtxt); % Create text for max and uncertainty.
figtxt2 = strcat('T_1 =', " ", t1valtxt, " ", char(177), " ", xErrtxt, " ", 's'); % Create text for T1 value and uncertainty.

% Plot the signal intensities values against time
figure; % Create a new figure for plotting.
plot(f, 'r--', xData, yData, 'ks'); % Plot the fitted curve and data points.
xlabel('Time (s)'); % Label the x-axis.
ylabel('Avg. Intensities (a.u.)'); % Label the y-axis.
%title('Difference Image Intensities vs Time'); % Set the plot title.
title_name = append(exp_string,' ','ROI ANALYSIS');
title(sprintf(title_name));           % title statement using title_name
legend('Data', 'Exponential Fit'); % Add a legend to the plot.
xpos = (1);
ypos = (1);
Mytext = sprintf(append('fitmodel=(t_1,b,c,x) (b+c*exp(-x/t_1))'));
YourText = sprintf(append('FOV=',fov_string,'x',fov_string,' mm2 \nmatrix=',matrix_string,'x',matrix_string,'\nregion(y,x)=',y_start_string,':',y_end_string,'
',x_start_string,':',x_end_string,'\nFA=',fa_string,'\nimagesub=',dicom_start_string,':',subt_image_number_string));  % yellow text
hText = text(0.5*xpos, 1500*ypos, YourText,'color','magenta','FontSize',16); %yellow text position and color
hText = text(0.5*xpos, 800*ypos, figtxt1, 'color', 'green','FontSize',16); % Add max text to the plot with color chosen.
hText = text(0.5*xpos, 500*ypos, figtxt2, 'color', 'blue','FontSize',16); % Add T1 text to the plot with color chosen.
```

```
hText = text(0.1*xpos, -100*ypos, Mytext, 'color', 'red', 'FontSize',16); % Add T1 text to the plot with color chosen.
%grid on; % Turn on the grid for the plot.

% Save the figure as a PDF file
%saveas(gcf, 'Difference Image T1 plot.pdf'); % Save the current figure as a PDF.
filename = append('Difference Image T1 plot','.pdf');
exportgraphics(gcf, sprintf(filename), 'ContentType', 'vector'); %!

filename = append('Difference Image T1 plot','.jpg');
exportgraphics(gcf, sprintf(filename), 'ContentType', 'vector'); %!
```

14. MATLAB code for raw imaging data with HP ^{129}Xe gas in excised rabbit lungs at 0.35 T

The MATLAB code presented here was utilized for images presented in **Figure 4b and Figure 5**.

```
cla;
clear;
close all;
% parameters to ENTER
FOV=256;                    % enter FOV from the image information
matrix=64;                  % enter matrix size from the image information
fa = 5;                     % flip angle value: will be used for display only
slice = 50;                 % slide thickness value: will be used for display only
noise_threshold = 10;       % thresholding for image presentation: everything less than RMS noise * this value will be dropped
exp_number = 4;             % experiment number: : will be used for display only
date_value = '06/28/2023';  % date: : will be used for display only
gas_value = 'xenon';        % type of gas (e.g., propane): : will be used for display only
subject = 'rabbit lungs';   % type of gas (e.g., rabbit lungs): : will be used for display only
x_position = 8;             % x-position for the output of key imaging parameters
y_position = 25;            % y-position for the output of key imaging parameters
%computed parameters
slice_string = int2str(slice);      % converts slice thickness value to a string
fa_string = int2str(fa);            % converts FA value to a string
fa_string = append('FA=',fa_string);        % finished FA string composition
matrix_string = int2str(matrix);    % converts matrix size to a string
fov_string = int2str(FOV);          % converts FOV value to a string
exp_string = int2str(exp_number);   % converts exp number to a string
exp_string = append('S',exp_string);    % creates S number as a string
date_string = date_value;   % converts date into a string
gas_string = gas_value;     % converts gas type into a string
subject_string = subject;   % converts subject type into a string


% Specify the paths of the DICOM files
dicomPaths = {
'I0000001.dcm',
'I0000002.dcm',
'I0000003.dcm',
'I0000004.dcm',
'I0000005.dcm',
'I0000006.dcm',
'I0000007.dcm',
'I0000008.dcm',
'I0000009.dcm',
'I0000009.dcm',
'I0000010.dcm',
'I0000011.dcm',
'I0000012.dcm',
'I0000013.dcm',
'I0000014.dcm',
'I0000015.dcm',
'I0000016.dcm',
};

% Read the DICOM files
dicomFiles = cell(16, 1);
for i = 1:16
    dicomFiles{i} = double(dicomread(dicomPaths{i}));
end

% reate a subplot to plot the resized image (one at a time)
figure;
```

```
for i = 1:16
    % Resize the image to 768x768
    counter = 0;        % resets the counter for the number of noise calculation
    signalsum = 0;      % resets the signal pixel count for noise calculation
    original_image{i}=dicomFiles{i};    % loads the images
    resized_image = imresize(double(dicomFiles{i}), [768, 768]);     % loads the images
    % compute noise:
    noise_image = original_image{i}(4:11, 10:17);    % selects the region for noise computations
    offset = mean(noise_image);                     % computing the mean for each column of the image matrix
    offset1 = mean(offset);                     % computing the mean for all elements of the image matrix
    noise_diff = noise_image-offset1;
    noiserms = (rms(noise_image-offset1));        % computing RMS noise of each column of the noise matrix after subtracting the mean value
    noiserms1 = (rms(noiserms));                  % computing RMS noise of the image matrix
    noise_counter = noiserms1*noise_threshold;     % computing noise value for thresholding using the factor noise_threshold
    % compute average SNR using noise thresholding
for k = 1:matrix
for j = 1:matrix
    if original_image{i}(k, j) > noise_counter         % compares pixel value to noise threshold
        signalsum = signalsum + original_image{i}(k, j)/noiserms1;  % if the pixel value is greater than the threshold value, it is added to the total SNR sum
        counter = counter +1;                          % counts the number of pixels that exceeded the noise threshold value
    end
end
end
    SNR_average = signalsum/counter;               % computes the average SNR of all signal pixels that have exceeded the noise threshold value
    snr_string = int2str(SNR_average);           % converts SNR value to a string
    %image plotting
    imshow(resized_image, [], 'XData', [0, FOV], 'YData', [0, FOV]);       % broadcasting the image in the figure dispplay (one at a time)
    YourText = sprintf(append('FOV=',fov_string,'x',fov_string,' mm2 \nmatrix=',matrix_string,'x',matrix_string,'\nslice=',slice_string,' mm\nSNR=',snr_string));  % 
yellow text
    hText = text(x_position,y_position,YourText,'Color',[1 1 0],'FontSize',18); %yellow text position and color %! changes second number to 25(from 20) as my texts 
were popping out of the figure frame at the top
    caxis([noise_counter/2, 4095-noise_counter/2]);  % setting the threshold for image visualization
    ax = gca;   %standard axes
    axis on;    % axes to be displays (ON)
    i_string = int2str(i);  % creates string value of i (the loop counter)
    title_name = append(date_string,' ',exp_string,', ',gas_string,', ',subject_string,'\n',fa_string,', image=',i_string);
    title(sprintf(title_name));             % title statement using title_name file
    ax.FontSize = 14;                      % font size of the axes
    ax.TickDir = 'out';                   % ticks are out not in
    ax.TickLength = [0.015 0.015];         % ticks' length
    ax.XAxis.MajorTickChild.LineWidth = 2;  % x axis tickmark thickness = 2
    ax.YAxis.MajorTickChild.LineWidth = 2;  % y axis tickmark thickness = 2
    xlabel('x (mm)')        % x axis label
    ylabel('y (mm)')        % y axis label
    colorbar               % displays the color bar
    colormap(hot);          %  color mode: other good color modes are: 'gray' and 'jet'
    filename = append(exp_string,'_',i_string,'.pdf');
    exportgraphics(gcf, sprintf(filename), 'ContentType', 'vector'); %!
    %line wont work on my matlab. errors says unrecognized function or
    %variable 'exportgraphics'
    %saveas(gcf, sprintf(filename)); %!line added to be able to save images generated during analysis
end
```

15. MATLAB code for gas-phase T_d relaxation dynamics studies with HP ^{129}Xe gas in excised rabbit lungs at 0.35 T

```
clc; % Clears the command window.
clear; % Clears all variables from the workspace.
close all; % Closes all open figure windows.
% Parameters to ENTER
FOV=256;                            % enter FOV from the image information
matrix=64;                          % enter matrix size from the image information
fa = 5;                           % flip angle value: will be used for display only
exp_number = 4;                     % experiment number: : will be used for display only
scan_time = 1.12; % Time per image scan in seconds.
dicom_start = 4; % The starting DICOM image number.
%subt_image_number = dicom_start + 10; % The DICOM image number to be subtracted.
x_start = 22; % Starting x-coordinate for the signal intensity region.
x_end = 26; % Ending x-coordinate for the signal intensity region.
y_start = 45; % Starting y-coordinate for the signal intensity region.
y_end = 49;  % Ending y-coordinate for the signal intensity region.
x1_start = 58; % Starting x-coordinate for the signal intensity region.
```

```
x1_end = 62; % Ending x-coordinate for the signal intensity region.
y1_start = 58; % Starting y-coordinate for the signal intensity region.
y1_end = 62;  % Ending y-coordinate for the signal intensity region.
region_size = 5; % Size of the region for calculating T1.

% Computed parameters
x_start_string = int2str(x_start);          % converts slice thickness value to a string
x_end_string = int2str(x_end);
y_start_string = int2str(y_start);
y_end_string = int2str(y_end);
dicom_start_string = int2str(dicom_start);
%subt_image_number_string = int2str(subt_image_number);
fa_string = int2str(fa);               % converts FA value to a string
exp_string = int2str(exp_number);       % converts exp number to a string
exp_string = append('S',exp_string);       % creates S number as a string;               % finished FA string composition
fov_string = int2str(FOV);            % converts FOV value to a string
matrix_string = int2str(matrix);      % converts matrix size to a string
%num_file = dicom_start:subt_image_number; % Creates an array of image numbers for processing.
dicom_string = int2str(dicom_start); % Converts the starting DICOM number to a string.
%subt_string = int2str(subt_image_number); % Converts the subtraction image number to a string.

% Specify the paths of the DICOM files
dicomPaths = {
   'I0000001.dcm',
'I0000002.dcm',
'I0000003.dcm',
'I0000004.dcm',
'I0000005.dcm',
'I0000006.dcm',
'I0000007.dcm',
'I0000008.dcm',
'I0000009.dcm',
'I0000010.dcm',
'I0000011.dcm',
'I0000012.dcm',
'I0000013.dcm',
'I0000014.dcm',
'I0000015.dcm',
'I0000016.dcm',

}; % List of DICOM file paths.

% Read the DICOM files
dicomFiles = cell(16, 1); % Preallocate a cell array for the DICOM images.
for i = 1:16
    dicomFiles{i} = double(dicomread(dicomPaths{i})); % Read each DICOM file into the array.
end

% Calculate average signal intensity for each image after subtraction
signal_intensities = zeros(1, 16); % Preallocate an array for signal intensities.
for i = 1:16
    subtractedImage{i} = dicomFiles{i}; %- dicomFiles{subt_image_number}; % Subtract the specified image.
    signal_T1_image = subtractedImage{i}(y_start:y_end, x_start:x_end)-subtractedImage{i}(y1_start:y1_end, x1_start:x1_end); % Extract the region for signal
intensity.
    signal_intensities(i) = (mean(signal_T1_image(:))); % Compute the mean signal intensity.
end

% Image plotting one at a time but plotted together in same page
figure; % Create a new figure window.
for i = 1:16
    resized_image = subtractedImage{i}; % Prepare the subtracted image for plotting.
    subplot(4, 4, i); % Create a subplot in a 4x4 grid.
    imshow(resized_image, []); % Display the subtracted image.

    %xlabel('x (pixel)'); % Label the x-axis.
    %ylabel('y (pixel)'); % Label the y-axis.

    % Highlight the selected region
    hold on; % Retain current plot when adding new elements.
    rectangle('Position', [x_start, y_start, region_size, region_size], 'EdgeColor', 'red', 'LineWidth', 0.5); % Draw a rectangle around the selected region.
    rectangle('Position', [x1_start, y1_start, region_size, region_size], 'EdgeColor', 'green', 'LineWidth', 0.5); % Draw a rectangle around the selected region.
    hold off; % Release the plot hold.

    title_name = append('2D GRE MRI OF EXCISED LUNGS, IMAGES', ' ', dicom_string, ' ', 'to', ' ', "16",' ','USED'); % Create a title for the figure.
    sgtitle(title_name); % Set a super title for the entire figure.
```

```
    filename = append('T1_Images.pdf'); % Define the filename for saving images.
    exportgraphics(gcf, sprintf(filename), 'ContentType', 'vector');

    filename = append('T1_Images.jpg'); % Define the filename for saving images.
    exportgraphics(gcf, sprintf(filename), 'ContentType', 'vector'); %!
    %saveas(gcf, sprintf(filename)); % Save the current figure as a PDF.
end

% Define the data points for fitting
xData = [0, 1*scan_time, 2*scan_time, 3*scan_time, 4*scan_time, 5*scan_time, 6*scan_time, 7*scan_time, 8*scan_time, 9*scan_time, 10*scan_time, 11*scan_time,
12*scan_time];%, 13*scan_time, 14*scan_time]; % Create x-data points based on scan time.
yData = signal_intensities(4:end); % Get corresponding signal intensities.
xData = (xData)'; % Transpose xData for fitting.
yData = (yData)'; % Transpose yData for fitting.
filename = append('signal_intenvstime.xlsx'); % Define filename for exporting data to Excel.
xlswrite(filename, xData, 'Sheet1', 'A1'); % Write xData (time) to the first column of Excel.
xlswrite(filename, yData, 'Sheet1', 'B1'); % Write yData (signal intensities) to the second column of Excel.

% Fit the data to the T1 function: SNR = A * exp(-TE / T1)
%mymodel = fittype('a+b*exp(-c*x)');
mymodel=@(t1,b,c,x) (b+c*exp(-x/t1));
f = fit(xData, yData, mymodel,'Startpoint',[1 10 1000]); % Perform exponential fitting on the data.
% Determine fitting coefficients, max and uncertainty
coeffs = coeffvalues(f); % Get the fitting coefficients.
max = round(coeffs(3), 2); % Round the maximum value from the fit.
bounds = confint(f); % Get the confidence intervals for the fitting.
yErr = round(abs((bounds(2,3)-bounds(1,3))/2), 2); % Calculate the uncertainty in y.

% Determine relaxation rate constants and uncertainty
t1val = round(abs(coeffs(1)), 2); % Calculate T1 relaxation time from the fit.
xErr = round((bounds(2,1)-bounds(1,1))/2, 2); % Calculate the uncertainty in x.

% Determine constants and uncertainty
maxtxt = num2str(max); % Convert max value to string.
t1valtxt = num2str(t1val); % Convert T1 value to string.
yErrtxt = num2str(yErr); % Convert y error to string.
xErrtxt = num2str(xErr); % Convert x error to string.

% Concatenate strings to generate buildup & fit characteristics
fiteqtxt = 'y = a*exp(bx)'; % Define the fitting equation.
figtxt1 = strcat('max =', " ", maxtxt, " ", char(177), " ", yErrtxt); % Create text for max and uncertainty.
figtxt2 = strcat('T_1 =', " ", t1valtxt, " ", char(177), " ", xErrtxt, " ", 's'); % Create text for T1 value and uncertainty.

% Plot the signal intensities values against time
figure; % Create a new figure for plotting.
plot(f, 'r--', xData, yData, 'ks'); % Plot the fitted curve and data points.
xlabel('Time (s)'); % Label the x-axis.
ylabel('Avg. Intensities (a.u.)'); % Label the y-axis.
%title('Difference Image Intensities vs Time'); % Set the plot title.
title_name = append(exp_string,' ','ROI ANALYSIS');
title(sprintf(title_name));            % title statement using title_name
legend('Data', 'Exponential Fit'); % Add a legend to the plot.
xpos = (1);
ypos = (1);
Mytext = sprintf(append('fitmodel=(t_1,b,c,x) (b+c*exp(-x/t_1))'));
YourText = sprintf(append('FOV=',fov_string,'x',fov_string,' mm2 \nmatrix=',matrix_string,'x',matrix_string,'\nregion(y,x)=',y_start_string,':',y_end_string,'
',x_start_string,':',x_end_string,'\nFA=',fa_string,'\nimagesub=',dicom_start_string,':','16'));  % yellow text
hText = text(0.5*xpos, 2000*ypos, YourText,'color','magenta','FontSize',16); %yellow text position and color
hText = text(0.5*xpos, 1600*ypos, figtxt1, 'color', 'green','FontSize',16); % Add max text to the plot with color chosen.
hText = text(0.5*xpos, 1200*ypos, figtxt2, 'color', 'blue','FontSize',16); % Add T1 text to the plot with color chosen.
hText = text(0.1*xpos, 1000*ypos, Mytext, 'color', 'red', 'FontSize',16); % Add T1 text to the plot with color chosen.
%grid on; % Turn on the grid for the plot.

% Save the figure as a PDF file
%saveas(gcf, 'Difference Image T1 plot.pdf'); % Save the current figure as a PDF.
filename = append('Difference Image T1 plot','.pdf');
exportgraphics(gcf, sprintf(filename), 'ContentType', 'vector'); %!

filename = append('Difference Image T1 plot','.jpg');
exportgraphics(gcf, sprintf(filename), 'ContentType', 'vector'); %!
```

16. MATLAB code for gas-phase T_d relaxation dynamics in phantom at 0.55 T MRI scanner

I) File storage format and architecture

This section of the SI presents and discusses the currently utilized format for file and folder naming of MR image analysis using MATLAB processing software. To ensure reproducibility between data sets, it is expected that data from all experiments is saved in a uniform fashion. All HP propane images at 0.55 T were taken using various repetitions ranging from 8 to 16. DICOM files are saved as Phantom_Test.MR.Plummer_Hyperpo.19.M (M is a string of number comprising of year, time and other unique identifier for each scan).

After transferring the image data in DICOM file format from the computer, the user selects group specific DICOM file based on the numbers of the DICOM folder that is labeled by the user to create a sequence with the correct number of scans that is under investigation. For example, S4 is a scan that is used for acquiring axial HP propane images in the **Figure 3b**.

- SX (X = number of scan)
    - N number of DICOM files, Where N represents number of scans (in this case 8) used during the experiment.

```
clc; % Clears the command window.
clear; % Clears all variables from the workspace.
close all; % Closes all open figure windows.
% Parameters to ENTER
FOV=320;                          % enter FOV from the image information
matrix=64;                        % enter matrix size from the image information
fa = 5;                           % flip angle value: will be used for display only
exp_number = 4;                   % experiment number: : will be used for display only
scan_time = 0.293; % Time per image scan in seconds.
dicom_start = 1; % The starting DICOM image number.
subt_image_number = dicom_start + 7; % The DICOM image number to be subtracted.
x_start = 63; % Starting x-coordinate for the signal intensity region.
x_end = 67; % Ending x-coordinate for the signal intensity region.
y_start = 23; % Starting y-coordinate for the signal intensity region.
y_end = 27;  % Ending y-coordinate for the signal intensity region.
region_size = 5; % Size of the region for calculating T1.

% Computed parameters
x_start_string = int2str(x_start);        % converts slice thickness value to a string
x_end_string = int2str(x_end);
y_start_string = int2str(y_start);
y_end_string = int2str(y_end);
dicom_start_string = int2str(dicom_start);
subt_image_number_string = int2str(subt_image_number);
fa_string = int2str(fa);             % converts FA value to a string
exp_string = int2str(exp_number);      % converts exp number to a string
exp_string = append('S',exp_string);      % creates S number as a string;             % finished FA string composition
fov_string = int2str(FOV);          % converts FOV value to a string
matrix_string = int2str(matrix);      % converts matrix size to a string
num_file = dicom_start:subt_image_number; % Creates an array of image numbers for processing.
dicom_string = int2str(dicom_start); % Converts the starting DICOM number to a string.
subt_string = int2str(subt_image_number); % Converts the subtraction image number to a string.

% Specify the paths of the DICOM files
dicomPaths = {
   'Phantom_Test.MR.Plummer_Hyperpo.4.3.2024.07.30.11.27.00.275.27822333.dcm',
    'Phantom_Test.MR.Plummer_Hyperpo.4.6.2024.07.30.11.27.00.275.27822366.dcm',
    'Phantom_Test.MR.Plummer_Hyperpo.4.12.2024.07.30.11.27.00.275.27822432.dcm',
    'Phantom_Test.MR.Plummer_Hyperpo.4.14.2024.07.30.11.27.00.275.27822454.dcm',
    'Phantom_Test.MR.Plummer_Hyperpo.4.15.2024.07.30.11.27.00.275.27822465.dcm',
    'Phantom_Test.MR.Plummer_Hyperpo.4.16.2024.07.30.11.27.00.275.27822476.dcm',
    'Phantom_Test.MR.Plummer_Hyperpo.4.17.2024.07.30.11.27.00.275.27822487.dcm',
    'Phantom_Test.MR.Plummer_Hyperpo.4.22.2024.07.30.11.27.00.275.27822542.dcm',

    }; % List of DICOM file paths.

% Read the DICOM files
```

```
dicomFiles = cell(8, 1); % Preallocate a cell array for the DICOM images.
for i = 1:8
    dicomFiles{i} = double(dicomread(dicomPaths{i})); % Read each DICOM file into the array.
end

% Calculate average signal intensity for each image after subtraction
signal_intensities = zeros(1, 8); % Preallocate an array for signal intensities.
for i = 1:8
    subtractedImage{i} = dicomFiles{i} - dicomFiles{subt_image_number}; % Subtract the specified image.
    signal_T1_image = subtractedImage{i}(y_start:y_end, x_start:x_end); % Extract the region for signal intensity.
    signal_intensities(i) = (mean(signal_T1_image(:))); % Compute the mean signal intensity.
end

% Image plotting one at a time but plotted together in same page
figure; % Create a new figure window.
for i = 1:8
    resized_image = subtractedImage{i}; % Prepare the subtracted image for plotting.
    subplot(4, 4, i); % Create a subplot in a 4x4 grid.
    imshow(resized_image, []); % Display the subtracted image.

    %xlabel('x (pixel)'); % Label the x-axis.
    %ylabel('y (pixel)'); % Label the y-axis.

    % Highlight the selected region
    hold on; % Retain current plot when adding new elements.
    rectangle('Position', [x_start, y_start, region_size, region_size], 'EdgeColor', 'red', 'LineWidth', 0.5); % Draw a rectangle around the selected region.
    hold off; % Release the plot hold.

    title_name = append('2D GRE MRI OF PHANTOM, IMAGES', ' ', dicom_string, ' ', 'to', ' ', subt_string,' ','USED'); % Create a title for the figure.
    sgtitle(title_name); % Set a super title for the entire figure.

    filename = append('T1_Images.pdf'); % Define the filename for saving images.
    exportgraphics(gcf, sprintf(filename), 'ContentType', 'vector');

    filename = append('T1_Images.jpg'); % Define the filename for saving images.
    exportgraphics(gcf, sprintf(filename), 'ContentType', 'vector'); %!
    %saveas(gcf, sprintf(filename)); % Save the current figure as a PDF.
end

% Define the data points for fitting
xData = [0, 1*scan_time, 2*scan_time, 3*scan_time, 4*scan_time, 5*scan_time, 6*scan_time, 7*scan_time];%, 8*scan_time, 9*scan_time]; % Create x-data points based on scan time.
yData = signal_intensities(num_file); % Get corresponding signal intensities.
xData = (xData)'; % Transpose xData for fitting.
yData = (yData)'; % Transpose yData for fitting.
filename = append('signal_intenvstime.xlsx'); % Define filename for exporting data to Excel.
xlswrite(filename, xData, 'Sheet1', 'A1'); % Write xData (time) to the first column of Excel.
xlswrite(filename, yData, 'Sheet1', 'B1'); % Write yData (signal intensities) to the second column of Excel.

% Fit the data to the T1 function: SNR = A * exp(-TE / T1)
%mymodel = fittype('a+b*exp(-c*x)');
mymodel=@(t1,b,c,x) (b+c*exp(-x/t1));
f = fit(xData, yData, mymodel,'Startpoint',[1 10 1000]); % Perform exponential fitting on the data.
% Determine fitting coefficients, max and uncertainty
coeffs = coeffvalues(f); % Get the fitting coefficients.
max = round(coeffs(3), 2); % Round the maximum value from the fit.
bounds = confint(f); % Get the confidence intervals for the fitting.
yErr = round(abs((bounds(2,3)-bounds(1,3))/2), 2); % Calculate the uncertainty in y.

% Determine relaxation rate constants and uncertainty
t1val = round(abs(coeffs(1)), 2); % Calculate T1 relaxation time from the fit.
xErr = round((bounds(2,1)-bounds(1,1))/2, 2); % Calculate the uncertainty in x.

% Determine constants and uncertainty
maxtxt = num2str(max); % Convert max value to string.
t1valtxt = num2str(t1val); % Convert T1 value to string.
yErrtxt = num2str(yErr); % Convert y error to string.
xErrtxt = num2str(xErr); % Convert x error to string.

% Concatenate strings to generate buildup & fit characteristics
fiteqtxt = 'y = a*exp(bx)'; % Define the fitting equation.
figtxt1 = strcat('max =', " ", maxtxt, " ", char(177), " ", yErrtxt); % Create text for max and uncertainty.
figtxt2 = strcat('T_1 =', " ", t1valtxt, " ", char(177), " ", xErrtxt, " ", 's'); % Create text for T1 value and uncertainty.

% Plot the signal intensities values against time
```

```
figure; % Create a new figure for plotting.
plot(f, 'r--', xData, yData, 'ks'); % Plot the fitted curve and data points.
xlabel('Time (s)'); % Label the x-axis.
ylabel('Avg. Intensities (a.u.)'); % Label the y-axis.
%title('Difference Image Intensities vs Time'); % Set the plot title.
title_name = append(exp_string,' ','ROI ANALYSIS');
title(sprintf(title_name));            % title statement using title_name
legend('Data', 'Exponential Fit'); % Add a legend to the plot.
xpos = (1);
ypos = (1);
Mytext = sprintf(append('fitmodel=(t_1,b,c,x) (b+c*exp(-x/t_1))'));
YourText = sprintf(append('FOV=',fov_string,'x',fov_string,' mm2 \nmatrix=',matrix_string,'x',matrix_string,'\nregion(y,x)=',y_start_string,':',y_end_string,'
',x_start_string,':',x_end_string,'\nFA=',fa_string,'\nimagesub=',dicom_start_string,':',subt_image_number_string));  % yellow text
hText = text(0.5*xpos, 20*ypos, YourText,'color','magenta','FontSize',16); %yellow text position and color
hText = text(0.5*xpos, 10*ypos, figtxt1, 'color', 'green','FontSize',16); % Add max text to the plot with color chosen.
hText = text(0.5*xpos, 4*ypos, figtxt2, 'color', 'blue','FontSize',16); % Add T1 text to the plot with color chosen.
hText = text(0*xpos, 1*ypos, Mytext, 'color', 'red', 'FontSize',16); % Add T1 text to the plot with color chosen.
%grid on; % Turn on the grid for the plot.

% Save the figure as a PDF file
%saveas(gcf, 'Difference Image T1 plot.pdf'); % Save the current figure as a PDF.
filename = append('Difference Image T1 plot','.pdf');
exportgraphics(gcf, sprintf(filename), 'ContentType', 'vector'); %!

filename = append('Difference Image T1 plot','.jpg');
exportgraphics(gcf, sprintf(filename), 'ContentType', 'vector'); %!
```

17. MATLAB code for gas-phase T_d relaxation dynamics with HP propane in phantom at 3 T MRI scanner

I) File storage format and architecture

This section of the SI presents and discusses the currently utilized format for file and folder naming of MR image analysis using MATLAB processing software. To ensure reproducibility between data sets, it is expected that data from all experiments is saved in a uniform fashion. All HP propane images on the 3 T MRI scanner were taken using 64 repetitions as described in the main text. All these 64 scans for an individual run are saved in a DICOM file named 'Enlm1' in a sub folder named 'pdata' inside folder labeled numerically, e.g., 1, 2, 4, 6 etc. These numbers represent the sequences.

After transferring the image data in DICOM file format from the MRI, the user selects a sequence that is under investigation. For example, folder 16 is a scan that is used for acquiring axial HP propane images which was used in the 3 T relaxation plot in **Figure 3b**.

```
clc; % Clears the command window.
clear; % Clears all variables from the workspace.
close all; % Closes all open figure windows.
% Parameters to ENTER
FOV=64;                           % enter FOV from the image information
matrix=64;                        % enter matrix size from the image information
fa = 5;                          % flip angle value: will be used for display only
exp_number = 16;                    % experiment number: : will be used for display only
scan_time = 0.147; % Time per image scan in seconds.
dicom_start = 26; % The starting DICOM image number.
subt_image_number = dicom_start + 38; % The DICOM image number to be subtracted.
x_start = 31; % Starting x-coordinate for the signal intensity region.
x_end = 37; % Ending x-coordinate for the signal intensity region.
y_start = 28; % Starting y-coordinate for the signal intensity region.
y_end = 34;  % Ending y-coordinate for the signal intensity region.
region_size = 7; % Size of the region for calculating T1.

% Computed parameters
x_start_string = int2str(x_start);        % converts slice thickness value to a string
x_end_string = int2str(x_end);
y_start_string = int2str(y_start);
```

```
y_end_string = int2str(y_end);
dicom_start_string = int2str(dicom_start);
subt_image_number_string = int2str(subt_image_number);
fa_string = int2str(fa);            % converts FA value to a string
exp_string = int2str(exp_number);     % converts exp number to a string
exp_string = append('S',exp_string);     % creates S number as a string;          % finished FA string composition
fov_string = int2str(FOV);          % converts FOV value to a string
matrix_string = int2str(matrix);     % converts matrix size to a string
num_file = dicom_start:subt_image_number; % Creates an array of image numbers for processing.
dicom_string = int2str(dicom_start); % Converts the starting DICOM number to a string.
subt_string = int2str(subt_image_number); % Converts the subtraction image number to a string.
% Specify the path to your DICOM file
dicomFilePath = 'EnIm1.dcm';

% Initialize an empty 4D array to store the image data
dicomImage = [];

% Read the DICOM file
dicomImage = double(dicomread(dicomFilePath));

% Optionally, you can display additional metadata
dicomInfo = dicominfo(dicomFilePath);
disp(dicomInfo);
% Display DICOM metadata in the Command Window

% Check the dimensions of your DICOM image
% This will show something like [Width, Height, slice, frame]
size(dicomImage)

% Calculate average signal intensity for each image after subtraction
for k = 1:size(dicomImage, 4)
   reference_frame = dicomImage(:,:,subt_image_number);
   subtractedImage{k} = dicomImage(:,:,k) - reference_frame;
   signal_T1_image = subtractedImage{k}(y_start:y_end, x_start:x_end);
   signal_intensities(k) = (mean(signal_T1_image(:))); % Compute the mean signal intensity.
end

% Define the last three data points for fitting
xData = [0, 1*scan_time, 2*scan_time, 3*scan_time, 4*scan_time, 5*scan_time, 6*scan_time, 7*scan_time, 8*scan_time, 9*scan_time, 10*scan_time, 11*scan_time,
12*scan_time, 13*scan_time, 14*scan_time, 15*scan_time, 16*scan_time, 17*scan_time, 18*scan_time, 19*scan_time, 20*scan_time, 21*scan_time, 22*scan_time,
23*scan_time, 24*scan_time, 25*scan_time, 26*scan_time, 27*scan_time, 28*scan_time, 29*scan_time, 30*scan_time, 31*scan_time, 32*scan_time, 33*scan_time,
34*scan_time, 35*scan_time, 36*scan_time, 37*scan_time, 38*scan_time];%, 39*scan_time];%, 40*scan_time];
yData = signal_intensities(num_file);
xData=(xData)';
yData=(yData)';

% Image plotting one at a time but plotted together in same page
figure; % Create a new figure window.
for k = 1:size(dicomImage, 4)
   resized_image = subtractedImage{k}; % Prepare the subtracted image for plotting.
   subplot(9, 8, k); % Create a subplot in a 4x4 grid.
   imshow(resized_image, []); % Display the subtracted image.

   % Highlight the selected region
   hold on; % Retain current plot when adding new elements.
   rectangle('Position', [x_start, y_start, region_size, region_size], 'EdgeColor', 'red', 'LineWidth', 0.5); % Draw a rectangle around the selected region.
   hold off; % Release the plot hold.
   %title([num2str(k),'-' subt_string]);
   title_name = append('2D GRE MRI OF PHANTOM, IMAGES', ' ', dicom_string, ' ', 'to', ' ', subt_string,' ','USED'); % Create a title for the figure.
   sgtitle(title_name); % Set a super title for the entire figure.

   filename = append('T1_Images_7pixel.pdf'); % Define the filename for saving images.
   exportgraphics(gcf, sprintf(filename), 'ContentType', 'vector');

   filename = append('T1_Images_7pixel.jpg'); % Define the filename for saving images.
   exportgraphics(gcf, sprintf(filename), 'ContentType', 'vector'); %!
   %saveas(gcf, sprintf(filename)); % Save the current figure as a PDF.
end

filename = append('signal_intenvstime_7pixel.xlsx'); % Define filename for exporting data to Excel.
xlswrite(filename, xData, 'Sheet1', 'A1'); % Write xData (time) to the first column of Excel.
xlswrite(filename, yData, 'Sheet1', 'B1'); % Write yData (signal intensities) to the second column of Excel.

% Fit the data to the T1 function: SNR = A * exp(-TE / T1)
%mymodel = fittype('a+b*exp(-c*x)');
```

```
mymodel=@(t1,b,c,x) (b+c*exp(-x/t1));
f = fit(xData, yData, mymodel,'Startpoint',[1 10 10000]); % Perform exponential fitting on the data.
% Determine fitting coefficients, max and uncertainty
coeffs = coeffvalues(f); % Get the fitting coefficients.
max = round(coeffs(3), 2); % Round the maximum value from the fit.
bounds = confint(f); % Get the confidence intervals for the fitting.
yErr = round(abs((bounds(2,3)-bounds(1,3))/2), 2); % Calculate the uncertainty in y.

% Determine relaxation rate constants and uncertainty
t1val = round(abs(coeffs(1)), 2); % Calculate T1 relaxation time from the fit.
xErr = round((bounds(2,1)-bounds(1,1))/2, 2); % Calculate the uncertainty in x.

% Determine constants and uncertainty
maxtxt = num2str(max); % Convert max value to string.
t1valtxt = num2str(t1val); % Convert T1 value to string.
yErrtxt = num2str(yErr); % Convert y error to string.
xErrtxt = num2str(xErr); % Convert x error to string.

% Concatenate strings to generate buildup & fit characteristics
%fiteqtxt = 'y = a*exp(bx)'; % Define the fitting equation.
figtxt1 = strcat('max =', " ", maxtxt, " ", char(177), " ", yErrtxt); % Create text for max and uncertainty.
figtxt2 = strcat('T_1 =', " ", t1valtxt, " ", char(177), " ", xErrtxt, " ", 's'); % Create text for T1 value and uncertainty.

% Plot the signal intensities values against time
figure; % Create a new figure for plotting.
plot(f, 'r--', xData, yData, 'ks'); % Plot the fitted curve and data points.
xlabel('Time (s)'); % Label the x-axis.
ylabel('Avg. Intensities (a.u.)'); % Label the y-axis.
%title('Difference Image Intensities vs Time'); % Set the plot title.
title_name = append(exp_string,' ','ROI ANALYSIS 7pixel');
title(sprintf(title_name));            % title statement using title_name
legend('Data', 'Exponential Fit'); % Add a legend to the plot.
xpos = (1);
ypos = (1);
Mytext = sprintf(append('fitmodel=(t_1,b,c,x) (b+c*exp(-x/t_1))'));
YourText = sprintf(append('FOV=',fov_string,'x',fov_string,' mm2 \nmatrix=',matrix_string,'x',matrix_string,'\nregion(y,x)=',y_start_string,':',y_end_string,'
',x_start_string,':',x_end_string,'\nFA=',fa_string,'\nimagesub=',dicom_start_string,':',subt_image_number_string));  % yellow text
hText = text(0.5*xpos, 16000*ypos, YourText,'color','magenta','FontSize',16); %yellow text position and color
hText = text(0.5*xpos, 5000*ypos, figtxt1, 'color', 'green','FontSize',16); % Add max text to the plot with color chosen.
hText = text(0.5*xpos, 3000*ypos, figtxt2, 'color', 'blue','FontSize',16); % Add T1 text to the plot with color chosen.
hText = text(0*xpos, 1*ypos, Mytext, 'color', 'red', 'FontSize',16); % Add T1 text to the plot with color chosen.
%grid on; % Turn on the grid for the plot.


% Save the figure as a JPG file
filename = append('Difference Image T1 7pixel plot','.jpg');
exportgraphics(gcf, sprintf(filename), 'ContentType', 'vector'); %!

% Save the figure as a PDF file
filename = append('Difference Image T1 7pixel plot','.pdf');
exportgraphics(gcf, sprintf(filename), 'ContentType', 'vector'); %!
%saveas(gcf, 'Raw Image T1.pdf');
end
```

18. MATLAB code for gas-phase T_d relaxation dynamics obtained in phantom with HP propane in 7 T MRI scanner

I) File storage format and architecture

This section of the SI presents and discusses the currently utilized format for file and folder naming of MR image analysis using MATLAB processing software. To ensure reproducibility between data sets, it is expected that data from all experiments is saved in a uniform fashion. All HP propane images at 7 T were taken using 32 repetitions as described in the main text. All these 32 scans for an individual run are saved as DICOM files in a folder named ‘dicom’ in a sub folder named ‘pdata’ inside folder labeled numerically, e.g., 1, 2, 4, 6 etc. These numbers represent the sequences. DICOM files are saved as ImM (M = scan number).

After transferring the image data in DICOM file format from the MRI computer, the user selects a sequence with 32 scans that is under investigation. For example, folder 12 is a scan that is used for acquiring axial HP propane images which was used in the 7 T relaxation plot in **Figure 3b**

```
clc; % Clears the command window.
clear; % Clears all variables from the workspace.
close all; % Closes all open figure windows.
% Parameters to ENTER
FOV=96;                              % enter FOV from the image information
matrix=64;                           % enter matrix size from the image information
fa = 5;                              % flip angle value: will be used for display only
exp_number = 12;                     % experiment number: : will be used for display only
scan_time = 0.289; % Time per image scan in seconds.
dicom_start = 11; % The starting DICOM image number.
subt_image_number = dicom_start + 9; % The DICOM image number to be subtracted.
x_start = 34; % Starting x-coordinate for the signal intensity region.
x_end = 38; % Ending x-coordinate for the signal intensity region.
y_start = 36; % Starting y-coordinate for the signal intensity region.
y_end = 40;  % Ending y-coordinate for the signal intensity region.
region_size = 5; % Size of the region for calculating T1.

% Computed parameters
x_start_string = int2str(x_start);         % converts slice thickness value to a string
x_end_string = int2str(x_end);
y_start_string = int2str(y_start);
y_end_string = int2str(y_end);
dicom_start_string = int2str(dicom_start);
subt_image_number_string = int2str(subt_image_number);
fa_string = int2str(fa);              % converts FA value to a string
exp_string = int2str(exp_number);       % converts exp number to a string
exp_string = append('S',exp_string);       % creates S number as a string;              % finished FA string composition
fov_string = int2str(FOV);            % converts FOV value to a string
matrix_string = int2str(matrix);      % converts matrix size to a string
num_file = dicom_start:subt_image_number; % Creates an array of image numbers for processing.
dicom_string = int2str(dicom_start); % Converts the starting DICOM number to a string.
subt_string = int2str(subt_image_number); % Converts the subtraction image number to a string.

% Specify the paths of the DICOM files
dicomPaths = {
    'Im01.dcm',
    'Im02.dcm',
    'Im03.dcm',
    'Im04.dcm',
    'Im05.dcm',
    'Im06.dcm',
    'Im07.dcm',
    'Im08.dcm',
    'Im09.dcm',
    'Im10.dcm',
    'Im11.dcm',
    'Im12.dcm',
    'Im13.dcm',
    'Im14.dcm',
    'Im15.dcm',
    'Im16.dcm'
    'Im17.dcm',
    'Im18.dcm',
    'Im19.dcm',
    'Im20.dcm',
    'Im21.dcm',
    'Im22.dcm',
    'Im23.dcm',
    'Im24.dcm',
    'Im25.dcm',
    'Im26.dcm',
    'Im27.dcm',
    'Im28.dcm',
    'Im29.dcm',
    'Im30.dcm',
    'Im31.dcm',
    'Im32.dcm',
```

```
    }; % List of DICOM file paths.

% Read the DICOM files
dicomFiles = cell(32, 1); % Preallocate a cell array for the DICOM images.
for i = 1:32
    dicomFiles{i} = double(dicomread(dicomPaths{i})); % Read each DICOM file into the array.
end

% Calculate average signal intensity for each image after subtraction
signal_intensities = zeros(1, 32); % Preallocate an array for signal intensities.
for i = 1:32
    subtractedImage{i} = dicomFiles{i} - dicomFiles{subt_image_number}; % Subtract the specified image.
    signal_T1_image = subtractedImage{i}(y_start:y_end, x_start:x_end); % Extract the region for signal intensity.
    signal_intensities(i) = (mean(signal_T1_image(:))); % Compute the mean signal intensity.
end

% Image plotting one at a time but plotted together in same page
figure; % Create a new figure window.
for i = 1:32
    resized_image = subtractedImage{i}; % Prepare the subtracted image for plotting.
    subplot(9, 4, i); % Create a subplot in a 4x4 grid.
    imshow(resized_image, []); % Display the subtracted image.

    %xlabel('x (pixel)'); % Label the x-axis.
    %ylabel('y (pixel)'); % Label the y-axis.

    % Highlight the selected region
    hold on; % Retain current plot when adding new elements.
    rectangle('Position', [x_start, y_start, region_size, region_size], 'EdgeColor', 'red', 'LineWidth', 0.5); % Draw a rectangle around the selected region.
    hold off; % Release the plot hold.

    title_name = append('2D GRE MRI OF PHANTOM, IMAGES', ' ', dicom_string, ' ', 'to', ' ', subt_string,' ','USED'); % Create a title for the figure.
    sgtitle(title_name); % Set a super title for the entire figure.

    filename = append('T1_Images.pdf'); % Define the filename for saving images.
    exportgraphics(gcf, sprintf(filename), 'ContentType', 'vector');

    filename = append('T1_Images.jpg'); % Define the filename for saving images.
    exportgraphics(gcf, sprintf(filename), 'ContentType', 'vector'); %!
    %saveas(gcf, sprintf(filename)); % Save the current figure as a PDF.
end

% Define the data points for fitting
xData = [0, 1*scan_time, 2*scan_time, 3*scan_time, 4*scan_time, 5*scan_time, 6*scan_time, 7*scan_time, 8*scan_time, 9*scan_time]; % Create x-data points based on scan time.
yData = signal_intensities(num_file); % Get corresponding signal intensities.
xData = (xData)'; % Transpose xData for fitting.
yData = (yData)'; % Transpose yData for fitting.
filename = append('signal_intenvstime.xlsx'); % Define filename for exporting data to Excel.
xlswrite(filename, xData, 'Sheet1', 'A1'); % Write xData (time) to the first column of Excel.
xlswrite(filename, yData, 'Sheet1', 'B1'); % Write yData (signal intensities) to the second column of Excel.

% Fit the data to the T1 function: SNR = A * exp(-TE / T1)
%mymodel = fittype('a+b*exp(-c*x)');
mymodel=@(t1,b,c,x) (b+c*exp(-x/t1));
f = fit(xData, yData, mymodel,'Startpoint',[1 10 1000]); % Perform exponential fitting on the data.
% Determine fitting coefficients, max and uncertainty
coeffs = coeffvalues(f); % Get the fitting coefficients.
max = round(coeffs(3), 2); % Round the maximum value from the fit.
bounds = confint(f); % Get the confidence intervals for the fitting.
yErr = round(abs((bounds(2,3)-bounds(1,3))/2), 2); % Calculate the uncertainty in y.

% Determine relaxation rate constants and uncertainty
t1val = round(abs(coeffs(1)), 2); % Calculate T1 relaxation time from the fit.
xErr = round((bounds(2,1)-bounds(1,1))/2, 2); % Calculate the uncertainty in x.

% Determine constants and uncertainty
maxtxt = num2str(max); % Convert max value to string.
t1valtxt = num2str(t1val); % Convert T1 value to string.
yErrtxt = num2str(yErr); % Convert y error to string.
xErrtxt = num2str(xErr); % Convert x error to string.

% Concatenate strings to generate buildup & fit characteristics
fiteqtxt = 'y = a*exp(bx)'; % Define the fitting equation.
figtxt1 = strcat('max =', " ", maxtxt, " ", char(177), " ", yErrtxt); % Create text for max and uncertainty.
```

```
figtxt2 = strcat('T_1 =', " ", t1valtxt, " ", char(177), " ", xErrtxt, " ", 's'); % Create text for T1 value and uncertainty.

% Plot the signal intensities values against time
figure; % Create a new figure for plotting.
plot(f, 'r--', xData, yData, 'ks'); % Plot the fitted curve and data points.
xlabel('Time (s)'); % Label the x-axis.
ylabel('Avg. Intensities (a.u.)'); % Label the y-axis.
%title('Difference Image Intensities vs Time'); % Set the plot title.
title_name = append(exp_string,' ','ROI ANALYSIS');
title(sprintf(title_name));           % title statement using title_name
legend('Data', 'Exponential Fit'); % Add a legend to the plot.
xpos = (1);
ypos = (1);
Mytext = sprintf(append('fitmodel=(t_1,b,c,x) (b+c*exp(-x/t_1))'));
YourText = sprintf(append('FOV=',fov_string,'x',fov_string,' mm2 \nmatrix=',matrix_string,'x',matrix_string,'\nregion(y,x)=',y_start_string,':',y_end_string,'
',x_start_string,':',x_end_string,'\nFA=',fa_string,'\nimagesub=',dicom_start_string,':',subt_image_number_string));  % yellow text
hText = text(0.5*xpos, 3500*ypos, YourText,'color','magenta','FontSize',16); %yellow text position and color
hText = text(0.5*xpos, 2000*ypos, figtxt1, 'color', 'green','FontSize',16); % Add max text to the plot with color chosen.
hText = text(0.5*xpos, 1500*ypos, figtxt2, 'color', 'blue','FontSize',16); % Add T1 text to the plot with color chosen.
hText = text(0.1*xpos, -100*ypos, Mytext, 'color', 'red', 'FontSize',16); % Add T1 text to the plot with color chosen.
%grid on; % Turn on the grid for the plot.

% Save the figure as a PDF file
%saveas(gcf, 'Difference Image T1 plot.pdf'); % Save the current figure as a PDF.
filename = append('Difference Image T1 plot','.pdf');
exportgraphics(gcf, sprintf(filename), 'ContentType', 'vector'); %!

filename = append('Difference Image T1 plot','.jpg');
exportgraphics(gcf, sprintf(filename), 'ContentType', 'vector'); %!
```

19. Data availability statement

All raw DICOM MRI scans that are presented in the main text and the SI, and all spectroscopic data presented in the main text and the SI can be found in the ZIP SI file. The raw MRI data is denoted by the figure/display number, and the spectroscopic data notation is provided in **Figure S1** caption.

20. Detailed sensitivity comparison of HP propane and HP ^{129}Xe

Estimation of relative detection sensitivity. A quantitative interpretation of the observed SNR of HP propane and HP ^{129}Xe requires consideration of several factors beyond the measured nuclear polarization. Following our previously reported treatment of HP-MRI detection sensitivity,[4] the image SNR can be represented schematically as

$$\mathrm{SNR} = ACV_{\mathrm{vox}}\, P\, \gamma, \qquad \text{(S1)}$$

where C is the concentration of the observed spins, V_{vox} is the voxel volume, P is nuclear-spin polarization, γ is the gyromagnetic ratio, and A represents the remaining experimental sensitivity factors, including RF-coil sensitivity, frequency-dependent noise, receiver performance, and pulse-sequence efficiency.

Because the propane and ^{129}Xe images were acquired using the same nominal FOV (256 × 256 mm²), matrix (64 × 64), slice thickness (50 mm), and flip angle (5°), the nominal voxel-volume contribution cancels in a first-order comparison. The remaining factors are considered individually below.

20.1. Number of hyperpolarized nuclei and polarization

Hyperpolarized propane was produced by pairwise addition of p-H_2, introducing two hyperpolarized protons per propane molecule. The polarization of these nascent protons was estimated to be approximately 1%. Thus, expressed per propane molecule,

$$(NP)_H = 2 \times 0.01 = 0.020 \qquad \text{(S2)}$$

The xenon used in these experiments was natural-abundance xenon. The isotopic abundance of ^{129}Xe is approximately 26.4%. Xenon constituted approximately 50% of the administered Xe/N_2 mixture, and the measured ^{129}Xe polarization was approximately 10%. The corresponding relative polarized-spin concentration is therefore

$$(NP)_{Xe} = 0.50 \times 0.264 \times 0.10 = 0.0132. \qquad \text{(S3)}$$

Consequently,

$$\frac{(NP)_H}{(NP)_{Xe}} = \frac{0.020}{0.0132} = 1.52. \quad \text{(S4)}$$

Thus, despite the approximately tenfold higher polarization of ^{129}Xe, the combination of two hyperpolarized protons per propane molecule, essentially undiluted propane, and the 26.4% natural abundance of ^{129}Xe results in **approximately 1.52-fold more nominal polarized spins for propane** under the conditions employed here.

20.2. Gyromagnetic-ratio contribution to magnetization

For a specified spin polarization, magnetization scales with the nuclear magnetic moment and hence approximately with γ. The gyromagnetic-ratio ratio is

$$\frac{\gamma_H}{\gamma_{^{129}Xe}} \approx 3.62. \quad \text{(S5)}$$

Combining this with Eq. S4 gives the nominal magnetization ratio

$$\frac{M_H}{M_{Xe}} \approx 1.52 \times 3.62 = 5.50. \quad \text{(S6)}$$

Thus, based only on polarized-spin concentration and gyromagnetic ratio, the nominal HP propane magnetization is approximately 5.5-fold greater than that of the naturalabundance HP ^{129}Xe mixture used here.

20.3. Frequency-dependent RF detection sensitivity

At B_0 = 0.35 T, the approximate resonance frequencies are $\nu_H \approx$ 14.9 MHz and $\nu_{129Xe} \approx$ 4.1 MHz, giving

$$\frac{\nu_H}{\nu_{Xe}} \approx 3.62. \quad \text{(S7)}$$

The induced NMR voltage itself scales linearly with resonance frequency. However, SNR does not scale linearly because RF-coil resistance and thermal noise are also frequency dependent. The classical analysis of Hoult and Richards describes these dependencies and predicts the familiar frequency scaling of NMR sensitivity under specified coil-noiselimited conditions.[5]

When polarization/magnetization is treated explicitly as in Eqs. S2–S6, an approximate residual frequency-dependent sensitivity term of

$$\text{SNR} \propto \nu^{3/4} \quad \text{(S8)}$$

can be used for the present illustrative comparison. Therefore,

$$\left(\frac{\nu_H}{\nu_{Xe}}\right)^{3/4} = (3.62)^{3/4} \approx 2.62. \quad \text{(S9)}$$

Combining the polarized-spin, gyromagnetic-ratio, and frequency-dependent terms gives

$$\left(\frac{\text{SNR}_H}{\text{SNR}_{Xe}}\right)_{\text{intrinsic}} \approx 1.52 \times 3.62 \times 2.62 \approx 14.4. \quad \text{(S10)}$$

Thus, **for otherwise equivalent RF detection conditions**, this simplified treatment predicts an approximately 14-fold sensitivity advantage for HP propane.

Importantly, Eq. S8 represents an approximate scaling under the assumptions of the RFnoise model and is not universal. Our previous low-field studies demonstrated experimentally that appropriately optimized RF coils can exhibit substantially weaker frequency dependence.[4] Equation S10 should therefore be regarded as an illustrative sensitivity estimate rather than an exact prediction.

20.4. Approximate RF-coil geometry correction

The RF coils employed for the two experiments were substantially different. HP propane was detected using a proton knee coil with an estimated volume of approximately 3 L, whereas HP ^{129}Xe was detected using a clinical chest coil with an estimated volume of approximately 30 L.

For an approximate geometric comparison, if characteristic coil dimension *r* scales with coil volume as

$$r \propto V^{1/3}, \qquad \text{(S11)}$$

and receive sensitivity is approximated as inversely proportional to characteristic coil dimension, then

$$\frac{A_H}{A_{Xe}} \sim \left(\frac{V_{Xe}}{V_H}\right)^{1/3} = \left(\frac{30}{3}\right)^{1/3} = 10^{1/3} \approx 2.15. \qquad \text{(S12)}$$

Applying this illustrative geometric correction to Eq. S10 gives

$$\left(\frac{\mathrm{SNR}_H}{\mathrm{SNR}_{Xe}}\right)_{\mathrm{estimated}} \approx 14.4 \times 2.15 \approx 31. \qquad \text{(S13)}$$

This correction is intentionally approximate. Coil sensitivity cannot be determined from coil volume alone and also depends on coil geometry, filling factor, conductor losses, *Q*, sample loading, matching, preamplifier noise, and the receive chain. Equation S13 therefore should not be interpreted as a fully quantitative prediction of the measured SNR ratio.

20.5. Comparison with experimentally observed SNR

The initial HP propane and HP ^{129}Xe lung images yielded approximate SNR values of 28 and 13, respectively. Thus,

$$\left(\frac{\mathrm{SNR}_H}{\mathrm{SNR}_{Xe}}\right)_{\mathrm{observed}} = \frac{28}{13} \approx 2.15. \qquad \text{(S14)}$$

The observed ~2.2-fold ratio is substantially smaller than the illustrative ~31-fold estimate in Eq. S13. Importantly, the preceding calculation implicitly makes two assumptions that are particularly unfavorable for an accurate description of HP propane.

Polarization loss before imaging. The ~1% propane polarization used in Eq. S2 represents the estimated initial polarization and does not necessarily represent the polarization remaining when the first image is acquired. In the excised lung, the measured propane decay time was only T_d = 0.68 ± 0.12 s, compared with T_1 = 10.9 ± 2.2 s for ^{129}Xe. Therefore, even a relatively short delay associated with gas transfer, lung inflation, and initiation of image acquisition can substantially reduce propane polarization while producing comparatively little loss of ^{129}Xe polarization. This distinction between polarization generated initially and polarization available at the time of imaging is important in quantitative HP MRI sensitivity comparisons and was considered explicitly in our previous HP MRI sensitivity studies.[4]

Incomplete NMR visibility of propane hyperpolarization. The calculation also assumes that the polarization of both *p*-H_2-derived protons is completely represented as directly observable magnetization. At 0.35 T, however, the chemical-shift frequency separation of the relevant propane protons is approximately 6.7 Hz, comparable to their scalar coupling (~7 Hz). The system therefore approaches the strong-coupling regime, where hyperpolarized spin order cannot necessarily be represented as independent longitudinal proton magnetizations. A fraction of the hyperpolarization may reside in long-lived or otherwise NMR-invisible spin-order components and consequently may not contribute directly to the GRE image signal.

A more realistic expression for the propane contribution can therefore be written schematically as

$$P_{H,\mathrm{effective}} = P_{H,0}\, e^{-t_{\mathrm{delay}}/T_d}\, f_{\mathrm{visible}}, \qquad \text{(S15)}$$

where P_H,0 is the estimated initial propane polarization, t_{delay} represents the effective interval between polarization generation and imaging, and $f_{visible} \le 1$ represents the fraction of the remaining hyperpolarized spin order that contributes to directly observable magnetization under the imaging conditions.

Neither t_{delay} nor $f$visible was independently quantified in the present experiments. Additionally, ^{129}Xe T_2* is anticipated to be greater than that of HP propane gas (in part due to lower gyromagnetic ratio), which would benefit SNR of HP ^{129}Xe disproportionately more when compared to HP propane gas. We therefore do not use Eq. S15 to fit or reconcile the observed SNR ratio. Instead, it illustrates why the nominal sensitivity estimate in Eq. S13 represents an idealized comparison rather than a prediction of experimental image SNR.

20.6. Comparison under equivalent gas concentration and RF detection conditions

It is also informative to compare 1%-polarized HP propane and 10%-polarized natural-abundance ^{129}Xe assuming equal gas number density and otherwise equivalent RF detection conditions. In this case, the 50% Xe dilution factor employed in Eq. S3 is omitted. The relative polarized-spin concentration is therefore

$$\frac{2(0.01)}{0.264(0.10)} = 0.758. \qquad \text{(S16)}$$

Including the gyromagnetic-ratio and approximate frequency-dependent RF-sensitivity terms employed above gives

$$\frac{\mathrm{SNR}_H}{\mathrm{SNR}_{Xe}} \approx 0.758(3.62)(3.62)^{3/4} \approx 7.2. \qquad \text{(S17)}$$

Thus, under these idealized equal-concentration and equivalent-detection conditions, 1%-polarized HP propane is estimated to provide approximately sevenfold greater instantaneous detection sensitivity than 10%-polarized natural-abundance ^{129}Xe. This comparison does not account for differences in hyperpolarization lifetime, T_2*, or the fraction of HP propane spin order that is directly NMR-visible.

21. References used in Supporting Information (SI)

1. Ariyasingha, N. M.; Samoilenko, A.; Chowdhury, M. R. H.; Nantogma, S.; Oladun, C.; Birchall, J. R.; Bawardi, T.; Salnikov, O. G.; Kovtunova, L. M.; Bukhtiyarov, V. I.; Shi, Z.; Luo, K.; Tan, S.; Koptyug, I. V.; Goodson, B. M.; Chekmenev, E. Y. Developing Hyperpolarized Butane Gas for Ventilation Lung Imaging. *Chemical & biomedical imaging* **2024,** *2* (10), 698-710.
2. Ariyasingha, N. M.; Oladun, C.; Samoilenko, A.; Chowdhury, M. R. H.; Nantogma, S.; Shi, Z.; Luo, K.; Tan, S.; Salnikov, O. G.; Xhupi, A.; Suleiman, M.; Chekmenev, Y. A.; Kovtunova, L. M.; Koptyug, I. V.; Gelovani, J. G.; Goodson, B. M.; Chekmenev, E. Y. Parahydrogen-Hyperpolarized Propane-d(6) Gas Contrast Agent: T(1) Relaxation Dynamics and Pilot Millimeter-Scale Ventilation MRI. *The journal of physical chemistry. A* **2025,** *129* (19), 4275-4287.
3. Chowdhury, M. R. H.; Oladun, C.; Ariyasingha, N. M.; Samoilenko, A.; Bawardi, T.; Burueva, D. B.; Salnikov, O. G.; Kovtunova, L. M.; Bukhtiyarov, V. I.; Shi, Z.; Luo, K.; Tan, S.; Gelovani, J. G.; Koptyug, I. V.; Goodson, B. M.; Chekmenev, E. Y. Rapid lung ventilation MRI using parahydrogen-induced polarization of propane gas. *Analyst* **2024,** *149* (24), 5832-5842.
4. Coffey, A. M.; Kovtunov, K. V.; Barskiy, D.; Koptyug, I. V.; Shchepin, R. V.; Waddell, K. W.; He, P.; Groome, K. A.; Best, Q. A.; Shi, F.; Goodson, B. M.; Chekmenev, E. Y. High-resolution Low-field Molecular MR Imaging of Hyperpolarized Liquids. *Anal. Chem.* **2014,** *86* (18), 9042–9049.
5. Hoult, D. I.; Richards, R. E. The signal-to-noise ratio of the nuclear magnetic resonance experiment. *J. Magn. Reson.* **1976,** *24* (1), 71-85.
